# A Cytochrome P450‐Mediated Intramolecular Carbon–Carbon Ring Closure in the Biosynthesis of Multidrug‐Resistance‐Reversing Lathyrane Diterpenoids

**DOI:** 10.1002/cbic.201600316

**Published:** 2016-07-15

**Authors:** Andrew J. King, Geoffrey D. Brown, Alison D. Gilday, Edith Forestier, Tony R. Larson, Ian A. Graham

**Affiliations:** ^1^ Centre for Novel Agricultural ProductsDepartment of BiologyUniversity of YorkHeslingtonYorkYO10 5DDUK; ^2^ Department of ChemistryUniversity of ReadingWhiteknightsReadingRG6 6ADUK

**Keywords:** casbene, cytochromes, diterpenoids, gene clusters, lathyranes

## Abstract

The Euphorbiaceae produce a wide variety of bioactive diterpenoids. These include the lathyranes, which have received much interest due to their ability to inhibit the ABC transporters responsible for the loss of efficacy of many chemotherapy drugs. The lathyranes are also intermediates in the biosynthesis of range of other bioactive diterpenoids with potential applications in the treatment of pain, HIV and cancer. We report here a gene cluster from *Jatropha curcas* that contains the genes required to convert geranylgeranyl pyrophosphate into a number of diterpenoids, including the lathyranes jolkinol C and *epi*‐jolkinol C. The conversion of casbene to the lathyranes involves an intramolecular carbon–carbon ring closure. This requires the activity of two cytochrome P450s that we propose form a 6‐hydroxy‐5,9‐diketocasbene intermediate, which then undergoes an aldol reaction. The discovery of the P450 genes required to convert casbene to lathyranes will allow the scalable heterologous production of these potential anticancer drugs, which can often only be sourced in limited quantities from their native plant.

The Euphorbiaceae produce a diverse range of casbene (**1**)‐derived diterpenoids,^1^ many of which are providing interesting leads in the development of new pharmaceuticals. These include the lathyranes (Scheme 1), which are inhibitors of ABC transporters that are responsible for the efflux of chemotherapy drugs in multidrug‐resistant (MDR) cancers as well as fungal and protozoal pathogens.^2^ The lathyranes are also the precursors of many other active diterpenoids including ingenol mebutate, a licenced pharmaceutical used for the treatment of actinic keratosis, prostratin, a lead compound for the treatment of latent HIV infections, and resiniferatoxin, an ultrapotent capsaicin analogue that is currently in clinical trial for the treatment of cancer‐related intractable pain (Scheme 2).^3^ Although the relationship between casbane, the lathyrane and a number of other diterpenoid classes was noted several decades ago (Scheme 1),^4^ the mechanism leading to the ring closure required to convert the 14:3 casbane ring into the 5:11:3 lathyrane ring system has not previously been reported.

**Scheme 1:**
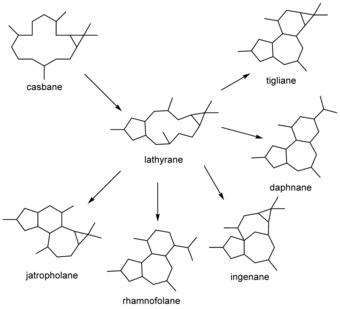
The lathyranes as proposed intermediates in the biosynthesis of diterpenoids with tigliane, daphnane, ingenane, rhamnofolane and jatropholane carbon skeletons.

**Scheme 2:**
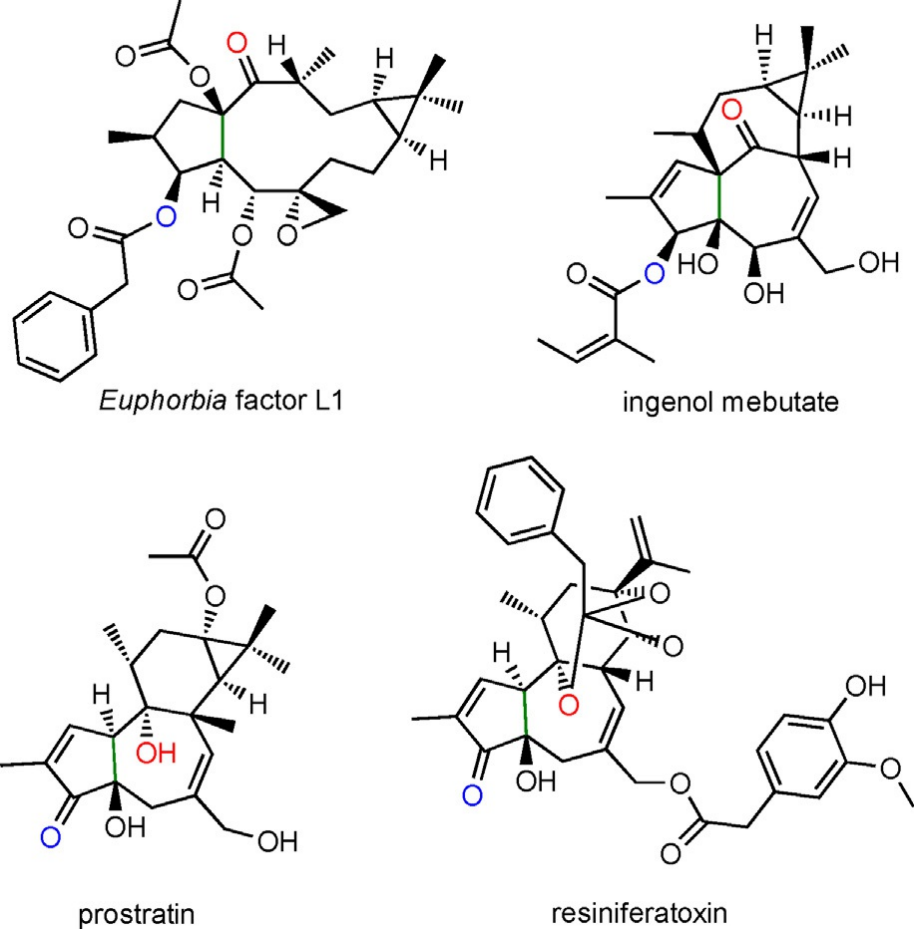
Structures of *Euphorbia* factor L1 (a lathyrane), ingenol mebutate (an ingenane), prostratin (a tigliane) and resiniferatoxin (a daphnane). The red and blue oxygen atoms highlighted on each of the molecules correspond to the 5 and 9 positions of casbene, respectively. The green carbon–carbon bond corresponds to the 6 and10 positions of casbene.

Recently, we reported a diterpenoid biosynthetic gene cluster in castor (*Ricinus communis*) that contained genes encoding diterpene synthases and several cytochrome P450s, including casbene synthases and casbene‐5‐oxidases. We also demonstrated the existence of similar clusters in other Euphorbiaceae including *Jatropha curcas*, a plant that produces a variety of diterpenoids including lathyranes, jatropholanes, rhamnofolanes and tiglianes^5^ (Figure S1 in the Supporting Information). Using a recently released version of the *Jatropha curcas* genome,^6^ we were able to perform further in silico analysis of this cluster, and found that it contained a number of enzyme‐encoding genes, including casbene synthases, cytochrome P450s, alcohol dehydrogenases and “alkenal reductase”‐like genes (Figure 1). The P450 genes were all members of the CYP71D tribe, and all but two were part of the CYP726A taxon‐specific bloom found so far only in the Euphorbiaceae^7^, ^8^ (Figure S2).

**Figure 1:**
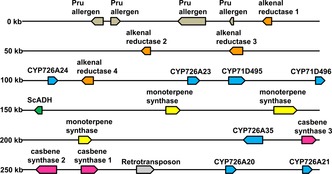
A diterpenoid biosynthesis gene cluster. The diagram corresponds to a 300 kb region present on scaffold 123 of the *J. curcas* genome (Genbank accession no. NW_012124159). Different classes of enzymes have been colour coded, for example, cytochrome P450 genes are shown in blue.

Using qPCR, we analysed the expression of the genes present within this cluster (Figure S3). The majority of the genes for which we were able to detect transcripts were most abundantly expressed within the roots. The exception to this was CYP71D495, which was most abundant in leaves, but still abundant in both stems and roots. This observation was consistent with the roots of *J. curcas* being rich in diterpenoids.^5^

Phylogenetic analysis of the P450 genes suggested that CYP726A35 was orthologous to CYP726A18 and CYP726A15 from castor (Figure S2). The former of these P450s is able to convert casbene into 5‐ketocasbene via a hydroxyl intermediate, whereas the latter catalyses a similar reaction with neocembrene.^7^ When CYP726A35 was transiently coexpressed with casbene synthase in *Nicotiana benthamiana* leaves, we were able to detect a metabolite (**2**) with a molecular mass of 302.23 (Figure 2), which we identified by NMR spectroscopy as 6‐hydroxy‐5‐ketocasbene (Scheme 3). This diterpenoid has previously been reported to be a product of casbene oxidation by CYP726A14 from castor.^9^

**Figure 2:**
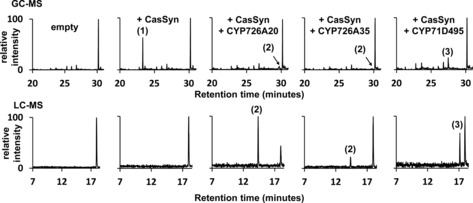
GC and LC chromatograms of casbene and casbene metabolites produced by transient expression of casbene synthase and casbene synthase with a single cytochrome P450 from the *J. curcas* gene cluster in *N. benthamiana*. The structures of the numbered metabolites are shown in Scheme 3. The corresponding mass and NMR spectra are provided in the Supporting Information and Figure S6.

**Scheme 3:**
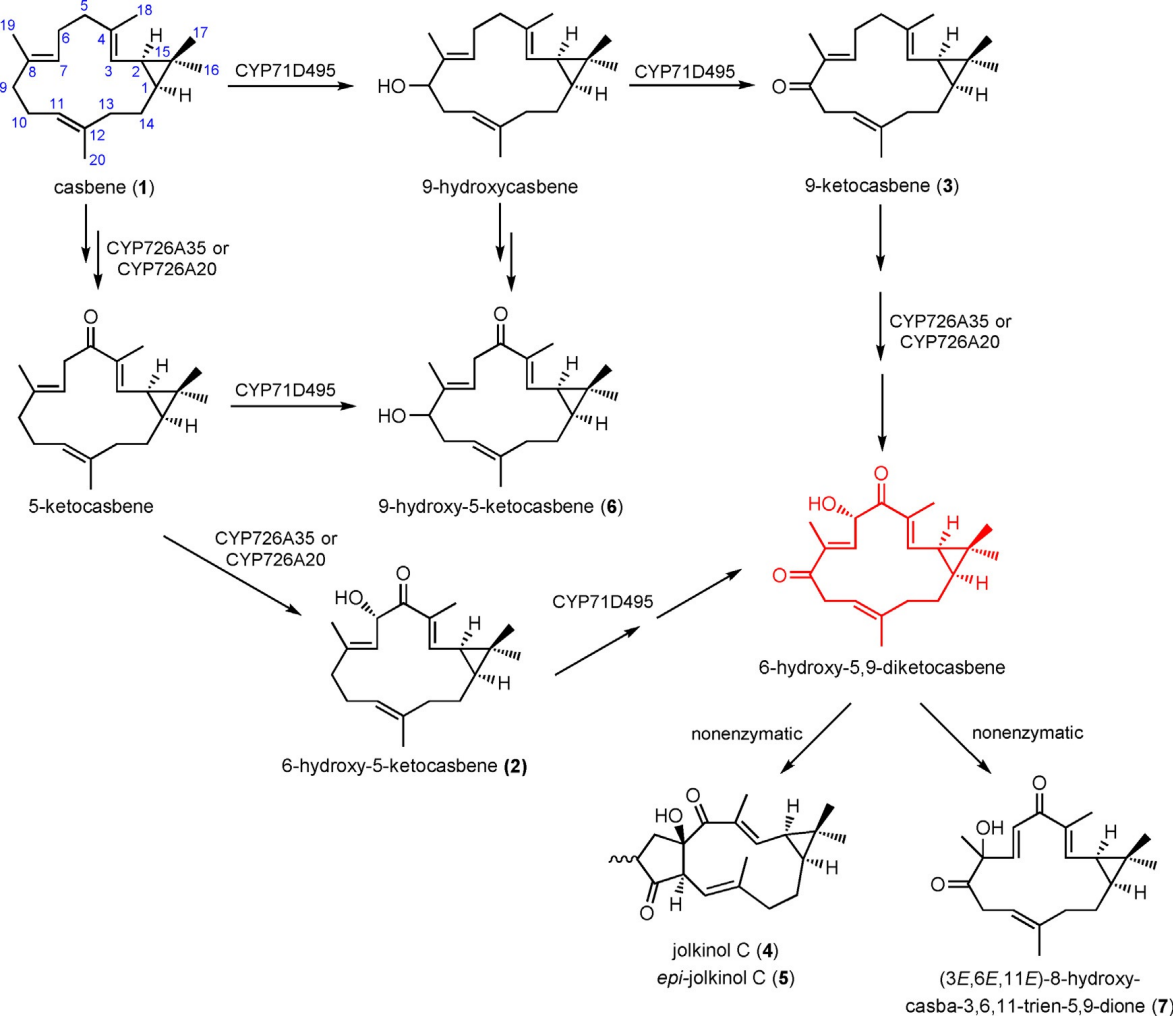
Roles of CYP726A35, CYP726A20 and CYP71D495 in diterpenoid biosynthesis. The blue numbering is based on the casbene numbering system.

A second P450 from *J. curcas* (CYP726A20) was also able to convert casbene into 6‐hydroxy‐5‐ketocasbene. This observation was similar to what we observed in castor, where we identified more than one P450 gene that was able to perform casbene 5‐oxidation.^7^ In silico analyses of CYP726A35, CYP726A18 and CYP726A15 (neocembrene‐5‐oxidase) revealed the presence of a putative plastidial transit peptide (Figure S4). Fusion of green fluorescent protein (GFP) to the N terminus resulted in the import of transiently expressed GFP into the plastids of *N. benthamiana* (Figure S4). CYP726A20 did not contain a predicted chloroplast transit peptide, and, consistent with this, fusion of the first 80 amino acids of this protein to GFP did not result in import into plastids. Thus it would appear that in both *Jatropha* and castor the enzymes catalysing casbene 5‐oxidation are located in both the plastid and the endoplasmic reticulum. Both *Jatropha* enzymes were also able to catalyse 6‐hydroxylation. Interestingly, in castor, *Euphorbia peplus* 7 and *J. curcas* (Figure 1), the plastidial casbene‐5‐oxidases are adjacent to a casbene synthase, thus indicating the order of these genes may be conserved in the Euphorbiaceae.

Only one other of the cytochrome P450 genes present within the *J. curcas* gene cluster (CYP71D495) was able to form a product (**3**) with casbene. This was purified and determined to be 9‐ketocasbene (Figure 2, Scheme 3). Interestingly, all of the diterpenoids reported in *J. curcas* (Figure S1) and the vast majority of those described in the Euphorbiaceae contain either a hydroxy or keto group at this 9‐position.^5^, ^7^ Indeed, 9‐oxidation (in addition to 5‐oxidation) appears to be present in all lathyranes, jatropholanes, tiglianes and ingenanes; this suggests that it might be a prerequisite for 6,10 ring closure.

Transient expression in *N. benthamiana* of casbene synthase together with both a casbene 5,6‐oxidase (either CYP726A35 or CYP726A20) and the casbene‐9‐oxidase (CYP71D495) resulted in the formation of several products (**4**–**7**; Figure 3). To increase the quantities of these products and facilitate purification for their subsequent identification by NMR spectroscopy, we co‐infiltrated *N. benthamiana* with *Agrobacterium* cultures harbouring plasmids for the overexpression of deoxyxylulose‐5‐phosphate synthase (DXS) and geranylgeranyl pyrophosphate synthase (GGPPS). Overexpression of these two 2‐*C*‐methyl‐d‐erythritol 4‐phosphate pathway genes has previously been shown to increase diterpenoid biosynthesis in *Agrobacterium*‐infiltrated *N. benthamiana*.^10^ Interestingly, the ratio of the main products (**4**, **5** and **6**) varied depending on whether CYP726A35 or CYP726A20 was used as a casbene 5,6‐oxidase; this might be due in part to their respective plastidial and ER localisation.

**Figure 3:**
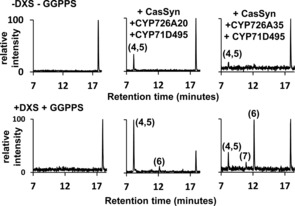
LC chromatograms obtained from co‐expression of casbene synthase with two cytochrome P450s from the *J. curcas* diterpenoid gene cluster. The lower panels show the results from co‐expression of the *J. curcas* genes with 1‐deoxy‐d‐xylulose 5‐phosphate synthase (DXS) and a plastidial geranylgeranyl pyrophosphate synthase (GGPPS) from *Arabidopsis thaliana*. The structures of the numbered metabolites are shown in Scheme 3. The corresponding mass and NMR spectra are provided in the Supporting Information and Figure S6.

NMR analysis (Supporting Information) indicated that one of LC peaks (**4, 5**) corresponded to a mixture of epimers with the lathyrane skeleton, the most abundant of which (ca. 80 %) has previously been described in the literature as jolkinol C (**4**).^11^ We have therefore called the other *epi‐*jolkinol C (**5**). The other two compounds were 9‐hydroxy‐5‐ketocasbene (**6**), and (3*E*,6*E*,11*E*)‐8‐hydroxy‐casba‐3,6,11‐trien‐5,9‐dione (**7**) (Scheme 3). Therefore, none of the four compounds isolated (**4**–**7**) corresponded to the product that might have been predicted from both oxidations, that is, 6‐hydroxy‐5,9‐diketocasbene (shown in red in Schemes 3 and 4). A likely explanation for the absence of this compound is that it undergoes spontaneous conversion to jolkinol C (**4**). In such an event, it is proposed that the 5‐keto group in 6‐hydroxy‐5,9‐diketocasbene undergoes facile enolisation to produce an extended enolate. Further tautomerisation of this extended enolate would allow the Δ^7,8^ double bond to migrate to the Δ^6,7^ position, thereby producing 5,6,9‐triketocasbene as a reactive intermediate, which would immediately transform into jolkinol C through an intramolecular aldol reaction, forming a new carbon–carbon bond between the 6 and 10 positions (Scheme 4). The isolation of both 8‐methyl epimers of jolkinol C, and indeed of similar pairs of epimers for several of the jatropholanes and rhamnofolanes in the roots of *J. curcas* 5 (Figure S1), is also consistent with such a nonenzymatic tautomerisation process, which effects double‐bond migration, prior to the aldol reaction.

**Scheme 4:**
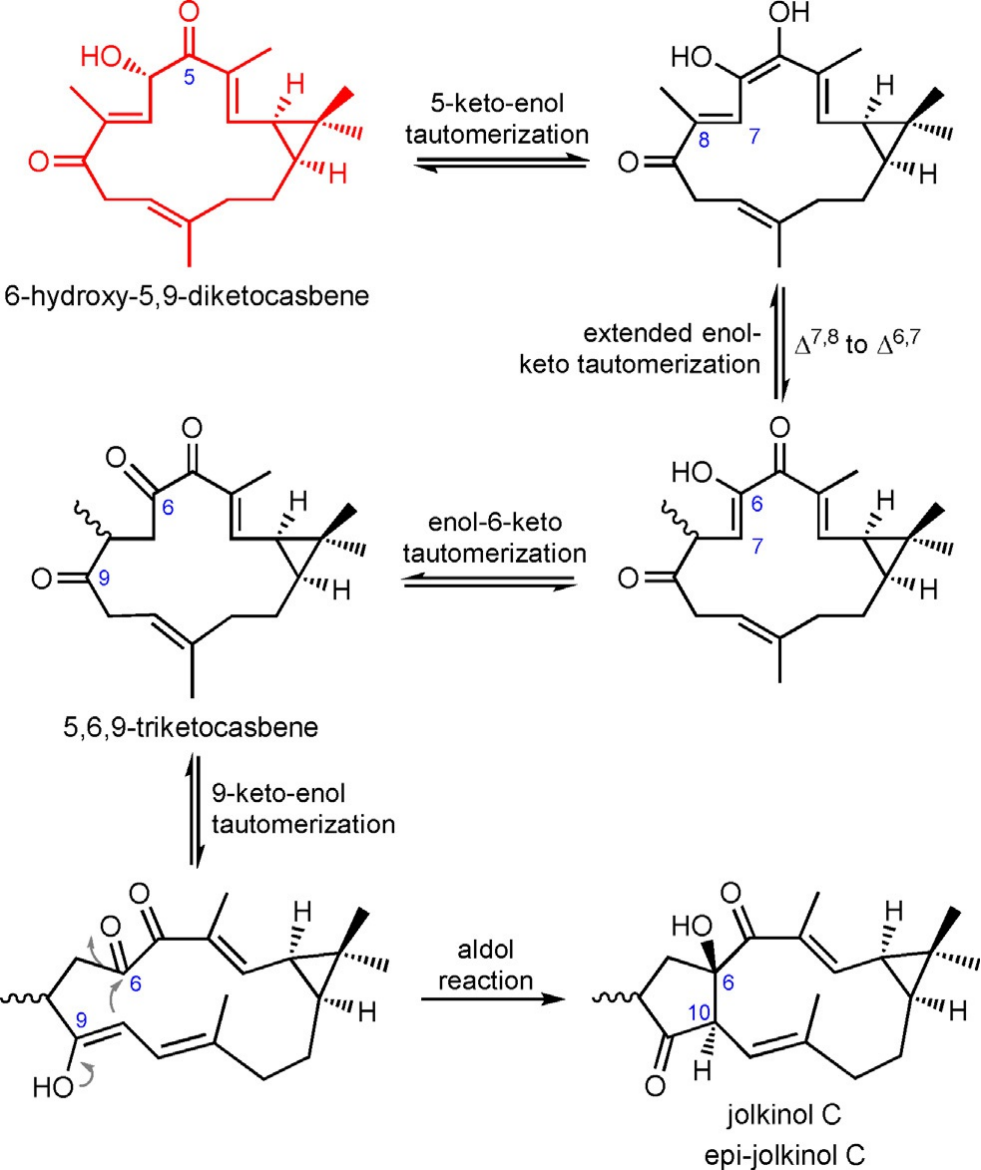
Presumed facile enolisation at the 5‐keto group is the key step for the Δ^7,8^ to Δ^6,7^ double bond isomerisation in 6‐hydroxy‐5,9‐diketocasbene, which leads to a triketo precursor that spontaneously converts to jolkinol C through an intramolecular aldol reaction.

A number of enzymes, including polyketide synthases, have previously been reported to perform intramolecular ring closures through aldol reactions.^12^ A number of plant P450s that catalyse intramolecular rearrangements have also been characterised. These include CYP80F1 from *Hyoscyamus niger*, which catalyses an oxidation and rearrangement as well as *Papaver somniferum* CYP719B1 and *Coptis japonica* CYP80G2, both of which catalyse C−C bond formations in alkaloid biosynthesis.^13^ In the case of the taxane diterpenoids, P450s catalysing C−C bond migrations and ring closures have also been reported.^14^ However, for the plant terpenoids at least, we are not aware of any other P450‐mediated ring closures involving an aldol reaction.

Our discovery of the steps required to convert casbene into lathyranes could be used to allow scalable production of the lathyranes in heterologous host systems such as yeast or tobacco. Interestingly, jolkinol derivatives have already received interest as precursors for the development of semisynthetic lathyranes with increased potency against MDR cancer cell lines.^15^ Our study has also provided insight into the metabolic diversification of the diterpenoids in the Euphorbiaceae, and demonstrated by the fact that the cytochrome P450s are not only responsible for decoration of the terpene scaffold, but also for initiating intramolecular ring closures. We are continuing to characterise the remaining genes present within the *J. curcas* diterpenoid cluster and to determine their involvement in the biosynthesis of casbene‐derived diterpenoids that have been reported from this plant.

## Supporting information

As a service to our authors and readers, this journal provides supporting information supplied by the authors. Such materials are peer reviewed and may be re‐organized for online delivery, but are not copy‐edited or typeset. Technical support issues arising from supporting information (other than missing files) should be addressed to the authors.

Supplementary
