# Supplementary material for: A Cytochrome P450‐Mediated Intramolecular Carbon–Carbon Ring Closure in the Biosynthesis of Multidrug‐Resistance‐Reversing Lathyrane Diterpenoids
Source: Chembiochem. 2016 Jul 15;17(17):1593–7. doi: 10.1002/cbic.201600316 (PMC5095812; doi:10.1002/cbic.201600316)
Supplement: Supplementary file 1 — Supplementary [file CBIC-17-1593-s001.pdf]

CHEMBIOCHEM

Supporting Information

A Cytochrome P450-Mediated Intramolecular Carbon–Carbon Ring Closure in the Biosynthesis of Multidrug-Resistance-Reversing Lathyrane Diterpenoids

Andrew J. King,*[a] Geoffrey D. Brown,[b] Alison D. Gilday,[a] Edith Forestier,[a] Tony R. Larson,[a] and Ian A. Graham*[a]

cbic_201600316_sm_miscellaneous_information.pdf

Supporting information

Experimental procedures.

Supporting Figure S1: Casbene-derived diterpenoids previously isolated from *Jatropha curcas*

Supporting Figure S2: Phylogenetic tree of CYP71 clan proteins

Supporting Figure S3: Analysis of expression of the diterpenoid cluster genes by qPCR

Supporting Figure S4: Plastidial transit peptide prediction by ChloroP

Supporting Figure S5: Plastidial localization of CYP726A35

Supporting Figure S6: Determination of molecular formulae of diterpenoids by high resolution mass spectrometry.

Supporting Figure S7: Gblocks curated 285 amino acid alignment for CYP71 clan proteins

Supporting Figure S8 – NMR data for 6-hydroxy-5-keto-casbene.

Supporting Figure S9 – NMR data for 9-keto-casbene.

Supporting Figure S10 – NMR data for 9-hydroxy-5-keto-casbene.

Supporting Figure S11 – NMR data for jolkinol C and *epi*-jolkinol C.

Supporting Figure S12 – NMR data for (3*E*, 6*E*, 11*E*)-8-hydroxy-casba-3,6,11-trien-5,9-dione

Supporting Table S1: Sequences of primers used for qPCR analysis of gene expression on *J. curcas* genome scaffold 123.

Supporting Table S2: Sequences of primers used insertion of *J. curcas* cDNA sequences into AgeI and XhoI sites of pEAQ-HT vector

Supporting Table S3: Primers used for creation of GFP fusion constructs in pEAQ-HT via Gibson Assembly

References to supporting information

Experimental procedures

Analysis of gene expression by qPCR.

RNA extraction, DNase treatment and cDNA synthesis were performed as described previously using three biological and four technical replicates per tissue[1]. qPCR primers (Supporting Table S1) were designed using Primer3Plus[2], and their specificity verified by a blastN search against the *J. curcas* genome. Optimal annealing temperatures were determined empirically by gradient PCR. qPCR reactions were then performed as described previously[1] and expression levels normalised against an β-actin gene (Genbank accession XM_012232498) using the delta-delta CT method[3] with correction for amplification efficiencies obtained using LinReg PCR[4].

Phylogenetic tree construction.

Construction of the phylogenetic tree of the CYP71 clan proteins (Supporting Figure S2) was constructed as described previously[1]. The curated 285 amino acid alignment is provided in Supporting Figure S7.

Gene cloning and transient gene expression in *Nicotiana benthamiana*.

cDNA was synthesised using total RNA from *J. curcas* roots or *A. thaliana* seedlings using g Superscript II reverse transcriptase (Invitrogen, Carlsbad, CA) and a 5'-$T_{(18)}$VN-3' primer. The open reading frame for each gene was then amplified and inserted into the pEAQ-HT expression vector via conventional restriction enzyme or Gibson cloning using the primers detailed in Supporting Table S2. In each instance, a 5'-AAAA-3' Kozak sequence was included immediately upstream of the start codon. DNA assembly was then performed using NEB Gibson Assembly Mastermix (NEB, Ipswich, MA) according to the manufacturer's protocol. After confirming the presence of the correct inserts by Sanger sequencing, the expression vectors were transformed into *Agrobacterium tumefaciens* LBA4404 using the freeze-thaw method[5]. For initial experiments to detect the production of novel diterpenoids, leaves were infiltrated with syringes with equal mixtures *A. tumefaciens* cultures at a final OD 600_{nm} of 1.0 in infiltration buffer (10 mM $MgCl_2$, 200 µM acetosynringone and 0.015% Silwet L-77). Five days after infiltration, ca. 2 cm^2 of leaf material was extracted with 1 ml of ethyl acetate by grinding for 1 minute with a steel bead at 30 Hz for 2 minutes in a Retsch homogenizer. After centrifugation, the supernatant was used either directly for GC-MS, or for LC-MS analysis after removal of the ethyl acetate and redissolving the extract in methanol. For the preparation of compounds for NMR analysis, multiple plants were infiltrated by immersing in cultures resuspended in infiltration buffer and then applying a partial vacuum to a pressure of 100 mbar for 1 minute.

In-silico analysis of plastidial transit peptides and creation of eGFP fusion constructs and visualization of subcellular localization.

In-silico prediction of plastidial transit peptides was performed using ChloroP[6]. pEAQ-HT expression vectors containing the N-terminal portions of proteins and eGFP were created by Gibson assembly using the primers detailed in Supporting Table S3. Leaves from *N. benthamiana* plants were examined by confocal microscopy five days after infiltration. A 20X magnification was used. Chlorophyll autofluorescence was observed using an excitation wavelength of 561 nm and an emission wavelength of 633-735 nm. GFP fluorescence was observed using an excitation wavelength of 488 nm and an emission wavelength of 495-600 nm.

Preparation and identification of 6-hydroxy-5-keto-casbene (2).

23.8 g of freeze-dried leaf material that had been infiltrated with *Agrobacterium* strains containing harboring plasmids for overexpression of casbene synthase and casbene 5,6-oxidase (CYP726A35) was extracted once with 250 ml ethyl acetate and once with 100 ml of ethyl acetate. The ethyl acetate was removed by rotary evaporation to yield 1.30 g of a green oily residue which was taken up in 10 ml of n-hexane. The extract was then applied to a 40 g Grace Resolve silica column and fractions collected on a 0-50 % ethyl acetate in hexane gradient. Fractions containing the desired product were pooled and then further purified using C30 reversed-phase HPLC as described previously[1] to yield ca. 1 mg of metabolite.

Data for 6-hydroxy-5-keto-casbene (2) :^{1}H NMR (700 MHz, $CDCl_3$): δ 6.35 (d, J = 11 Hz, 1H (H-3)), 5.25 (d, J = 9 Hz, 1H (H-6)), 5.09 (d, J = 9 Hz, 1H (H-7)), 4.84 (dd, J = 9, 4 Hz, 1H (H-11)), 2.25 (m, 1H (H-10a)), 2.24 (m, 1H (H-13a)), 2.20 (m, 1H (H-9a)), 2.14 (m, 1H (H-9b)), 2.12 (m, 1H (H-14a)), 2.03 (m, 1H (H-10b)),1.96 (s, 3H (H-18)), 1.77 (ddd, J = 12, 10, 3 Hz (H-13b)), 1.70 (s, 3H (H-19)), 1.58 (s, 3H (H-20)), 1.56 (dd, J = 11, 8 Hz, 1H (H-2)), 1.21 (ddd, J = 12, 8, 2 Hz, 1H (H-1)),1.18 (s, 3H (H-16)), 1.02 (s, 3H (H-17)), 0.84 (dddd, J = 12, 12, 10, 3 Hz (H-14b)); ^{13}C NMR (175 MHz, $CDCl_3$): δ 200.2 (C-5), 145.2 (C-3), 142.2 (C-8), 136.3 (C-12), 134.2 (C-4), 124.2 (C-7), 123.8 (C-11), 68.4 (C-6), 39.8 (C-13), 38.7 (C-9), 35.8 (C-1), 29.2 (C-16), 28.2 (C-2), 27.5 (C-15), 25.9 (C-14), 23.9 (C-10), 16.0 (C-17), 15.5 (C-19), 15.4 (C-20), 12.0 (C-18); HRMS (m/z): $[M+H]^+$ calcd. for $C_{20}H_{30}O_2$, 303.2319; found, 303.2313.

Preparation and identification of 9-keto casbene (3).

19.32 g of freeze-dried leaf material that had been infiltrated with *Agrobacterium* strains containing harboring plasmids for overexpression of casbene synthase and casbene 9-oxidase (CYP71D495) was extracted with ethyl acetate as described above. The ethyl acetate was removed by rotary evaporation to yield 1.09 g of a green oily residue which was taken up in 10 ml of n-hexane. The extract was then subjected to normal –phase silica flash chromatography and C30 reversed-phase HLPC as described above to yield 770 µg of metabolite.

Data for 9-keto-casbene (3): ^{1}H NMR (700 MHz, $CDCl_3$): δ 6.55 (dd, J = 7, 7 Hz, 1H (H-7)), 5.12 (dd, J = 8, 6 Hz, 1H (H-11)), 4.80 (d, J = 10 Hz, 1H (H-3)), 3.56 (dd, J = 12, 8 Hz, 1H (H-10a)), 3.02 (dd, J = 12, 6 Hz, 1H (H-10b)), 2.41 (m, 2H, (H-6a/6b)), 2.32 (m, 2H (H-5a and H-13a)), 2.11 (ddd, J = 13, 7, 7 Hz, 1H (H-5b)), 1.93 (dd, J = 12, 12 Hz, 1H (H-13b)), 1.85 (ddd, J = 14, 5, 1 Hz, 1H (H-14a)), 1.77 (s, 3H (H-20)), 1.75 (s,

3H (H-19)), 1.74 (s, 3H (H-18)), 1.29 (dd, J = 10, 9 Hz, 1H (H-2)), 1.12 (dddd, J = 14, 12, 10, 3 Hz, 1H (H-14b)), 1.08 (s, 3H (H-16)), 0.87 (s, 3H (H-17)), 0.68 (ddd, J = 10, 9, 1 Hz, 1H (H-1)); ^{13}C NMR (175 MHz, $CDCl_3$): δ 202.0 (C-9), 144.5 (C-7), 138.1 (C-12), 135.6 (C-8), 132.6 (C-4), 123.1 (C-3), 119.7 (C-11), 40.4 (C-13), 40.1 (C-10), 38.9 (C-5), 31.5 (C-1), 29.2 (C-16), 26.5 (C-2), 26.0 (C-6), 24.2 (C-14), 20.8 (C-15), 17.7 (C-20), 15.8 (C-18), 15.5 (C-17), 11.0 (C-19); HRMS (m/z): $[M+H]^+$ calcd. for $C_{20}H_{30}O$, 287.2369; found, 287.2368.

Preparation and identification of 9-hydroxy-5-keto-casbene (6), jolkinol C (4) , *epi*-jolkinol C (5) and (3*E*, 6*E*, 11*E*)-8-hydroxy-casba-3,6,11-trien-5,9-dione (7).

13.8 g of freeze-dried leaf material that had been infiltrated with *Agrobacterium* strains containing harboring plasmids for overexpression of deoxy-xylulose-5-phosphate synthase, geranylgeranyl pyrophosphate synthase, casbene synthase, casbene 5,6-oxidase (CYP726A35) and casbene-9-oxidase (CYP71D495) was extracted with ethyl acetate as described above. The ethyl acetate was removed by rotary evaporation to yield 600 mg of a green oily residue which was taken up in 10 ml of n-hexane. The extract was then subjected to normal–phase silica flash chromatography using a 10% to 100% ethyl acetate in hexane gradient. Fractions containing the desired metabolites were then further purified using preparative C18 reversed-phase HPLC to yield ca. 1.17 mg of 9-hydroxy-5-keto-casbene, 450 µg of jolkinol C (ca. 1:4 mixture of epimers) and 740 µg of 8-hydroxy-5,9-diketocasbene. To obtained improved NMR data for *epi*-Jolkinol C, 6.1 g of freeze-dried leaf material that had been infiltrated with *Agrobacterium* strains containing harboring plasmids for overexpression of deoxy-xylulose-5-phosphate synthase, geranylgeranyl pyrophosphate synthase, casbene synthase, casbene 5,6-oxidase (CYP726A20) and casbene-9-oxidase (CYP71D495) was extracted with ethyl acetate to yield 480 mg of green oily residue. After preparative chromatography, we were able to isolate a 690 µg fraction enriched in *epi*-jolkinol C (ca 65 % of *epi*-Jolkinol C).

Data for 9-hydroxy-5-keto-casbene **(6)**: ^{1}H NMR (700 MHz, $CDCl_3$): δ 6.33 (d, J = 10 Hz, 1H (H-3)), 5.29 (dd, J = 9, 4 Hz, 1H (H-7)), 4.69 (dd, J = 9, 4 Hz, 1H (H-11)), 4.17 (dd, J = 8, 6 Hz, 1H (H-9)), 3.69 (dd, J = 14, 9 Hz, 1H (H-6a)), 2.95 (dd, J = 14, 4 Hz, 1H, (H-6b)), 2.29 (m, 2H (H-10a/10b)), 2.18 (ddd, J = 10, 10, 10 Hz, 1H (H-13a)), 2.10 (dddd, J = 15, 12, 10, 3 Hz, 1H (H-14a)), 1.88 (s, 3H (H-18)), 1.74 (dd, J = 11, 11 Hz, 1H (H-13b)), 1.62 (s, 3H (H-20)), 1.57 (s, 3H (H-19)), 1.50 (dd, J = 10, 9 Hz, 1H (H-2)), 1.17 (s, 3H (H-16)), 1.16 (ddd, J = 12, 9, 3 Hz, 1H (H-1)), 1.10 (s, 3H (H-17)), 0.81 (ddd, J = 12, 12, 12 Hz, 1H (H-14b)); ^{13}C NMR (175 MHz, $CDCl_3$): δ 199.4 (C-5), 143.3 (C-3), 139.0 (C-8), 138.3 (C-12), 137.1 (C-4), 120.7 (C-7), 119.4 (C-11), 76.9 (C-9), 40.1 (C-13), 38.6 (C-6), 35.0 (C-1), 31.6 (C-10), 29.0 (C-16), 27.6 (C-2), 26.2 (C-14), 25.9 (C-15), 15.9 (C-17), 15.3 (C-20), 11.7 (C-18), 11.6 (C-19); HRMS (m/z): $[M+H]^+$ calcd. for $C_{20}H_{30}O_2$, 303.2319; found, 303.2313.

Data for Jolkinol C **(4)**: ^{1}H NMR (700 MHz, $CDCl_3$): δ 7.36 (d, J = 12 Hz, 1H (H-3)), 5.35 (d, J = 10 Hz, 1H (H-11)), 3.51 (dd, J = 14, 9 Hz, 1H (H-7a)), 3.03 (d, J = 10 Hz, 1H (H-10)), 2.66 (br d, J = 13 Hz, 1H (H-13a)), 2.58 (dq, J = 9, 7 Hz, 1H, (H-8)), 2.19 (ddddd, J = 14, 4, 4, 2, 2 Hz, 1H (H-14a)), 1.86 (s, 3H (H-18)), 1.69 (ddd, J = 13,12, 2 Hz, 1H (H-13b)), 1.59 (dd, J = 14, 2 Hz, 1H (H-7b)), 1.57 (dddd, J = 14, 12, 12, 2 Hz, 1H (H-14b)), 1.46 (dd, J = 12, 8 Hz, 1H (H-2)) 1.38 (s, 3H (H-20)), 1.29 (d, J = 7 Hz, 3H (H-19)), 1.19 (s, 3H (H-16)), 1.14 (ddd, J = 12, 8, 3 Hz, 1H (H-1)), 1.09 (s, 3H (H-17)); ^{13}C NMR (175 MHz, $CDCl_3$): δ 219.73 (C-9), 198.15 (C-5), 152.18 (C-3), 144.91 (C-12), 132.33 (C-4), 118.79 (C-11), 88.65 (C-6), 57.99 (C-10), 40.43 (C-7), 38.98 (C-8), 35.87 (C-13), 35.72 (C-1), 29.86 (C-2), 29.17 (C-16), 27.65 (C-14), 25.28 (C-15), 20.89 (C-20), 18.39 (C-19), 16.25 (C-17), 12.16 (C-18); HRMS (m/z): $[M+H]^+$ calcd. for $C_{20}H_{28}O_3$, 317.2111; found, 317.2096. N.B. The lathyrane system is not used; the casbane numbering system has been retained to allow comparison with precursor molecules.

Data for *epi*-Jolkinol C **(5)** (characterized as a *ca* 65:35 mixture with Jolkinol C (**4**)): ^{1}H NMR (700 MHz, $CDCl_3$): δ 7.34 (d, J = 12 Hz, 1H (H-3)), 5.29 (d, J = 12 Hz, 1H (H-11)), 2.86 (d, J = 11 Hz, 1H (H-10)), 2.64 (1H, (H-13a)), 2.61 (1H (H-7a)), 2.61 (1H (H-8)), 2.18 (1H (H-14a)), 2.15 (1H (H-7b)), 1.85 (3H (H-18)), 1.71 (1H, (H-13b)), 1.54 (1H (H-14b)), 1.45 (1H (H-2)), 1.43 (s, 3H (H-20)), 1.20 (d, J = 7 Hz, 3H (H-19)), 1.19 (3H (H-16)), 1.13 (1H (H-1)), 1.08 (3H (H-17)); ^{13}C NMR (175 MHz, $CDCl_3$): δ 219.11 (C-9), 198.38 (C-5), 151.82 (C-3), 145.10 (C-12), 132.32 (C-4), 118.83 (C-11), 86.77 (C-6), 56.93 (C-10), 41.47 (C-7), 40.30 (C-8), 36.00 (C-13), 35.44 (C-1), 29.83 (C-2), 29.18 (C-16), 27.85 (C-14), 25.23 (C-15), 20.74 (C-20), 16.25 (C-17), 14.61 (C-19), 12.09 (C-18).

Data for (3*E*, 6*E*, 11*E*)-8-hydroxy-casba-3,6,11-trien-5,9-dione **(7)**: ^{1}H NMR (700 MHz, $CDCl_3$): δ 6.52 (d, J = 17 Hz, 1H (H-6)), 6.49 (d, J = 17 Hz, 1H (H-7)), 6.22 (d, J = 9 Hz, 1H (H-3)), 5.21 (dd, J = 8, 6 Hz, 1H (H-11)), 3.42 (dd, J = 15, 6 Hz, 1H (H-10a)), 3.35 (br s, -OH), 3.23 (dd, J = 15, 8 Hz, 1H, (H-10b)), 2.37 (ddd, J = 14, 8, 8 Hz, 1H (H-13a)), 2.15 (dddd, J = 15, 8, 8, 3 Hz, 1H (H-14a)), 1.89 (s, 3H (H-18)), 1.88 (ddd, J = 14, 9, 3 Hz (H-13b)), 1.70 (s, 3H (H-20)), 1.53 (s, 3H (H-19)), 1.48 (dd, J = 10, 9 Hz, 1H (H-2)), 1.19 (s, 3H (H-17)), 1.14 (ddd, J = 10, 8, 2 Hz, 1H (H-1)), 0.99 (s, 3H (H-16)), 0.93 (m, 1H (H-14b)); ^{13}C NMR (175 MHz, $CDCl_3$): δ 209.2 (C-9), 194.3 (C-5), 144.0 (C-7), 142.8 (C-3), 140.9 (C-12), 138.5 (C-4), 128.9 (C-6), 116.5 (C-11), 79.2 (C-8), 39.2 (C-13), 39.1 (C-10), 32.9 (C-1), 29.0 (C-17), 27.4 (C-2), 25.6 (C-15), 24.8 (C-14), 23.7 (C-19), 16.2 (C-20), 16.1 (C-16), 12.4 (C-18); HRMS (m/z): $[M+H]^+$ calcd. for $C_{20}H_{28}O_3$, 317.2111; found, 317.2107.

Supporting Figure S1 – Casbene-derived diterpenoids previously isolated from *Jatropha curcas*

a

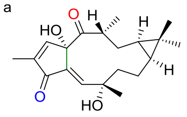

11 Curculathyrane A

b

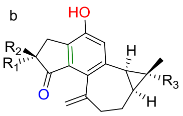

R_1 = H, R_2=CH_3, R_3 = CH_3 = **12** Jatropholone A
R_1 = CH_3, R_2=H, R_3 = CH_3 = **13** Jatropholone B
R_1 = H, R_2=CH_3, R_3 = CHO = **14** Jatrophaldehdye
R_1 = CH_3, R_2=CH_3, R_3 = CHO = **15** *epi*-Jatrophaldehyde
R_1 = H, R_2=CH_3, R_3 = CH_2OH = **16** Jatrophol
R_1 = CH_3, R_2=H, R_3 = CH_2OH = **17** *epi*-Jatrophol

c

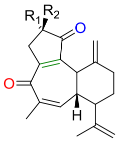

R_1 = H, R_2=CH_3 = **18** Curcusone A
R_1 = CH_3, R_2=H = **19** Curcusone B
R_1 = OH, R_2=CH_3 = **20** Curcusone C
R_1 = CH_3, R_2=OH = **21** Curcusone D

d

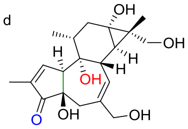

22 12-deoxy-16-hydroxy-phorbol

Supporting Figure S1. Diterpenoids of the **(a)** lathyrane **(b)** jatropholane **(c)** rhamnofolane and **(d)** tigliane classes which have been isolated from *J. curcas*. The red and blue oxygen atom highlighted on each of the molecules corresponds to the 5- and 9-positions of casbene repectively. The carbon-carbon bond highlighted in green corresponds to the 6,10-positions of casbene.

Supporting Figure S2 – Phylogenetic tree of CYP71 clan proteins

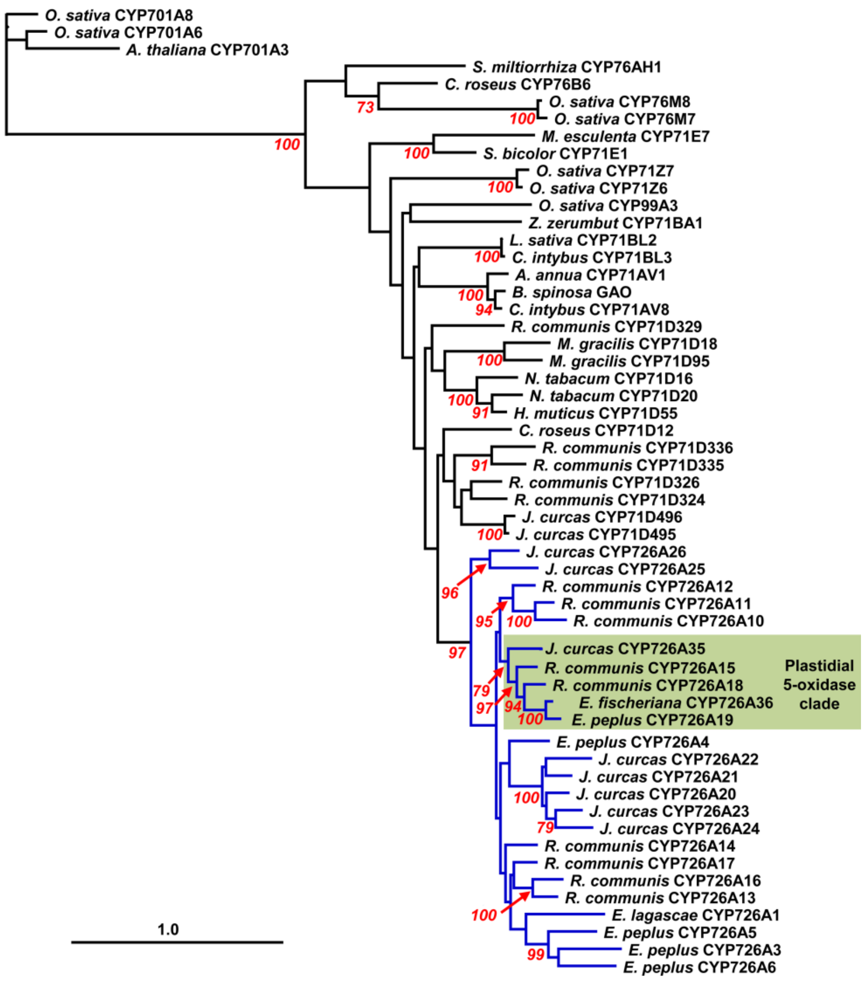

Supporting Figure S2: Phylogenetic tree of the CYP71 clan, including *Ricinus communis* casbene-5-oxidases (CYP726A14 [KF986810], CYP726A17 [KF986813], and CYP726A18 [KF986814]), neocembrene-5-oxidase (CYP726A15 [KF986811]), 5-keto-casbene-7,8-epoxidase (CYP726A16 [KF986812]), CYP726A10 [XP_002532302], CYP726A11 [XP_002532303], CYP726A12 [XP_002532304], CYP726A13 [KF986809],CYP71D324 [XP_002522762], CYP71D326 [XP_002522721], CYP71D335 [XP_002514827.1] and CYP71D336 [XP_002514826]; *Euphorbia lagascae* Δ-12 fatty acid epoxidase (CYP726A1 [AAL62063]); *Euphorbia peplus* casbene-5-oxidase CYP726A19 [KF986826], CYP726A3 [KF986822], CYP726A4 [KF986823], CYP726A5 [KF986824] and CYP726A6 [KF986825]; *Euphorbia fischeriana* CYP726A36 [JL056356]; *Jatropha curcas* CYP726A20 [KF986815], CYP726A21 [KF986816], CYP726A22 [KF986817], CYP726A23 [KF986818], CYP726A24 [KF986819], CYP726A25 [KF986820], CYP726A26 [KF986821], CYP726A35 [KX060558], CYP71D495 [KX060559] and CYP71D496 [KX060560]; *Catharanthus roseus* tabersonine-16-hydroxylase (CYP71D12 [ACM92061]) and geraniol-10-hydroxlase (CYP76B6, [Q8VWZ7]; *Nicotiana tabacum* 5-epiaristolochene dihydroxylase (CYP71D20 [Q94FM7]) and α-cembratrienol hydroxylase (CYP71D16 [AAD47832]; *Hyoscyamus muticus* premnaspirodiene oxidase (CYP71D55 [A6YIH8], *Mentha gracilis* limonene-6-hydroxylase (CYP71D18 [Q9XHE8] and limonene-3-hydroxylase (CYP71D95 [Q6WKY9]; *Zingiber zerumb*et α-humulene 10-hydroxlase (CYP71BA1 [E3W9C4]); *Oryza sativa* isokaurene C2-hydroxylase (CYP71Z6 [A3A871]),ent-cassadiene C2-hydroxylase (CYP71Z7 [Q6YV88]) ent-cassadiene C11-hydroxylases (CYP76M7 and CYP76M8 [Q69X58 and Q6YTF1] *syn*-pimaradiene oxidase (CYP99A3 [Q0JF01] and ent-kaurene oxidases (CYP701A6[AAT81230]) and CYP701A8[AAT46567], *Lactuca sativa* costunolide synthase (CYP71BL2 [F8S1I0]; *Cichorium intybus* (+)-valencene oxidase (CYP71AV8 [E1B2Z9] and costunolide synthase (CYP71BL3 [G3GBK0]; *Artemesia annua* amorpha-4,11-diene C-12 oxidase (CYP71AV1 [Q1PS23]*; Barnadesia spinosa* germacrene A oxidase (BsGAO, D5JBX1.1]; *Manihot esculenta* 2-methylbutanal oxime monooxygenase (CYP71E7, [Q6XQ14]); *Sorghum bicolor* 4-hydroxyphenylacetaldoxime monooxygenase (CYP71E1 [O48958]; *Salvia miltiorrhiza* miltiradiene oxidase (CYP76AH1 [AGN04215]), and *Arabidopsis e*nt-kaurene oxidase (CYP701A3 [NP_197962]. The branches highlighted in blue indicate the CYP726A tribe — a taxon specific bloom for the Euphorbiaceae. The green box indicates a clade containing CYP726A subfamily proteins predicted to contain a plastidial transit peptide at their N-termini. The numbers in red positioned to the left of and below the nodes indicate bootstrap values shown as a percentage based on 1000 replicates. Bootstrap values below 70% are not shown. The scale bar represents the number of substitutions per site.

Supporting Figure S3 – Analysis of expression of the diterpenoid cluster genes by qPCR

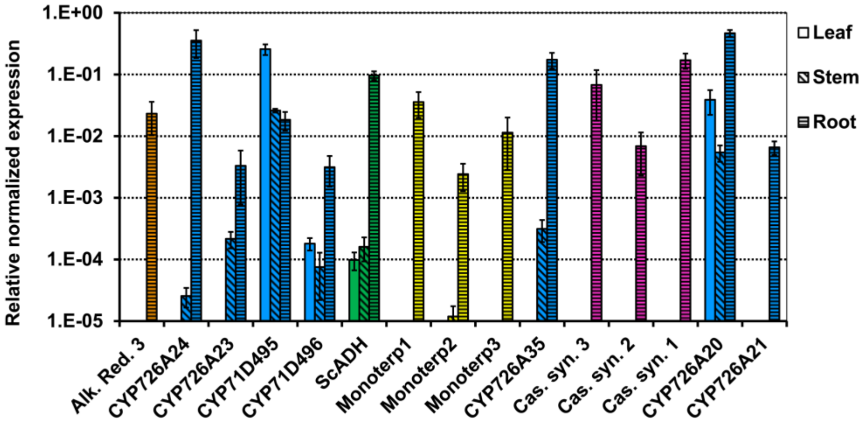

Supporting Figure S3: Analysis of expression in leaf, stem and root of the *J. curcas* diterpenoid cluster genes. The bars have been colour coded to match Figure 2. The error bars represent the standard deviations from three biological replicates. Expression levels are relative to β-actin. Genes for which no expression was detected are not shown.

Supporting Figure S4 – Plastidial transit peptide prediction by ChloroP

Species	Protein	Length	Score	cTP	CS-score	cTP-length
Ricinus communis	CYP726A15	534	0.586	Y	3.336	66
Ricinus communis	CYP726A18	533	0.568	Y	1.229	43
Euphorbia peplus	CYP726A19	560	0.586	Y	3.119	61
Jatropha curcas	CYP726A20	503	0.455	-	n/a	n/a
Jatropha curcas	CYP726A35	629	0.561	Y	1.527	73

>CYP726A15
MSLQPAPVSQSNFLYKKVPPILRAPTTKSSGSSRSSFFSSSVKLAARPPQPQACLSLNKNDDSNTSASSLPPGPWKLPLL
GNIHQLVGALPHHRLRDLAKAYGPVMSVKLGEVSAVVISSVDAAKEVLRTQDVNFADRPLVLAAEIVLYNRQDIVFGSYG
EQWRQMRKICTLELLSIKRVQSFKSVREEELSNFIRYLHSKAGTPVNLTHHLFSLTNSIMFRISIGKKYKNQDALLRVID
GVIEAGGGFSTADVFPSFKFLHHISGEKSSLEDLHREADYILEDIINERRASKINGDDRNQADNLLDVLLDLQENGNLEI
ALTNDSIKAAILEMFGAGSDTSSKTAEWALSELMRHPEEMEKAQTEVRQVFGKDGNLDETRLHELKFLKLVIKETLRLHP
PVALIPRECRQRTKVNGYDIDPKTKVLVNVWAISRDPNIWTEAEKFYPERFLHSSIDYKGNHCEFAPFGSGKRICPGMNL
GLTNLELFLAQLLYHFNWEFPDGITPKTLDMTESVGAAIKRKIDLKLIPVLFHP

>CYP726A18
MSSQPAVLQSNFLNRNVQPFLTIPSASTKYSGTACFSSFPSVKLNARPPQACFSLNKNNDHSTPTSILPPGPWQLPLIGN
IHQLVGHLPHSRLRDLGKIYGPVMSVQLGEVSAVVVSSVEAAKEVLRIQDVIFAERPPVLMAEIVLYNRHDIVFGSYGDH
WRQLRKICTLELLSLKRVQSFKSVREDEFSNFIKYLSSKAGTPVNLTHDLFSLTNSVMLRTSIGKKCKNQEAILRIIDSV
VAAGGGFSVADVFPSFKLLHMISGDRSSLEALRRDTDEILDEIINEHKAGRKAGDDHDEAENLLDVLLDLQENGDLEVPL
TNDSIKATILDMFGAGSDTSSKTAEWALSELMRHPEIMKKAQEEVRGVFGDSGEVDETRLHELKYLKLVIKETLRLHPAI
PLIPRECRERTKINGYDVYPKTKVLVNIWAISRDPNIWSEADKFKPERFLNSSLDYKGNYLEFAPFGSGKRVCPGMTLGI
TNLELILAKLLYHFDWKLPDGITPETLDMTESVGGAIKRRTDLNLIPVLYPTH

>CYP726A19
MATLQHSMQANLQKQNLHPLLNKSFGTPNRPSFVYSSKSASRRTIQACLSSNSQPGGVCPMANRFASSTTNQSVTESSSK
PDEEDENSPVKLPPGPWKLPLLGNILQLVGDLPHSRLRDLATEYGPVMSVQLGEVYAVVISSVEAAREILRNQDVNFADR
PPVLVSEIVLYNRQDIVFGAYGVHWRQMRRLCTTELLSIKRVQSFKLVREEEVSNFIKSLYSKAGKPVNLTEGLFTLTNS
IMLRTSIGKKCRDQDTLLRVIEGVVAAGGGFSIADVFPSAVFLHDINGDKSGLQSLRRDADLILDEIIGEHRAIRGTGGD
QGEADNLLDVLLDLQENGNLEVPLNDDSIKGAILDMFGAGSDTSSKSTEWALSELLRHPEEMKKAQDEVRRVFAKKGNVE
ESQLDQLKYLKLVIKETLRLHPAVPLIPRECREKTKVNGYDILPKTKALVNIWAISRDPKIWPEADKFIPERFENSSIDF
KGNNLEFAPFGSGKRICPGMALGITNLELFLAQLLYHFDWKLADGKDGRDLDMGEVVGGAIKRKVDLNLIPIPFHTSPAN

>CYP726A20
MEHQILSFPVLFSLLLFILVLLKVSKKLYKHDSKPPPGPWKLPFIGNLIQLVGDTPHRRLTALAKTYGPVMGVQLGQVPF
LVVSSPETAKEVMKIQDPVFAERPLVLAGEIVLYNRNDIVFGSYGDQWRQMRKFCTLELLSTKRVQSFRPVREEEVASFV
KLMRTKKGTPVNLTHALFALTNSIVARNAVGHKSKNQEALLEVIDDIVVSGGGVSIVDIFPSLQWLPTAKRERSRIWKLH
QNTDEILEDILQEHRAKRQATASKNWDRSEADNLLDVLLDLQQSGNLDVPLTDVAIKAAIIDMFGAGSDTSSKTAEWAMA
ELMRNPEVMKKAQEELRNFFGENGKVEEAKLHELKWIKLIIKETLRLHPAVAVIPRVCREKTKVYGYDVEPGTRVFINVW
SIGRDPKVWSEAERFKPERFIDSAIDYRGLNFELIPFGAGKRICPGMTLGMANLEIFLANLLYHFDWKFPKGVTAENLDM
NEAFGGAVKRKVDLELIPIPFRP

>CYP726A35
MSLQPAILQGNTCKQYFHPLSSISSTRWVGNCNRFAFLSPAKPTANRAPQASLSSKLQPVVRLLTKFPASGFLAMNQSVD
QFASTTTSLTKIFNKIGKPIQSSPFLVSVLLLMFMASKIQNQQEEDDNSINLPPGPWRLPFIGNIHQLAGPGLPHHRLTD
LAKTYGPVMGVHLGEVYAVVVSSAETSKEVLRTQDTNFAERPLVNAAKMVLYNRNDIVFGSFGDQWRQMRKICTLELLSV
KRVQSFKSVREEEMSSFIKFLSSKSGSPVNLTHHLFVLTNYIIARTSIGKKCKNQEALLRIIDDVVEAGAGFSVTDVFPS
FEALHVISGDKHKFDKLHRETDKILEDIISEHKADRAVSSKKSDGEVENLLDVLLDLQENGNLQFPLTNDAIKGAILDTF
GAGSDTSSKTAEWTLSELIRNPEAMRKAQAEIRRVFDETGYVDEDKFEELKYLKLVVKETLRLHPAVPLIPRECRGKTKI
NGYDIFPKTKVLVNVWAISRDPAIWPEPEKFNPERFIDNPIDYKSINCELTPFGAGKRICPGMTLGITNLELFLANLLYH
FDWKLPDGKMPEDLDMSESFGGAIKRKTDLKLIPVLARPLTPRNANSGNTFTTTDADSPASMCPHLKAL

Supporting Figure S4: Predication of plastid transit peptides using ChloroP. The predicted transit peptides are highlighted in green text.

Supporting Figure S5 – Plastidial localization of CYP726A35

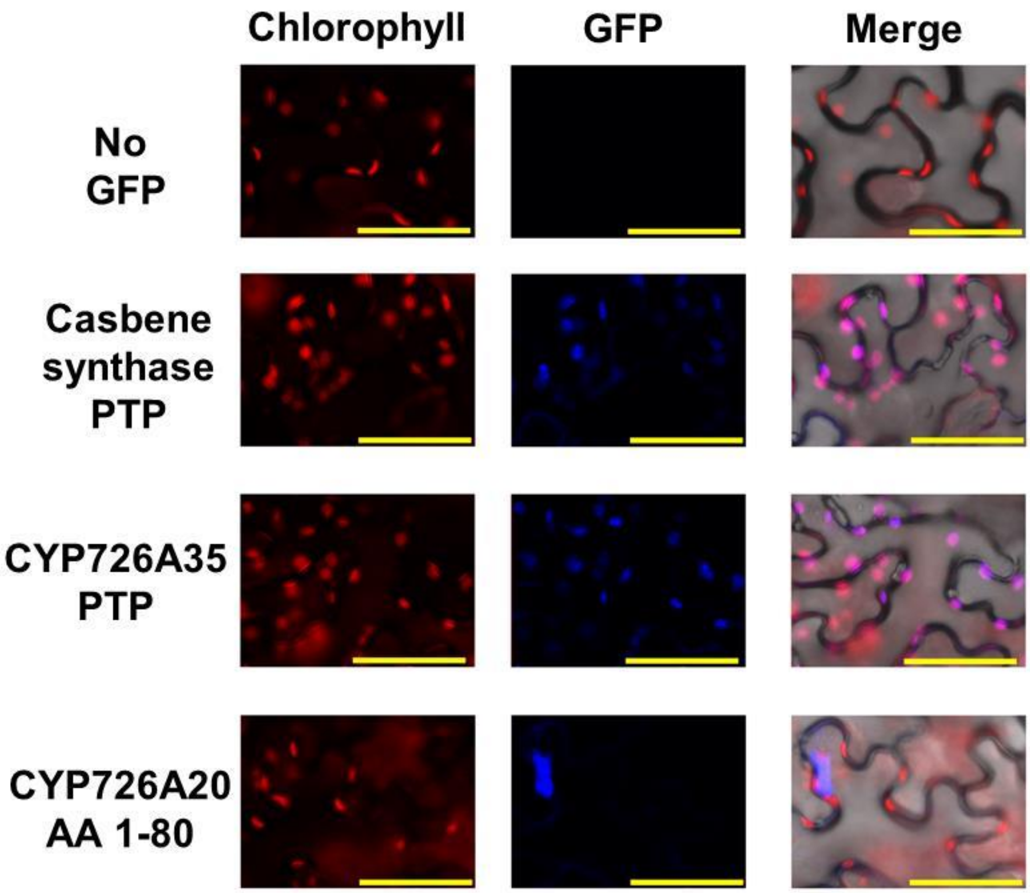

Supporting Figure S5: Transient expression of eGFP fusion proteins in the epidermis of *N. benthamiana***.** The first 72 amino acids from casbene synthase, the first 93 amino acids from CYP726A35, and the first 80 amino acids of CYP726A20 were fused to eGFP. The upper panel is a control experiment where *N. benthamiana* plants were infiltrated with an empty vector control. The left hand column shows the chlorophyll autofluorescence. The middle column shows the eGFP fluorescence. The right had column shows the two fluorescent merges with the bright field image showing epidermal (pavement) cells. The yellow bar in each picture corresponds to a distance of 50 μM. NB, when *N. benthamiana* plants are infiltrated using syringes, transgene expression is typically confined to the epidermal pavement cells. The diffuse red background fluorescence that appears in some images corresponds to mesophyll cells which are out of the focal plane.

Supporting Figure S6 – Determination of molecular formulae of diterpenoids by high resolution mass spectrometry.

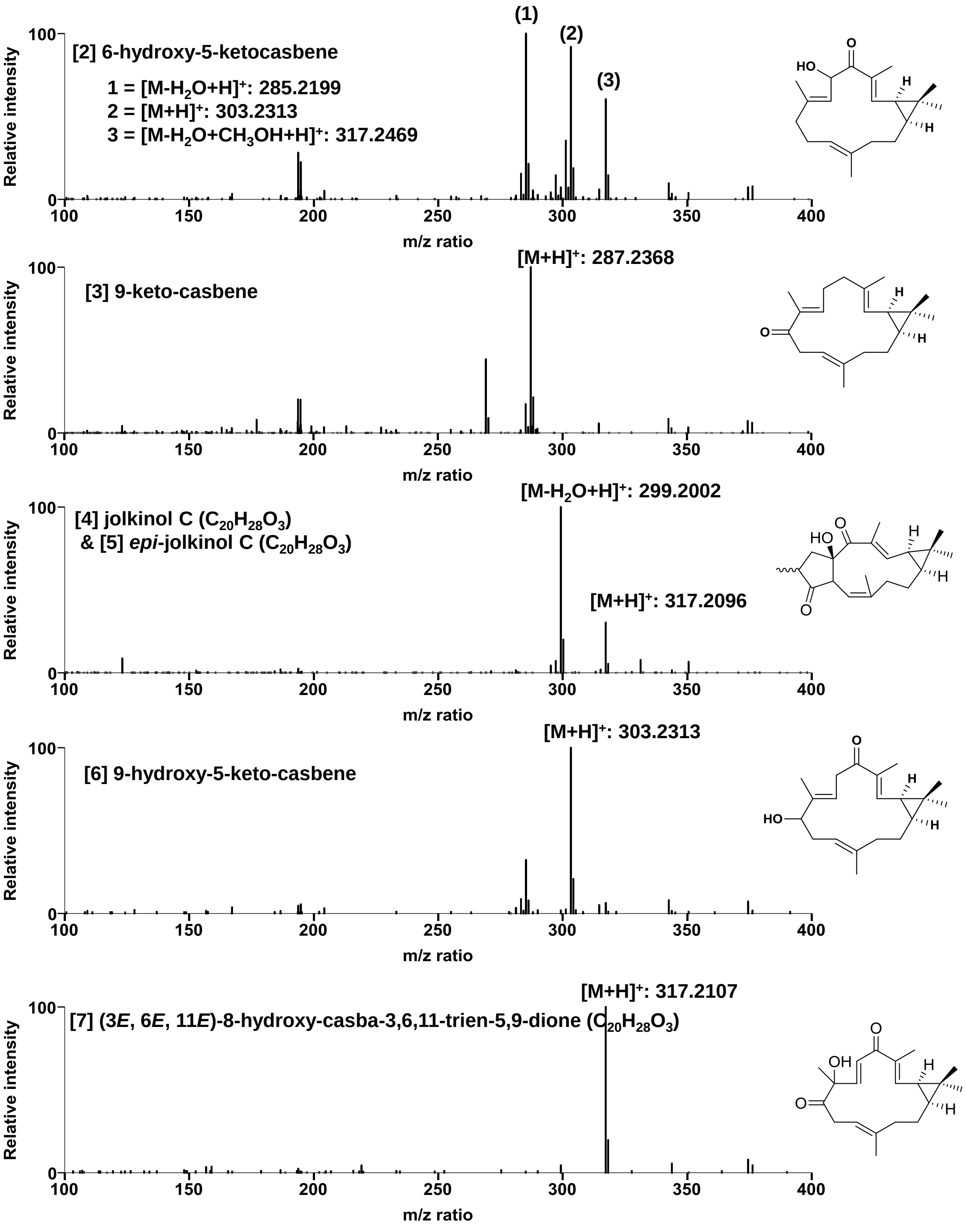

Supporting Figure S7 – Gblocks curated 285 amino acid alignment for CYP71 clan proteins

```
AthCYP701A3     1 VVPGFPVIGNLLQLPHKTFTRWSEIYGPIYSIKMGSSSLIVLNSTETAKEAMVTRFSSISTRKLSNAHKLVKRCLLNGLL
OsaCYP701A8     1 AVPGLPIIGNLHQLPHQTFAKWAEIYGPIYTIRTGASSVVVLNSTEVAKEAMVAKFSSISTRKLSKAHKMVKRYVMSSML
OsaCYP701A6     1 AVPGLPIIGNLHQLPHQTFAKWSETYGPIYTIKTGASPVVVLNSTEVAKEAMIDKFSSISTRKLPKAQKMAKRNIMIGML
SmiCYP76AH1     1 GPFPYPIVGNMLQLPHQVFAKLSKRYGPLMSIHLGSLYTVIVSSPEMAKEILHRHGQVFSGRTIAQAWRDMRKICKEQMF
CroCYP76B6      1 GPSPLPFIGSLHLLPHKSLAKLSKKHGPIMSLKLGQITTIVISSSTMAKEVLQKQDLAFSSRSVPNAWRSLRKVLNSNIF
OsaCYP76M7      1 GPTPLPLIGNLHLAFHHKLRDLARVHGPVMTLKLGLATNVVISSREAAIEAYTKYDRHLAARATPDTWKALRGIQGSHVF
OsaCYP76M8      1 GPTPLPLIGNLHLVFHHKLRDLARVHGPVMTLKLGLATNVVISSREAAIEAYTKYDRHLAARATPDTWKALRGIHASHVF
SbiCYP71E1      1 GPAQLPILGNLHLLPHKNLRELARRYGPVMQLRLGTVPTVVVSSAEAAREVLKVHDVDCCSRPASPGWREMRKLFALELL
MesCYP71E7      1 GPRQLPLIGNLHQLPYVDFWKMAKKYGPVMYLQLGRCPTVVLSSTETSKELMKDRDVECCSRPLSVGWREMRKLFIFELL
OsaCYP71Z6      1 GPWTLPLIGSLHHLTHRSLRALSEKHGPIMQLWMGEVPAVVVSSPAVAEEVLKHQDLRFADRHLTATWRHLRKICMQELL
OsaCYP71Z7      1 GPWTLPLIGSLHHLIHRSLRALSEKHGPIMQLWMGEVPAVIVSSPAVAEEVLKHQDLRFADRHLTATWRHLRKICMQELL
ZzeCYP71BA1     1 SPPRLPLIGNIHQLPHRILLQLARTHGPLICLRLGQVDQVVASSVEAVEEIIKRHDLKFADRPRDLTWKQMRKIYAMELL
OsaCYP99A3      1 GPWNLPLVGGLLHLPQVALRDLAGKYGPVMFLRTGQVDTVVISSPAAAQEVLRDKDVTFASRPSLLVWRMLRKLCTVELL
CciCYP71BL3     1 GPPKLPIIGNIHQLPHRVLRNLARKYGPIMHLQLGQVSTVVVSTPRLAREIMKTNDISFADRPTTTTWRQMKKICTLELL
LsaCYP71BL2     1 GPPKLPIIGNIHQLPHRVLRNLAKKYGPIMHLQLGQVSTVVVSTPRLAREIMKTNDISFADRPTTTTWRQMKKICTLELL
MgrCYP71D95     1 GPPKLPLVGHLHLLPQHAMADMAKKYGPVTHVQLGEVFSVVLSSREATKEAMKLLDPACADRFESIGWRQMRKICVSELL
MgrCYP71D18     1 SPPKLPVIGHLHFLPQHVFRSIAQKYGPVAHVQLGEVYSVVLSSAEAAKQAMKVLDPNFADRFDGIGWRQMRRICVTELL
RcoCYP71D329    1 GPMKLPIIGNLHNLPHHALQELAKEYGPILHLQLGEVSTMVVSDGKMAKEVLKTHDLNFAQRPRLLAYKQMKKVGAEELL
CroCYP71D12     1 GPPQIPILGNAHQLTHHILRDLAKKYGPLMHLKIGEVSTIVASSPQIAEEIFRTHDILFADRPSNLEWRQLRKISMMELL
AanCYP71AV1     1 EPWRLPIIGHMHHLPHRGVRDLARKYGSLMHLQLGEVPTIVVSSPKWAKEILTTYDITFANRPETLTWRQLRKICTLELL
CinCYP71AV8     1 EPWRLPIIGHMHHLPHRGVMELARKHGSLMHLQLGEVSTIVVSSPRWAKEVLTTYDITFANRPETLTWRQLRKLCTLELL
BspGAO          1 EAWRLPIIGHMHHLPHRGVTDMARKYGSLMHLQLGEVSTIVVSSPRWAKEVLTTYDITFANRPETLTWRQLRKLCTLELL
NtaCYP71D16     1 GPWKLPIIGSMLHMPHHVLRDLAKKYGPIMHLQLGEVSLVVISSPGMAKEVLKTHDLAFANRPLLVAWRQMRKICLLELL
HmuCYP71D55     1 GPWKLPLLGSMLHMPHHVLRDLAKKYGPLMHLQLGEVSAVVVTSPDMAKEVLKTHDIAFASRPKLLAWRQMRKICVLEVL
NtaCYP71D20     1 GPWKIPILGSMLHMPHHVLRDLAKKYGPLMHLQLGEISAVVVTSRDMAKEVLKTHDVVFASRPKIVAWRQMRKICVMELL
RcoCYP71D335    1 GPFQLPIIGNIHQLTHHRMRDLAEKYGPVMLLQVGDLTTIVISSAETAKQVLKTHDLFFAQRPNILAWRQLRKLCSLQLL
RcoCYP71D336    1 GPFQLPIIGNIHQLPHLRVTDLAKKYGPVMRLQLGEFTITVLSSAETAREVLKTHDLNFSQRPNLIGWRQLRKLCMLQLL
JcuCYP71D495    1 GPNKLPLIGNVHNLPYHRLRDLSKKYGPIMHLQLGENTTVVISSPELAQEVMKTHDVNFAQRPFVLAWRQLRKMCSLELL
JcuCYP71D496    1 GPNKLPVIGNVHNLPYHRLRDLSSKYGPIIHLQLGEITTVVISSPELAQEVMKTHDLNFAQRPFVLAWRQLRKMCSLELL
RcoCYP71D324    1 GPWKLPLIGSMHHLPHHRLKDLAKKYGPLMHLQLGELTNIVISSPEIAKEVMKTHDVVFAQRPHLLAWRQMRKLCTLELL
RcoCYP71D326    1 GPTKLPLIGNMHQLPHHRLRDLAKKYGSIMHLQLGEVPHVVVSSPEAAKEVMKTHDIVFAQRPFLLAWRQLRKICVLELL
EpeCYP726A3     1 GPWKLPLLGNILQLANHRLCDLAKVYGPVMSIQLGQNPAVVLSSPEAAEQVFKIQGDLFNNRPPALSWRQIRKITVMEFL
EpeCYP726A6     1 GPRKYPFIGNLPQLVHQRLADLAKTYGPVMSIQQGQIPSVVLSSVETAKEVLKIQGEEFAGRPSTMAWRQMKKISTLEFL
EpeCYP726A5     1 GPWQFPIIGNIPQLFHHRLSDLAKIHGPIMSIQQGQIPAVVITSVELAKEVLKTQGEIFAGRPQAPAWRQMRKICTLEFL
ElaCYP726A1     1 GPWKFPIIGNLPHLGHERFRALAQIYGPVMSLQIGQVSAVVISSAEAAKEVMKTQADAFAQRPIVLDWRQMKKIWILEFL
JcuCYP726A25    1 GPWRLPLIGNIHQLPHLRLTDLARTYGPVMRLQLGQIEAVVISSAETAKQVMKTQESQFLGRPSLLAWRQMKKIAVVELL
JcuCYP726A26    1 GPWKLPLIGNLHNLPHHRLRDLAGKYGAVMGFQLGQVPTVVISSAEIAKQVLKTHEFQFIDRPSLLAWRQIKKIAILELL
JcuCYP726A22    1 GPWKLPFLGNILQLPHHRFAELARTYGPVMGIKLGEIPFLVVSSPEAAKEVMKIQDPIFAERALVFAWRQLRKFCTLALL
JcuCYP726A24    1 GPWQLPFLGNIFQLFHIRLSELAKTYGPVMGIKVGQVPFLIVSSPEMAKEVLKVQDPTFVDRPVVLAWRQMRKFCTLELL
JcuCYP726A23    1 GPWKLPFLGNILQLFHRRLTELAKTHGPVMSINVGQIPYVVVSSPETAKEVMKIQDPVFADHPVVLALKQMRKFCTVELL
JcuCYP726A21    1 GPWKLPFLGNILQLPHRRITELAKKYGPVMSIKLGQHPYLVVSSPETAKEVMRTQDPIFADRPLVLAWRQMRKFCALELL
JcuCYP726A20    1 GPWKLPFIGNLIQLPHRRLTALAKTYGPVMGVQLGQVPFLVVSSPETAKEVMKIQDPVFAERPLVLAWRQMRKFCTLELL
JcuCYP726A35    1 GPWRLPFIGNIHQLPHHRLTDLAKTYGPVMGVHLGEVYAVVVSSAETSKEVLRTQDTNFAERPLVNAWRQMRKICTLELL
EpeCYP726A4     1 GPWKYPILGNIPQLPHHRLRDLAKIHGPVMNIQLGQVKSIVISSPETAKEVLKTQDIQFANRPLLLAWRQMRKICTLELL
RcoCYP726A14    1 GPWRLPLLGNFHQLPHHRLTELAKIYGPVMGIQLGQISVVIISSVETAKEVLKTQGEQFADRTLVLAWRQLRKLCTLELL
RcoCYP726A17    1 GPWKLPLLGNIHQLPHHRLRDLAKTYGPVMSIQLGQISAVVISSVQGAKEVLKTQGEVFAERPLIIAWRQMRKICTLELL
RcoCYP726A13    1 GPWKLPLIGNMHQLPHHRLRELAKTHGPVMSIQLGQVSAVVISSVEAAKQVLKTQGELFAERPSILAWRQMRKICTFELL
RcoCYP726A16    1 GPRKLPIIGNMHQLPHHPVTELSKTYGPIMSIQLGQISAIVISSVEGAKEVLKTQGELFAERPLLLAWRQLRKLCTLEVL
RcoCYP726A12    1 GPWKLPILGNIHQLPHHRLRDLAKIYGPVMSIKLGEVSAVVISSAEAAKEVLRTQDVSFADRPLGLSWRQLRKICILELL
RcoCYP726A10    1 GPWKLPLLGNIHQLPHQRLRDLAKSFGPVMSIKLGEISAVIISSAEAAQEVLKSQDVTFAERPASLAWRQTRKLCVLELL
RcoCYP726A11    1 GPWKLPFLGNIHQLPHRRLRDLAKTYGPVMSIKLGEISSIVISSAEAAQEVLKTQDVIFAERPIALALRQSRKICILELL
EpeCYP726A19    1 GPWKLPLLGNILQLPHSRLRDLATEYGPVMSVQLGEVYAVVISSVEAAREILRNQDVNFADRPPVLVWRQMRRLCTTELL
EfiCYP726A36    1 GPWKLPFIGNILQLPHRRLRDLATVYGPVMSVQLGEVYAVIISSVEAAKEVLRTQDVNFADRPPVLVWRQMRRICTMELL
RcoCYP726A15    1 GPWKLPLLGNIHQLPHHRLRDLAKAYGPVMSVKLGEVSAVVISSVDAAKEVLRTQDVNFADRPLVLAWRQMRKICTLELL
RcoCYP726A18    1 GPWQLPLIGNIHQLPHSRLRDLGKIYGPVMSVQLGEVSAVVVSSVEAAKEVLRIQDVIFAERPPVLMWRQLRKICTLELL
```

```
AthCYP701A3   81 GANAQKRKRHYRDALIENVSSKLNFRAIFEHELFGVALKQAFGEARIQQKHKRRLAVMNALIAILVWETIIETADTTLVT
OSACYP701A6   81 GTSAQKQFRDIRDMMIHNMLSTFIFRDVFKDELFRLSMIQSLGETRVFTTETRRTAVMRALLMMLVWEALIEAADTTLVT
OsaCYP701A8   81 GFNAQKQFRGTRERMISNVLSTLNFRDVYINELFSLSLIQSLGDTIVSTTEFRRDAIMNALLMLLLSESILAAADTVLVT
SmiCYP76AH1   81 SNQSMEASQGLRRQKLQQLLDHVDIREAAFITTLNLMSATLFSKRRADVFFGKLLAKIEGYFTHLMLDLFVGGSDTNTTS
CroCYP76B6    81 SGNRLDANQHLRTRKVQELIAYCDVGRAAFRTSLNLLSNLIFSRHRMTIHFGEVLKLFGGLIERMCLDLFVAGTDTTSST
OsaCYP76M7    81 TPRGLAAVRPIRERKVGDLMAYLLLGQAMHTGLLNLVSFSYFSRRWTTKRFNRVFSIMGDIVLDMLFEAFVAGADTMALT
OsaCYP76M8    81 TPRVLAAVRPIRERKVGDLIAYLLVGHAMYTGILNMVSFSYFSRRWTTKRFNRVFSIMGDIVLNMLFEAFVAGADTMALT
SbiCYP71E1    81 SMRRVKAACYAREQEMDRLVADLVLNDHVFALTDGIIGTVAFGLARRERIFNELDVFFEKVVKAIVLDTFIGAIDTSSVT
MesCYP71E7    81 SMRRVQTFWYAREEQMDKMIEILNLTEKVFNMMDGIIGTIAFGLAKRQRTFTDVDRYFEKVVKAILMNVFVGGIDTSAVT
OsaCYP71Z6    81 TAARVRSFQGVREREVARLVRELNLNERISKLANDIVMVSSVGPRKGLASRKRMELVIRRIIVVLLFDMISGASETSPTV
OsaCYP71Z7    81 TAARVRSFQGVREREVARLVRELNLNERISKLANDIVMVSSVGPRKGLASRKRMELVIRRIIVVLLFDMFTGASETSPTV
ZzeCYP71BA1   81 NSRRVKSFAAIREDVARKLTGEINLSEMVMSMINAIVIRVAFGKSKLEGVHGKLDKVFDEIVKAIVMEIFLAGTETSSSV
OsaCYP99A3    81 STKMVRQLAPIRDGETLALVRNITLATLLISCTNTFTAKAAFGRSRLERLRLQLDTVFDKIVKAIILDMFTGGTETTSST
CciCYP71BL3   81 SAKKVRSFSSIREEELSRISKVLNFTEMTVEMVNNVICKATLGKAKWLKMQKQLDDILEDVVKAVVLDMLTAGTDTSSAT
LsaCYP71BL2   81 SAKKVRSFSSIREEELRRISKVLNFTEMTVEMVNNVICKATLGKAKWLKMQKQLDDILEDVVKAVVLDMLTAGTDTSSAT
MgrCYP71D95   81 SARNVRSFGFIRQDEMSRLLRHLDMTERIATLTCSIICRAAFGKSKLWRMRRRVDTILEAIIKAFIFDTFSAGTETSSTT
MgrCYP71D18   81 SPKNVRSFGYIRQEEIERLIRLLDVTEEVSKMSCVVVCRAAFGKYRLQRMRRRLDHILDGFIKGFIFDTFSAGAETSSTT
RcoCYP71D329  81 GPKRVQSYSSLREEEVQKLVESVNFGDRICRLTNVIVFKAAFGKTKVKEVAKEIDRMLSDIIKAVIFDFFIAGTETSSNT
CroCYP71D12   81 SQKSVQSFRSIREEEVLNFIKSINLSKEISLLIYGITTRAAFGKYKIEKIHKQFDVIVETIIKAIIFNIFSAGTETSSTT
AanCYP71AV1   81 SVKKVKSFQSLREEECWNLVQEINLSENVFKLIATILSRAAFGRARLTSLRKKIDNLIDNLIKAIILDMFGAGTDTSSST
CinCYP71AV8   81 SNKKVKSFQSLREEECWNLVKDINLSENIFKMIATILSRAAFGRAKLTNIHNKLDNLINNIVKAVILDMFGAGTDTSSAT
BspGAO        81 SAKKVKSFQSLREEECWNLVKEVDLSESIFKLIATILSRAAFGRAKLTNIHNKLDSLINNIVKAVILDMFGAGTDTSSAT
NtaCYP71D16   81 SAKNVKSFNSIRQDEVHRMIKFFNVTKRISLFTNSMTCRSAFGKAKVMNTHNELDAILENIIKAIISDIFGGGTETSSTT
HmuCYP71D55   81 SAKNVRSFSSIRRDEVLRLVNFVNFTERLFLFTSSMTCRSAFGEGKIMKAHHKVDAIVEDVIKAIIFDMFAAGTETSSST
NtaCYP71D20   81 NAKNVRSFSSIRRDEVVRLIDSINFTQRIIWFASSMTCRSAFGKRKLLNAHLKVDAIVEDVIKAVIVDMFAAGTETSSTT
RcoCYP71D335  81 HVKRVQSFRPIREEEVSNIISVINLSELIRTFTYRLISRTAFGRIKVEKHFEKVDKKFQNIIKAVIMDMFFGGTDTSSAT
RcoCYP71D336  81 SAKRVQSFRTIREEEVSKLISSIHLRKLINALTFRIISRAAIGKVKLEKHFKQVDKIFQDIIKAVIMDMFIGGTSTSVEV
JcuCYP71D495  81 TAKRVQSFKSIREEEVSKLVESINFSKMASSLTYAIISRAVCGRRRVEKIHGEVDKIIENIIKAVILDMFIAGSDTSSTT
JcuCYP71D496  81 TAKRVQSFKSIREEEVFKLVESINFSKMASSLTYAIISRAVCGRRRVEKIHGEVDKIIETIIKAVILDMFIAGSDASSTT
RcoCYP71D324  81 TAKRVQSFRSIREEEVSRLMRSLNFSRMFNSLTYSIISRASFGAPRLKRIHHIVDNIFQNIIKGIILDTFVAGSETSSTT
RcoCYP71D326  81 SAKRVQSFRSIREEEVSNLINTLNFSRKLFSSTYGIAARASFGKSRLLRLRDDGDNILENIIKAVILDIFIAGSETSSTT
EpeCYP726A3   81 SPKRVLSFRSIREEQVSNFIKFLNFPKALSELTSRIMLITLLGKSRMEKVLQETDIILEAIIKANLMAMYAGGSETSSKL
EpeCYP726A6   81 SAKRVHSFKPVREERISIFLDSLNLTRTIYGLTNSIIQITAFGKSRMMRAHKEIDVVLDEIIKANMLEMFLSGSHTSSKI
EpeCYP726A5   81 SLKRVQSFRSLREENVSGFIKFLNLTKSVGNLTSSIMLIKTYGKSRMARVQKEMDKMLEQIIKANIMEIFFGGSHTSSKT
ElaCYP726A1   81 SAKKVQSSRLIREEEMEDAITFLNITKIIYGIIISIMIRTSVGESKPRRLHQEIDDILEEIIKASVLQMFTAGSETTSKA
JcuCYP726A25  81 SAKRVQAYKSVMDEEVSNFINFLNLTKTFYSLGNGIIAKTSIGSSTLHTAHQEADEILEEIIKAIILDMFAGASDTSLTT
JcuCYP726A26  81 SSKRVQSFKSVREEEVSSFFKFLNLSRTLLSLTNGIIAKTSIGSSTIRRIHREADTILEEIIKAIILEMFGAASDTSSVT
JcuCYP726A22  81 SAKRVQSFQSVRKEEMADFVNFLNLTHTIFAFTNSIIARNAVGKSRVMKLHYETDKILEDIIKASTIDAFVGGSDTSSKT
JcuCYP726A24  81 STKRVQSFRSVREEEAGEFVKFLNLTHALYALSNSMVARSTVGMSRIWKSHCQTDEILEGIIKGAILEMFGAGSDTSTKT
JcuCYP726A23  81 STKRVQSFRSVREEEVADFVKFLNLTHTLFALTNSIVARTAVGRSRIWKTHRETDKILEDVIKGTIMEMFGAGSDTSSKT
JcuCYP726A21  81 STKRVQSFRSVREEEIAEFVKSLNLSHTLFALTNSIIARNTVGRSRIWKLHCETDEILEGIIKAVILGMFGAGSDTSSKT
JcuCYP726A20  81 STKRVQSFRPVREEEVASFVKLMNLTHALFALTNSIVARNAVGRSRIWKLHQNTDEILEDIIKAAIIDMFGAGSDTSSKT
JcuCYP726A35  81 SVKRVQSFKSVREEEMSSFIKFLNLTHHLFVLTNYIIARTSIGKHKFDKLHRETDKILEDIIKGAILDTFGAGSDTSSKT
EpeCYP726A4   81 SAKRIQSFKSVREQEVESFIRLLNLTTAVFELTNTIMMITTIGKGKLQKLHKETDEILEEIIKAATLEMFGAGSDTSSKT
RcoCYP726A14  81 SAKRVQSFKSVREEELSNFVKFLNLTHTLFALTNNIMARTSVGKSRLEKLHQQADDILEDIIKGAILDMFGAGSDTSSKT
RcoCYP726A17  81 SAKRVQSFRSVREEEVSEFVRFLNLTKTLFALTNSIMARTSIGKSRLERLHRVADQIFEDIIKAAILEMFGAGSDTSSKT
RcoCYP726A13  81 SPKRVQSFSSVRQEELSNYVRFLNLSKTLFALTNSVIAKIAVGKSNLERLHRITDKILEDIVKSAILEMLSGGSDTSAKT
RcoCYP726A16  81 SAKRIQSFSSLRQEELSHFVRFVNLSKVLFALTNSIIARIATGKSRLEKLHRITDQVLEDIIKAAILEMLGGGSDTSAKT
RcoCYP726A12  81 SAKRVQSFKSLREAEVSNFIRFLNLTRKLFALTNTIMARTSVGKSRLERLHQDTDQILEDIIKGTILDMFAGGSDTTSKT
RcoCYP726A10  81 SAKRIQSFKSVREEEVDEFAKFVNLTDKLFALTNTIMARTTIGKFRLEKLHRETDKILEDIIKATILDIFIAGSDTSAKT
RcoCYP726A11  81 SAKRIQSFKSVREEEVSNFISFLNLTDKLFALTNSIMARTSIGKSRLERLHQQTDKILEDIIKAIILTIFIGGSDTSAKT
EpeCYP726A19  81 SIKRVQSFKLVREEEVSNFIKSLNLTEGLFTLTNSIMLRTSIGKSGLQSLRRDADLILDEIIKGAILDMFGAGSDTSSKS
EfiCYP726A36  81 SIKRVQSFKSVREEEVSNFIKLLNLTEKLFALTNSIMLRTSIGKSGLESLRRDADLVLDEIIKATILDMFGAGSDTSSKS
RcoCYP726A15  81 SIKRVQSFKSVREEELSNFIRYLNLTHHLFSLTNSIMFRISIGKSSLEDLHREADYILEDIIKAAILEMFGAGSDTSSKT
RcoCYP726A18  81 SLKRVQSFKSVREDEFSNFIKYLNLTHDLFSLTNSVMLRTSIGRSSLEALRRDTDEILDEIIKATILDMFGAGSDTSSKT
```

```
AthCYP701A3   161 TEWAIYELAKHPQDRLCKEIQNVCGQVPYLNGVFHETLRKYSPAPLTQIGGYHVPAGSEIAINIYGCNMDKKRWKTMAFG
OsaCYP701A6   161 TEWAMYELAKNPQERLYQEIREVCGRLPYLNAVFHETLRRHSPVPLTKLAGYDVPAGTEMVINLYGCNMNRKEWKTMAFG
OsaCYP701A8   161 TEWTMYEIAKNPQELLYQEIREACGRLPYLNAVFHETLRLHSPVPVTTLAGYDIAAGTQMMINVYACHMDEKVWKTMAFG
SmiCYP76AH1   161 IEWAMSELVMNPMARLKAELKSVAGKLPYLQAVIKEVMRIHPPGPLQEVNGYLIPKGTQILINAYAIGRDPSIWELLPFG
CroCYP76B6    161 LEWAMSEMLKNPMKKTQDELAQVIGRLPYLRCVMKETLRIHPPVPFVEVCGYNVPKGSQVLVNAWAIGRDETVWELIPFG
OsaCYP76M7    161 LEWVMAELLKNPMAKARAELRDVLGRLPYLQAVLKEAMRLHPVGALVEVGGYAVPKGSTVLFNAWAIMRDPAAWEFMPFG
OsaCYP76M8    161 LEWVMAELLKNPMAKARAELRDVLGRLPYLQAVLKEAMRLHPVGALVEVGGYAVPKGSTVLFNAWAIMRDAAAWEFMPFG
SbiCYP71E1    161 ILWAMSELMRKPLRKAQAEVRAAVGKIPYLKMVVKETLRLHPPATLTTICGYDVPANTRVFVNAWAIGRDPASWELIPFG
MesCYP71E7    161 ITWAFSELLKNPMKKAQEEVRRAVGKIKYIDCIVKETFRKHPPVPLCKIGGYDILPGTTIYVNAWAMGKDPTIWELVPFG
OsaCYP71Z6    161 LIWTLAELMRNPMAKAQAEVRQAVAGLSYLKMVIKETLRLHPPAPLSQVMGYDIPKGTSVFVNMWAICRDSRYWEFLPFG
OsaCYP71Z7    161 LIWILAELMRCPMAKAQAEVRQAAVGLSYLKMVIKEALRLHSPAPLTQVMGYDIPKGTSVFVNMWAICRDPNYWEFLPFG
ZzeCYP71BA1   161 IDWVMSELIKNPMEKVQKEMREAMQKFSYLNLVIKETLRLHPPGPLCEVMGYRVPAGARLLINAFALSRDEKYWEFMPFG
OsaCYPCYP99A3 161 TEWLMSELMRNPMAKVQAEVRGVFDNLSYMKLVIKETLRLNPVLPLCEIGGYEIVEGTRVLINSWAMARSPEYWEYLPFG
CciCYP71BL3   161 LEWAMTELMRNPMKRAQDEVRSVVKSLHYLKLIVKETLRLHAPTPLCNVDGYDIPAKTKILVNAWACGTDPDSWEFIPFG
LsaCYP71BL2   161 LEWAMTELMRNPMKRAQEEVRSVVKSLHYLKLIVKETLRLHAPTPLCNVDGYDIPAKTKILVNAWACGTDPDSWEFIPFG
MgrCYP71D95   161 TLWVMAELMRNPMAKAQAEVRAALKELKYMKSVVKETMRMHPPIPLCEVNGYKIPNKARIMINVWSMGRNPLYWEFIPFG
MgrCYP71D18   161 ISWALSELMRNPMAKVQAEVREALKELKYLRSVLKETLRLHPPFPLCEVNGYTIPAKTRIFINVWAIGRDPQYWEFIPFG
RcoCYP71D329  161 IEWAMSELMRNPLKKAQAEVREAFKDLKYLELVIKETLRLHPPLPLCAIGGYQIPAKTRMIVNAYAIGRDPKTWEYIPFG
CroCYP71D12   161 VDWAMCEMIKNPMKKAQEEVRKVFNQLKYLQAVIKETLRLHPPVPLCKIKGYTIPSKSRVIVNAWAIGRDPNYWEYLPFG
AanCYP71AV1   161 IEWAISELIKCPMEKVQAELRKALNELSYLNMVIKETLRLHPPLPLVNLAGYNIPNKTKLIVNVFAINRDPEYWEYLPFG
CinCYP71AV8   161 IEWAISELIRCPMEKVQTELRQALNELNYLKLVIKETLRLHPPLPLCVLGGYDIPSKTKLIVNVFAINRDPEYWEYLPFG
BspGAO        161 IEWAISELIRCPMEKVQTELRQALNELSYLKLVIKETLRLHPPLPLCVLAGYEIPTKTKLIVNVFAINRDPEYWEYLPFG
NtaCYP71D16   161 INWAMVEMMKNPFSKAQAEVREILREFKYLKMVIKETFRLHPPLPLIDLNGYTIPLKTKVVVNAWAMGRDPKYWEYLPFG
HmuCYP71D55   161 LVWAMVQMMRNPLAKAQAEVREAFKELKYLKLVIKETLRLHPPVPLTEINGYTIPVKTKVMVNVWALGRDPKYWEYLPFG
NtaCYP71D20   161 TVWAMAEMMKNPFTKAQAEVREAFRELKYLKLVIKETLRLHPPSPLTDINGYTIPAKTKVMVNVWALGRDPKYWEFLPFG
RcoCYP71D335  161 IEWTMSELIKNQMEKAQAEVRQIFGQLIYLKLVINETLRLHPPAPMCVINGYDIPTMSKVIINAWAIGRDPRYWEFLPFG
RcoCYP71D336  161 IEWTMSELIKNPMEKAQAEVREMFGNLNYLKLVIYETMRLHPPAPLCVINGYDIPAKSNVILNLWALGRDPRYWEYLPFG
JcuCYP71D495  161 IEWAISEMVKNPMEKAQAEVREVFGELNYLKLVIKETLRLHPAVPLCVIEGYNIATKSTVIVNAWAIARDPKYWEFIPFG
JcuCYP71D496  161 IEWAMSEMVKNPMEKAQAEVRKVFGELNYLKLVIKETLRLHPALPLCVIEGYNIATKSTVIVNAWAIARDPKYWEFTPFG
RcoCYP71D324  161 AEWAMSELLKNPMEKAQEEVRRVFGELNYLKWVINETLRLHPPIPLCVINGYDIPVKSKVIVNVWAIGRDPNCWEFLPFG
RcoCYP71D326  161 VEWAMSEMLRNPMEKAQEEVRRVFGELNYLKLVIKETLRLHPPAPLCEINGYYIPVNSKVIVNAWAIGRDPNNWEFIPFG
EpeCYP726A3   161 IEWTFAEMMKNPMRKAQEEVRRVFGELKYLKLVLKESFRIHPPSTLTKINGYDIHPKTTILINVWTMGRDPNLWELTPFG
EpeCYP726A6   161 TEWTMAELMRAPMRKAQEEVRRVFSECKYVKNVLKEAFRLHPPGPMTKVNGYEILPGTTVFINVWAIGRDPEVWELIPFG
EpeCYP726A5   161 VEWAMSELMKNPMTKAQAEVRQVFGEVKYLKSVIKETLRLHPPATFTKVNGYDIYPKTVVHVNTYAICRDPDVWELIPFG
ElaCYP726A1   161 TEWVMAELMKNPLRKAQEEVRQVFGDLKFFKLVVKETLRLHPPVVLTRIDGYEIHPNTRIVVNAWAIGRDPNTWELVPFG
JcuCYP726A25  161 AEWAMAEMVKHPMKKAQDEVRRTLNELKYLKLVIKETLRLHPPVALCELNGYDVNPKTKILVNAWAIGRDHNLWKFIPFG
JcuCYP726A26  161 IEWAMSEMMKNPMKKAQEEVREVFNELQYLKLVIKETLRLHPPLTLCQINEYDIYPKTRVLVNAWAIGRDPNWWEFIPFG
JcuCYP726A22  161 TEWAMAELMRKPMKKAQEELRSVFGELKWLKLIIKETMRLHPVLSLTKVSGYDVYPGTQVLVNVWALGRDPKHWEYLPFG
JcuCYP726A24  161 LEWAMSELMRNPMKKVQQELRSFFGELKWLKLIIKETLRLHPPIAVTKVCGYDVYPNTRVFVNVWAMGRDPKIWELIPFG
JcuCYP726A23  161 VEWAMAELMRNPMRKAQEELRSFFGELKCLKLIIKETLRLHPPGAVTKVAGYDIYPNTKIFVNTWAIGRDPEIWELIPFG
JcuCYP726A21  161 TEWAMAELMKNPMKKAQEELRSLFGEIKWLKLIINETLRLHPAVTLTKVSGYDVYPNTRVFINTWAIGRDPTIWEYTPFG
JcuCYP726A20  161 AEWAMAELMRNPMKKAQEELRNFFGELKWIKLIIKETLRLHPAVAVTKVYGYDVEPGTRVFINVWSIGRDPKVWELIPFG
JcuCYP726A35  161 AEWTLSELIRNPMRKAQAEIRRVFDELKYLKLVVKETLRLHPAVPLTKINGYDIFPKTKVLVNVWAISRDPAIWELTPFG
EpeCYP726A4   161 TDWAMAQLMRKPMKKAQEEVRRVFSELKYLKLIVKETLRLHPAVALTKIEGFDVYPKTKILVNPWAIGRDPKVWELIPFG
RcoCYP726A14  161 AEWALSELMRHPMKKAQEEVRRIFGELKFLNLVIKETLRLHPPVALTKVNGYDIYPKTRTLINVWSMGRDPSVWELIPFG
RcoCYP726A17  161 TEWAMSELMRNPMRKAQEEVRRVFGELKFLKLVVKETXRLHPAIALTKVDGYDIKPTARVLVNVWAIGRDPNVWELLPFG
RcoCYP726A13  161 IEWAMSELMRSPMEKAQEEVRRVFGELKYLKLVIKETLRLHPALALTKIDGYDISPKTKALVNVWAIGRDPSVWELLPFG
RcoCYP726A16  161 TEWAMSEMMRYPMKKAQEEVRQAFGELKYLRAVFKETLRLHPPLAMTKINGYDIYPKTKTLINVYAIGRDPNVWELIPFG
RcoCYP726A12  161 AEWAVSELMFNPMKKAQEEVRRVFGELKFLKLVIKETLRLHPALPLSKINGYNIDPKTKVLINVWAIGRDSNIWEFIPFG
RcoCYP726A10  161 VEWAMSELMRNPMKRAQEEVRQVFGDLKFMKLIVKETLRLHPVFAMTKVNGYDISPKTTMLINVWAIGRDPNVWEMIPFG
RcoCYP726A11  161 VEWVMSELMHNPMKKAQEEVRQVFGELKFLKSVVKETLRLHPVFPLTKVNGYDIYPKTKVLINVWAIGRDPDIWEMIPFG
EpeCYP726A19  161 TEWALSELLRHPMKKAQDEVRRVFAQLKYLKLVIKETLRLHPAVPLTKVNGYDILPKTKALVNIWAISRDPKIWEFAPFG
EfiCYP726A36  161 TEWALSELLRHPMKKAQDEVRKVFSQLKYLKLVIKETLRLHPAIPLTKVNGYDILPKTKALVNIWAISRDPTIWEFAPFG
RcoCYP726A15  161 AEWALSELMRHPMEKAQTEVRQVFGELKFLKLVIKETLRLHPPVALTKVNGYDIDPKTKVLVNVWAISRDPNIWEFAPFG
RcoCYP726A18  161 AEWALSELMRHPMKKAQEEVRGVFGELKYLKLVIKETLRLHPAIPLTKINGYDVYPKTKVLVNIWAISRDPNIWEFAPFG
```

```
AthCYP701A3   241 AGKRVCAGALQASLMAGIAIGRLVQEFEWENVDTYGLTSQKLYPL
OsaCYP701A6   241 AGRRACAGSLQATHIACAAVARFVQEFGWEKVDTVQLTAYKLHPL
OsaCYP701A8   241 AGRRTCAGSLQAMNIACVAVARLVQELEWDKEDTMQFTALKLDPL
SmiCYP76AH1   241 SGRRVCPGMPLATRILHMATATLVHNFDWHAGELFGVAVRRAVPL
CroCYP76B6    241 AGRRICPGLPLALRTVPLMLGSLLNSFNWDMEEKFGITLQKAHPL
OsaCYP76M7    241 SGRRLCPGLPLAERVMPFILASMLHTFEWDVSEKFKSANVLAVPL
OsaCYP76M8    241 SGRRLCPGLPLAERVVPFILASMLHTFEWDVSEKFKTANVLAVPL
SbiCYP71E1    241 AGRRICPGLTMGETNVTFTLANLLYCYDWSMEETGALTFHRKTPL
MesCYP71E7    241 AGRRICPGLAMGTTAVKYILSNLLYGWDYPLIEEGGLTVHNKQDI
OsaCYP71Z6    241 SGRRICPGINLGVANLELPLASLLYHFDWDMHETSGMVAAKLITL
OsaCYP71Z7    241 SGRRICPGINLGLANLELALASLLYHFDWDMQETPGIVAAKLTTL
ZzeCYP71BA1   241 AGRRICPGMTFGISSVEVALAHLLFHFDWDMMEVSGMSATRRSPL
OsaCYP99A3    241 TGRRRCPGDIFAMATLELIVARLLYYFDWDMELVVGATARRKNHL
CciCYP71BL3   241 AGRRICPGLTFGLSMVEYPLANFLYHFDWDITEITGISTSLKHQL
LsaCYP71BL2   241 AGRRICPGLTFGLSMVEYPLANFLYHFDWDITEITGISTSLKHQL
MgrCYP71D95   241 AGRRICPGLNFGLANVEVPLAQLLYHFDWDMSEAEGLTGIRKNNL
MgrCYP71D18   241 AGRRICPGLHFGLANVEIPLAQLLYHFDWDMTETPGLSGPKKKNV
RcoCYP71D329  241 GGRRICPGMNLGMANMQLPLAQLLYHFDWDMTETFGATITRKNKL
CroCYP71D12   241 GGRRICPGITFALANIELPLAQLLFHFDWNMKESRGVTVRREDDL
AanCYP71AV1   241 AGRRMCPGAALGLANVQLPLANILYHFNWDMTESSGATMQRKTEL
CinCYP71AV8   241 AGRRMCPGAALGLANVELPLAHILYYFNWDMTESFGATVQRKTEL
BspGAO        241 AGRRMCPGAALGLANVELPLAHILYYFNWDMSECFGATVQRKSEL
NtaCYP71D1    241 SGRRICPGISFGLANVYFPLAQLLNHFDWDLTEAAGAACARKNDL
HmuCYP71D55   241 GGRRICPGISFGLANVYLPLAQLLYHFDWDLTELVGVTAARKSDL
NtaCYP71D20   241 GGRRICPGMSFGLANLYLPLAQLLYHFDWDLTELSGITIARKGGL
RcoCYP71D335  241 GGRRMCPGISFGMATVELPLARMLYHFDWDMTEYLGVAGRRKNDL
RcoCYP71D336  241 GGRRICPGNLFAMAIVELPLAQLLYHFNWDMTDQQSLAGCRKNRL
JcuCYP71D495  241 AGRRMCPGMLFGLASVELPLAQLLYHFDWDMSDDLDGTATRRHAL
JcuCYP71D496  241 AGRRRCPGMLFGLASVELPLAQLLYHFDWDMSDNPGGTATRRHAL
RcoCYP71D324  241 AGRRMCPGILFGIINVEFPLAQLLYHFDWDMTEDFGAVVKRKSDL
RcoCYP71D326  241 AGRRMCPGILFGMANVELPLAQFLYHFDWDMMEGFGATVRRKNDL
EpeCYP726A3   241 VGKRMCPGITLSTTYVEFLLANLLYHFDWDMTETLRGTLKKVQDL
EpeCYP726A6   241 AGKRICPGLTLAVVYVELLLANLLYHFDWDMTEFFRGTLNRKEDL
EpeCYP726A5   241 AGKRICPGISLATTYVEVLLANLLYHFDWDMTEMHCGALARKHDL
ElaCYP726A1   241 AGKRICPGITSAITNLEYVIINLLYHFNWDMTEAIGGALRKKIDL
JcuCYP726A25  241 AGKRICPGITMAITIIEVLLAQLLYHFDWDMSDTFGLVVKRRIDL
JcuCYP726A26  241 AGKRMCPGITMAMANIELILAQLLYHFNWDMSESFGLAVKRKVEL
JcuCYP726A22  241 AGKRVCPGIALGMVHMENFLANLLFHFDWDMTDAFGGVMKRKVDL
JcuCYP726A24  241 AGKRICPGITLAIVHVETVLANLLYHFDWDMNETFAGIIRRKVDL
JcuCYP726A23  241 AGRRICPGITLASANMELFLANLLYHFDWDMNECFGGAVKRKVDL
JcuCYP726A21  241 AGRRICPGMAFGMVNLEIFLANLLYHFDWDMTENFGGVIKRKQDL
JcuCYP726A20  241 AGKRICPGMTLGMANLEIFLANLLYHFDWDMNEAFGGAVKRKVDL
JcuCYP726A35  241 AGKRICPGMTLGITNLELFLANLLYHFDWDMSESFGGAIKRKTDL
EpeCYP726A4   241 AGKRICPGMTLGIVNLELFLANLLYHFDWDMTEAIGGAIKRKLDL
RcoCYP726A14  241 AGKRICPGMTLGIVNLELFLAHLLYHFDWDMSEGFGGALKRKMDL
RcoCYP726A17  241 AGKRICPGILVGITNLELVLAHLLYHFDWDMREGFGGALHRKSDL
RcoCYP726A13  241 SGKRICPGMTLGLATVELFLSYLLYYFDWDMTEAFAASLKRKIDL
RcoCYP726A16  241 AGKRICPGMTLAITTVELFLAHLLYYFDWDMTESFGASIKRKIDL
RcoCYP726A12  241 AGKRICPGMMLGTTNLELFLAQLLYHFDWDMTEAFSGSINRKYDL
RcoCYP726A10  241 AGKRICLGMTLGTLILEHFLAKLLYHFDWDMTEHYSASMRRETDL
RcoCYP726A11  241 AGKRVCPGMSLGLLILELFLAQLLYHFDWDMSEYYSSSLRRKHDL
EpeCYP726A19  241 SGKRICPGMALGITNLELFLAQLLYHFDWDMGEVVGGAIKRKVDL
EfiCYP726A36  241 SGKRICPGMALGITNLELFLAQLLYHFDWDMSEVVGGAIKRKIDL
RcoCYP726A15  241 SGKRICPGMNLGLTNLELFLAQLLYHFNWDMTESVGAAIKRKIDL
RcoCYP726A18  241 SGKRVCPGMTLGITNLELILAKLLYHFDWDMTESVGGAIKRRTDL
```

Supporting Figure S8 – NMR data for 6-hydroxy-5-keto-casbene.

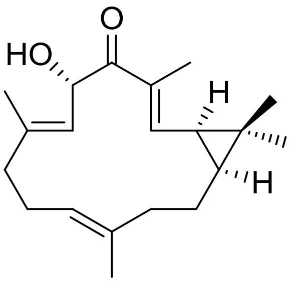

6-hydroxy-5-keto-casbene **(2)**

^{1}H NMR (700 MHz)

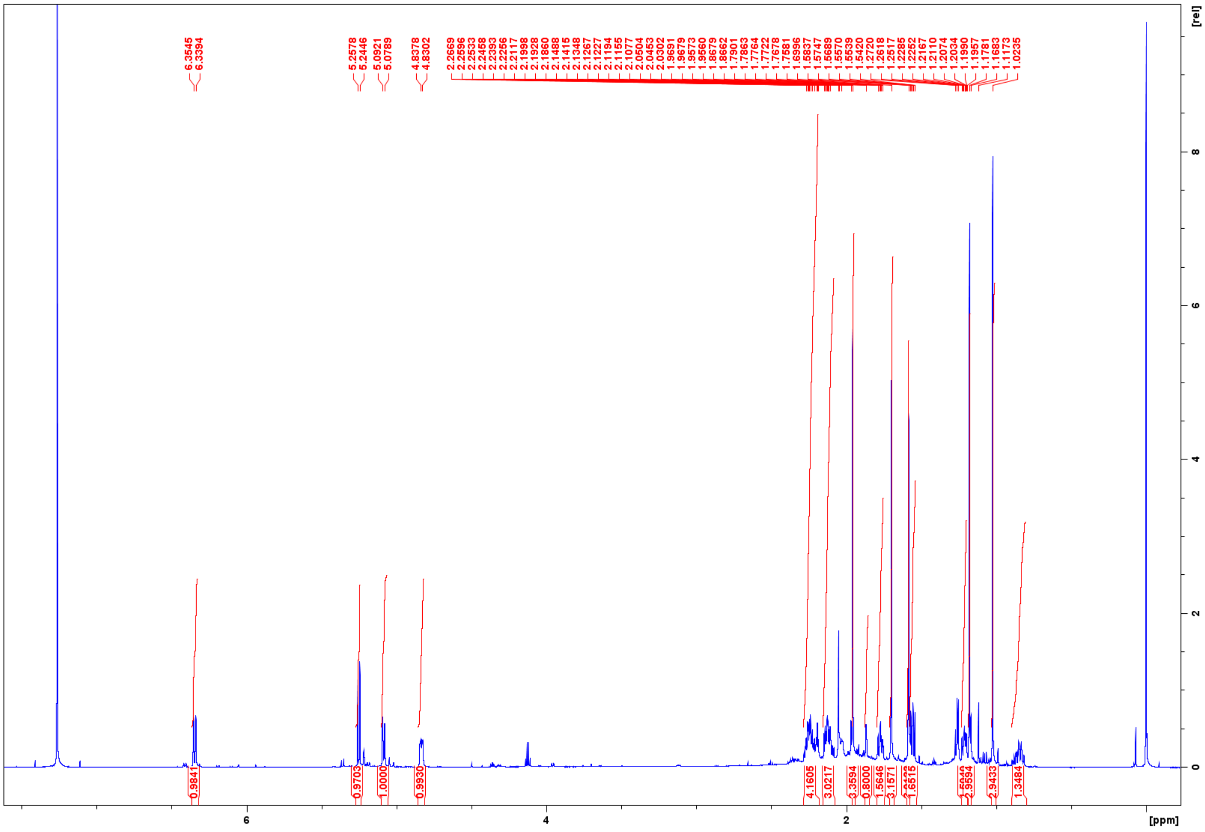

Supporting Figure S8 continued – NMR data for 6-hydroxy-5-keto-casbene.

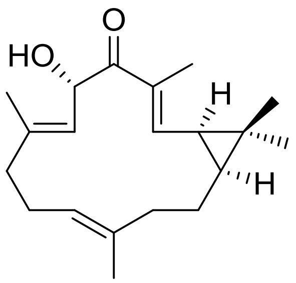

6-hydroxy-5-keto-casbene **(2)**

^{13}C NMR (175 MHz)

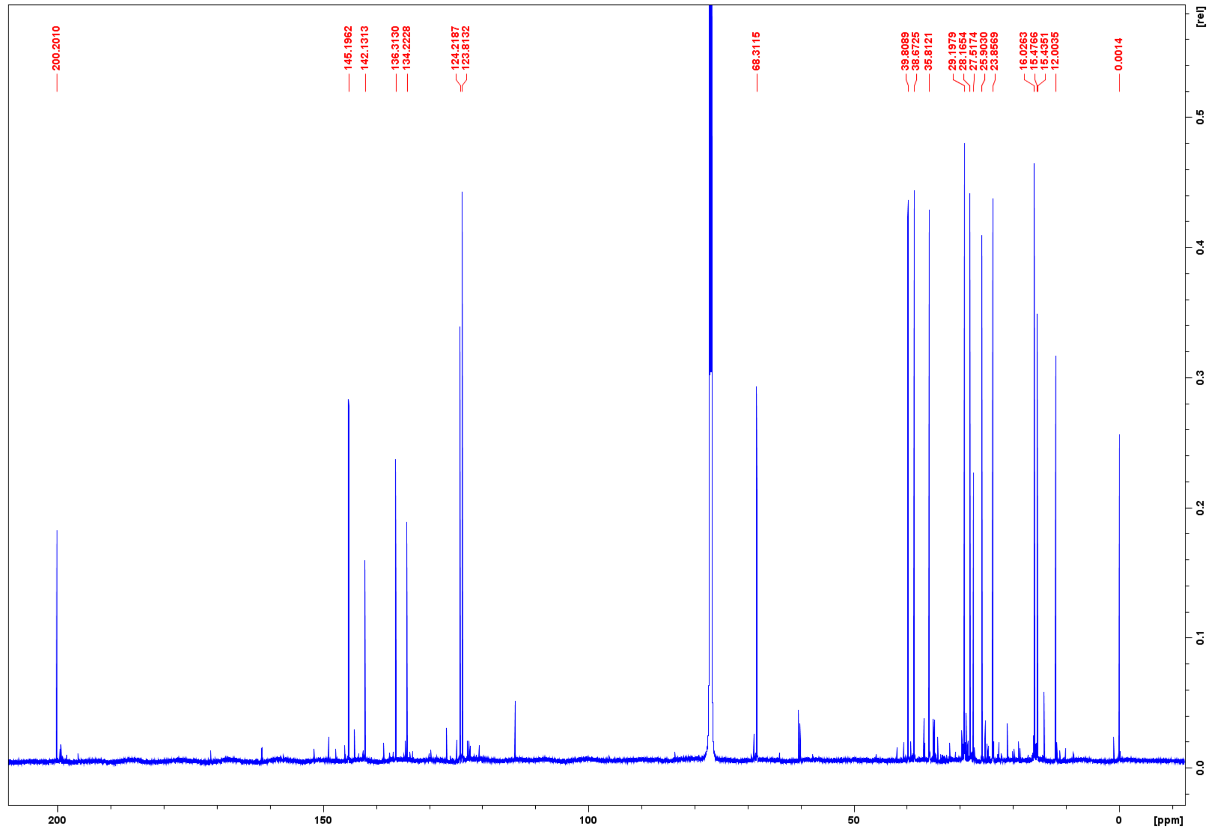

Supporting Figure S8 continued – NMR data for 6-hydroxy-5-keto-casbene.

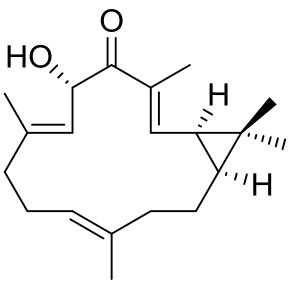

6-hydroxy-5-keto-casbene **(2)**

Edited-HSQC

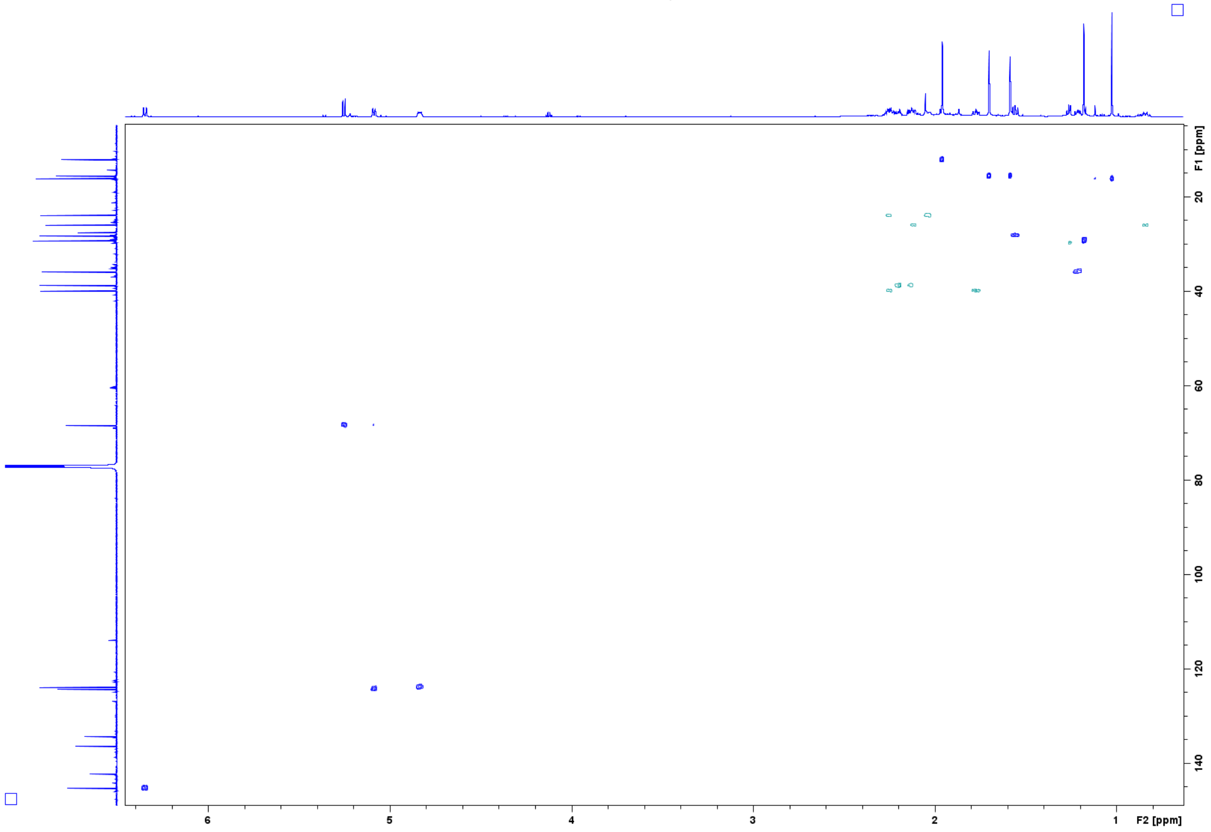

Supporting Figure S8 continued – NMR data for 6-hydroxy-5-keto-casbene.

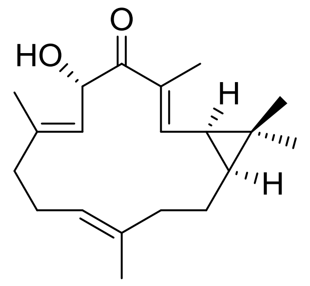

6-hydroxy-5-keto-casbene **(2)**

HMBC

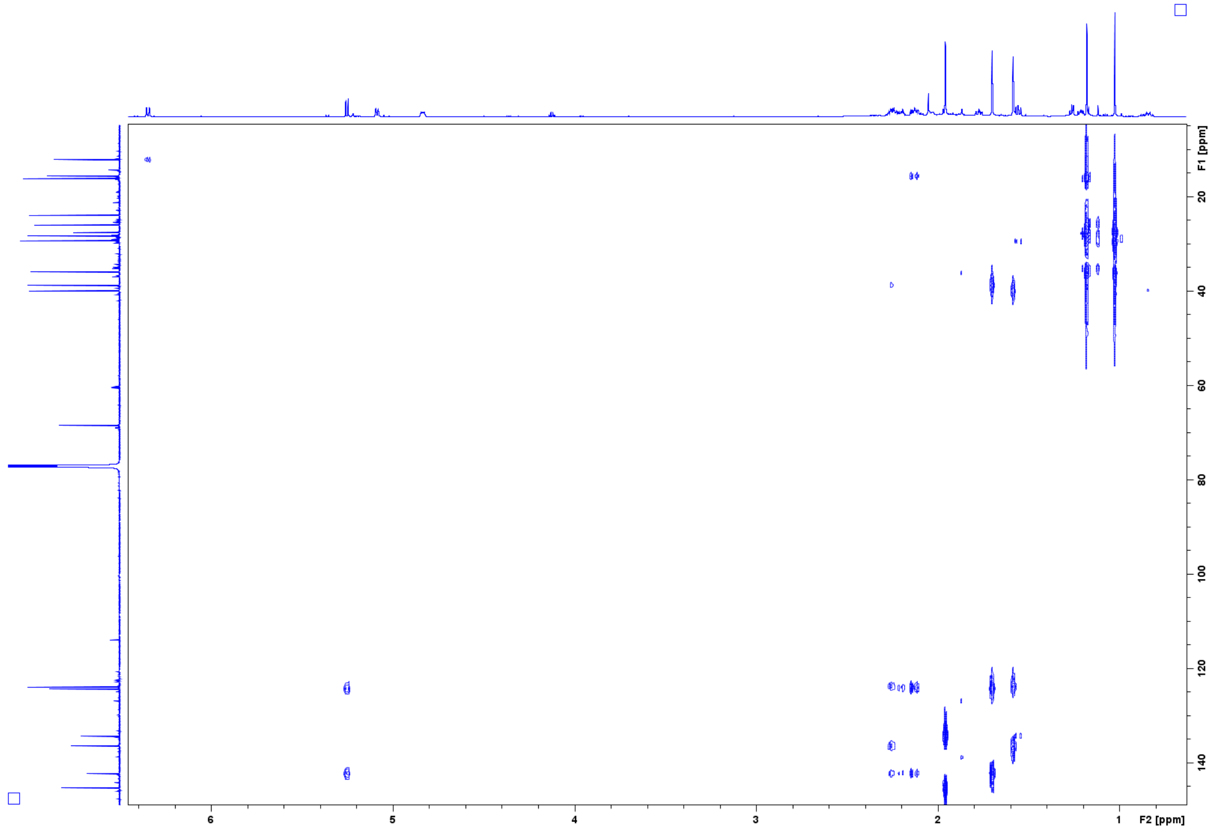

Supporting Figure S8 continued – NMR data for 6-hydroxy-5-keto-casbene.

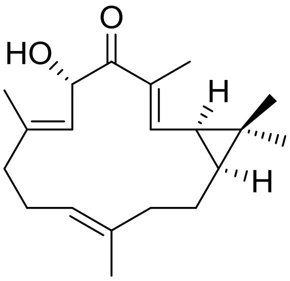

6-hydroxy-5-keto-casbene **(2)**

^{1}H-^{1}H COSY

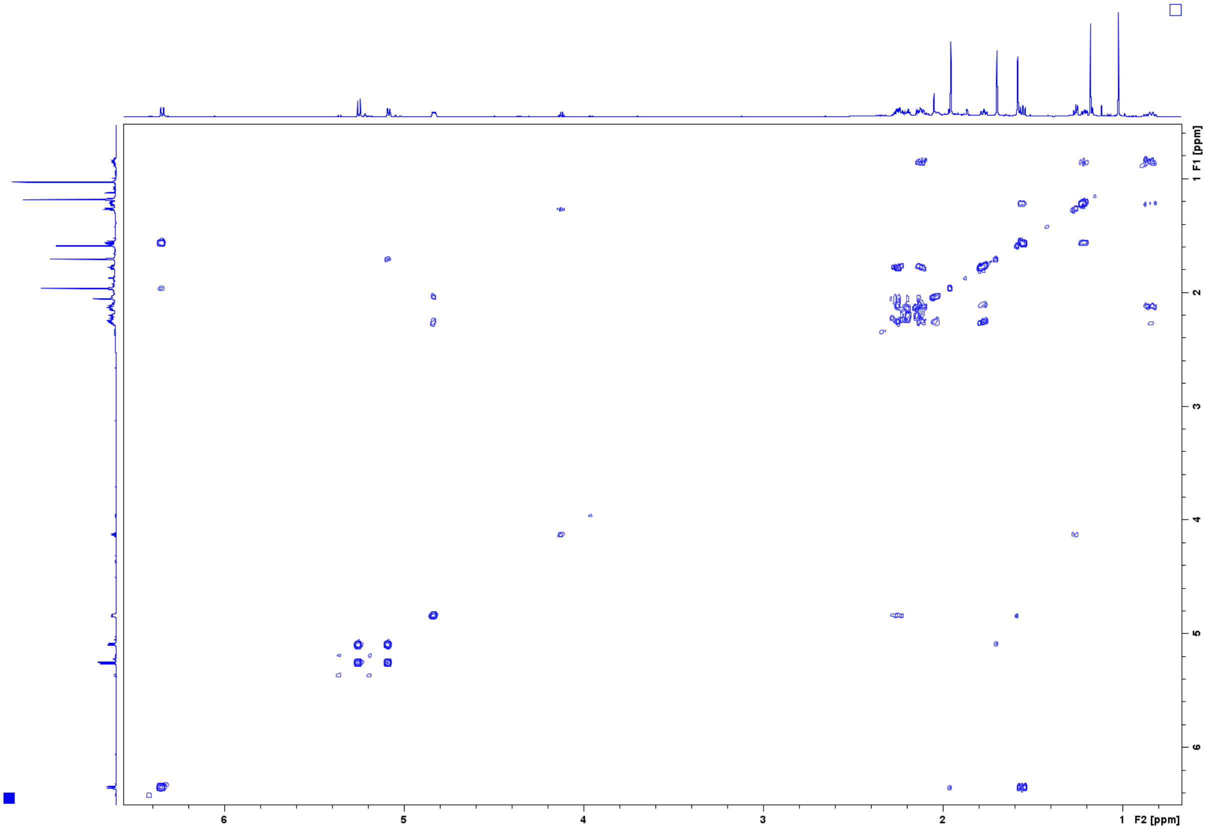

Supporting Figure S8 continued – NMR data for 6-hydroxy-5-keto-casbene.

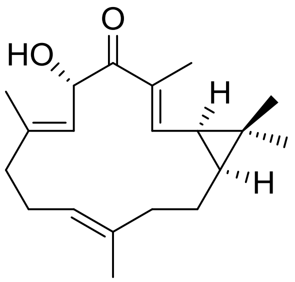

6-hydroxy-5-keto-casbene **(2)**

NOESY

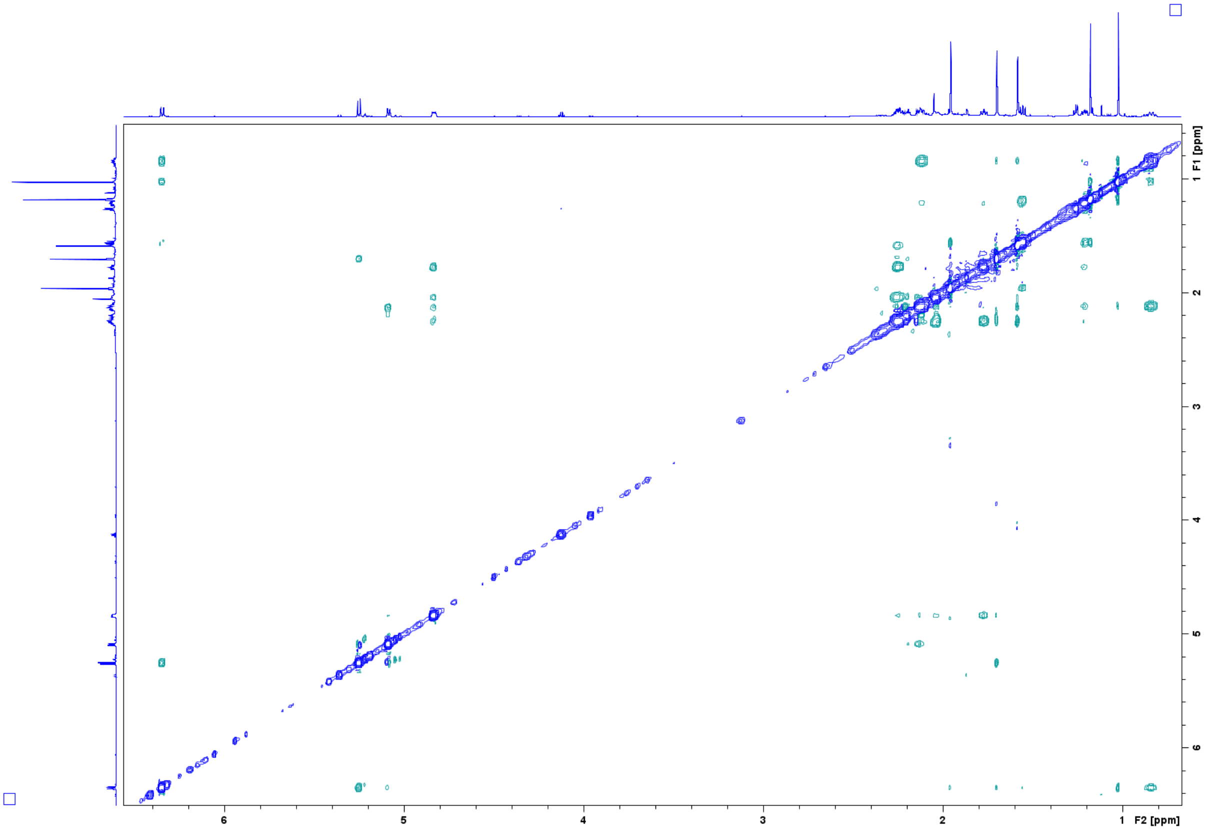

Supporting Figure S9 – NMR data for 9-keto-casbene.

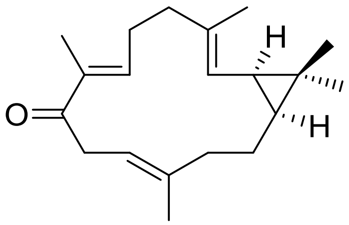

9-keto-casbene **(3)**

^{1}H NMR (750 MHz)

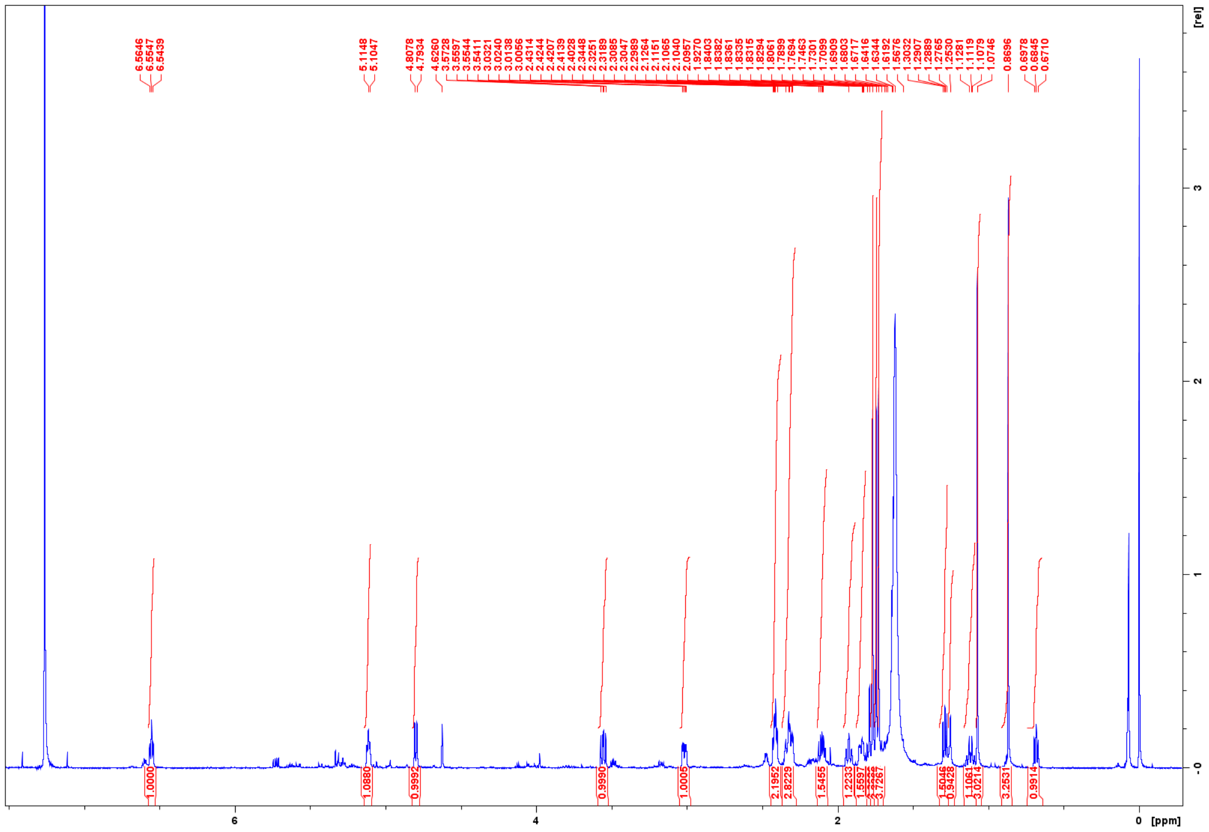

Supporting Figure S9 continued – NMR data for 9-keto-casbene

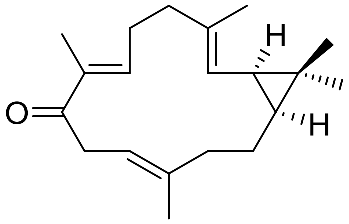

9-keto-casbene **(3)**

^{13}C NMR (175 MHz)

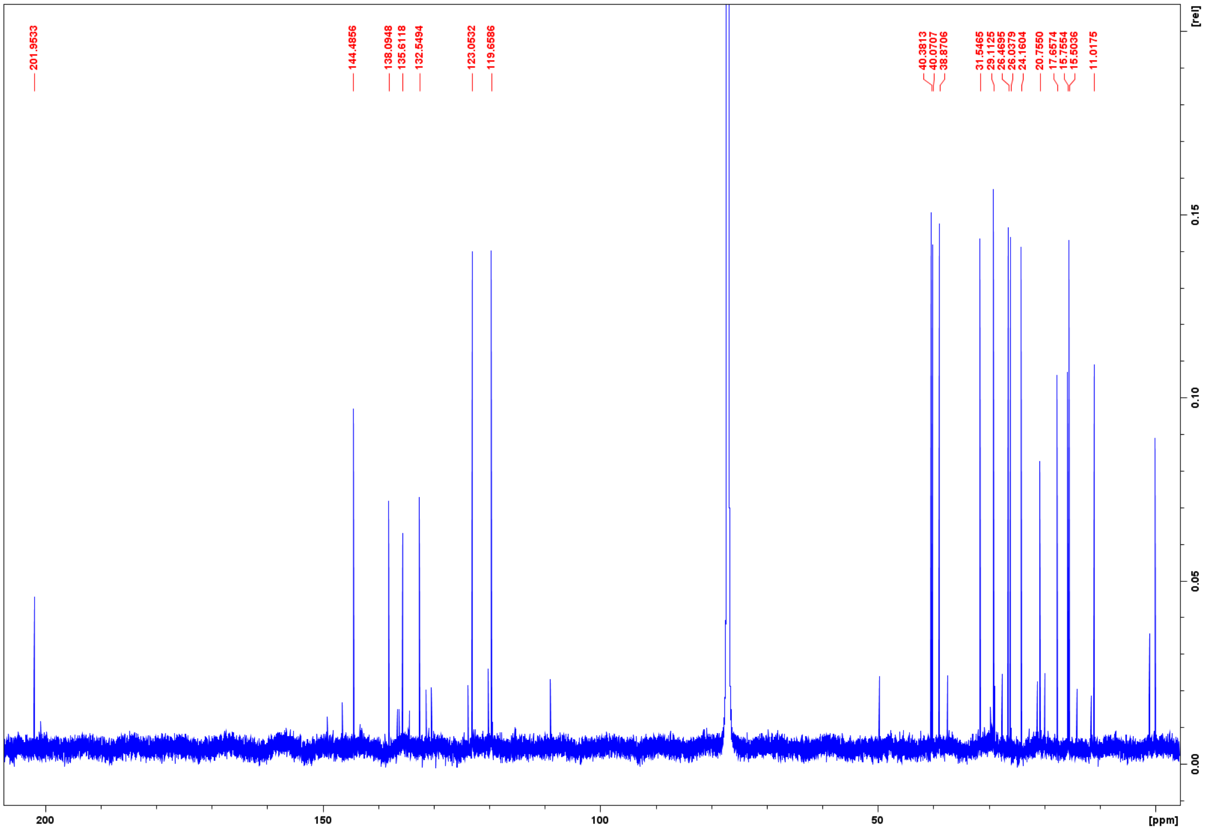

Supporting Figure S9 continued – NMR data for 9-keto-casbene

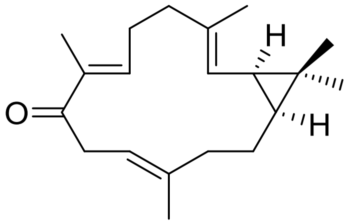

9-keto-casbene **(3)**

Edited-HSQC

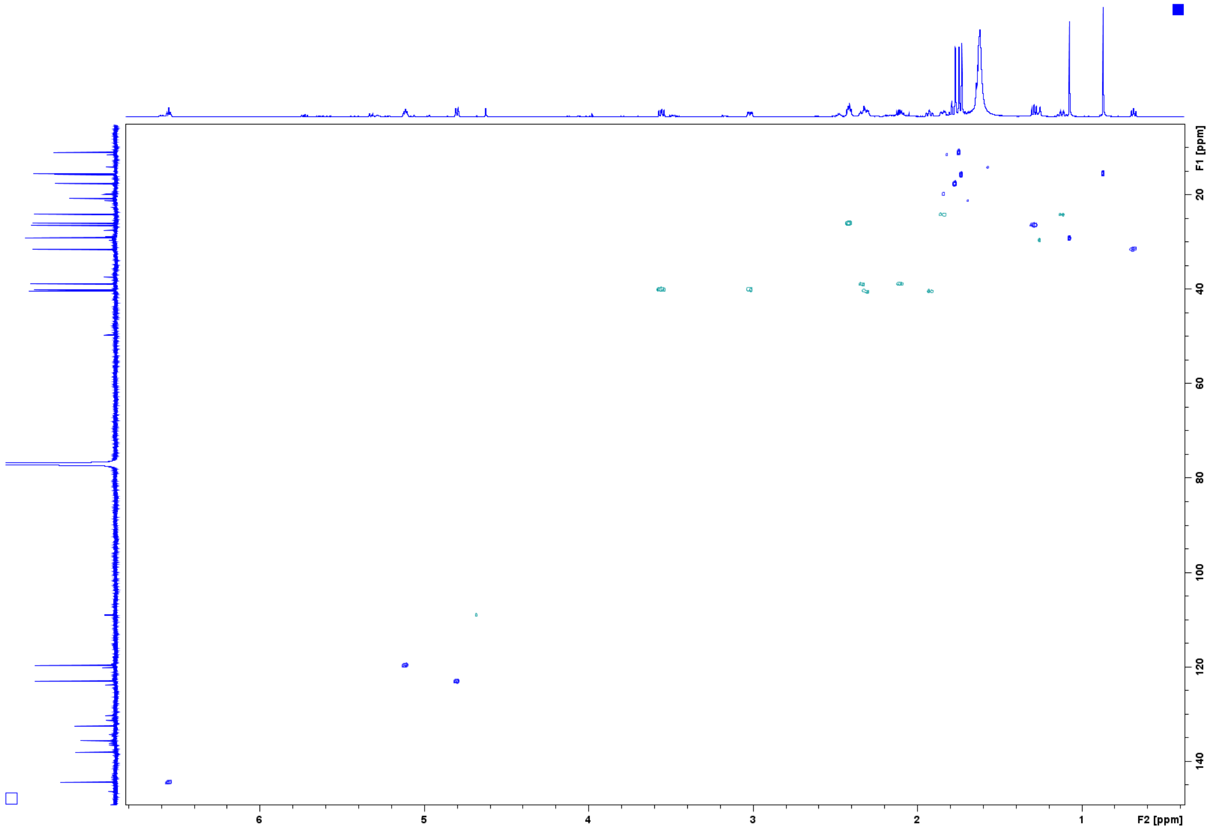

Supporting Figure S9 continued – NMR data for 9-keto-casbene

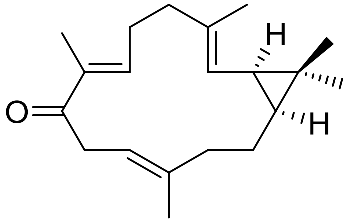

9-keto-casbene **(3)**

HMBC

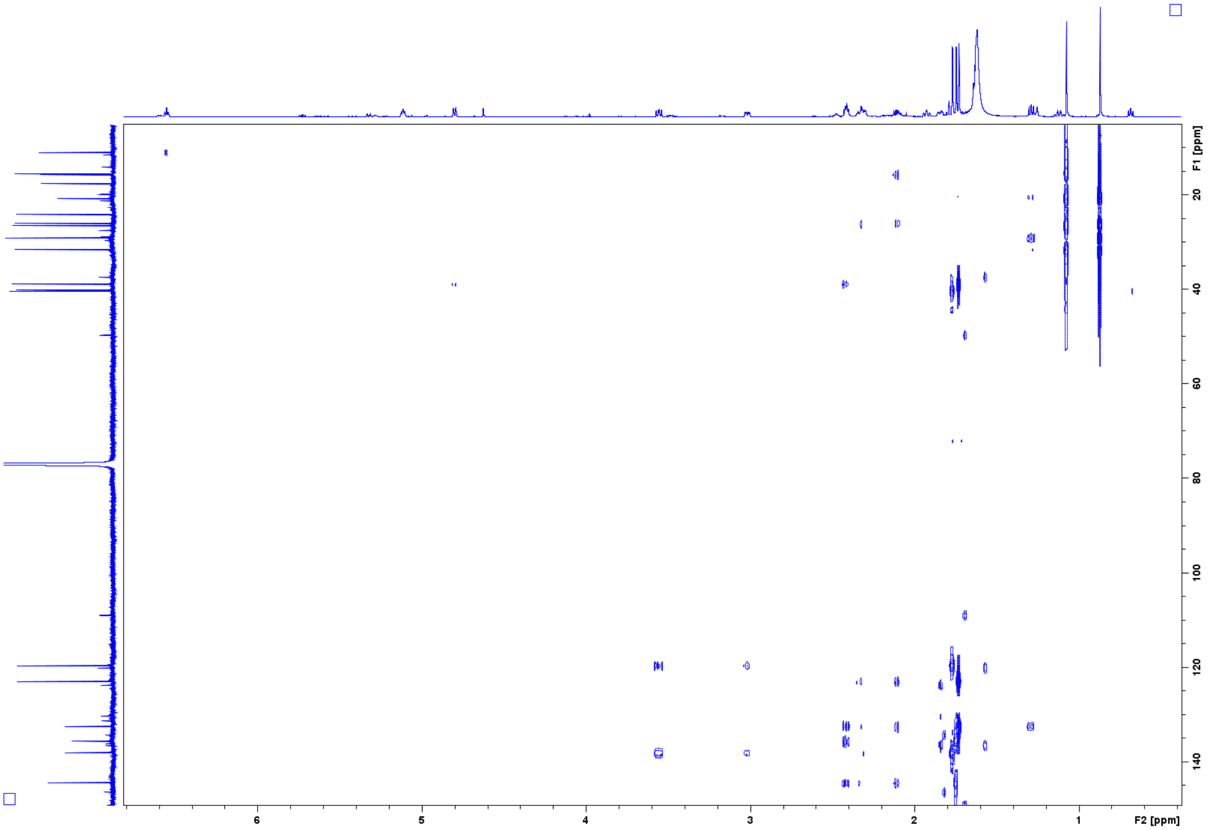

Supporting Figure S9 continued – NMR data for 9-keto-casbene

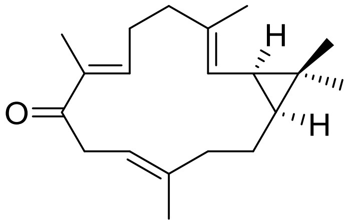

9-keto-casbene **(3)**

^{1}H-^{1}H COSY

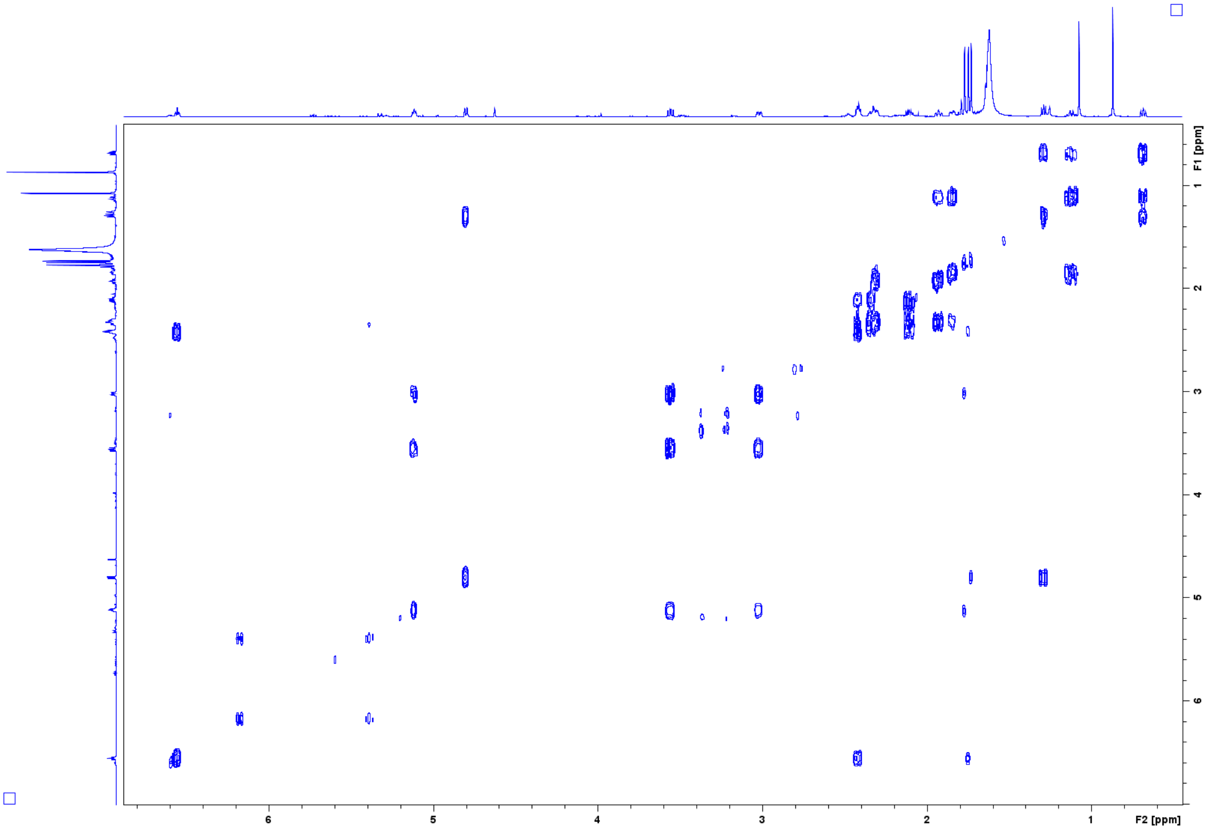

Supporting Figure S9 continued – NMR data for 9-keto-casbene

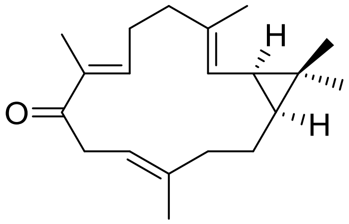

9-keto-casbene **(3)**

NOESY

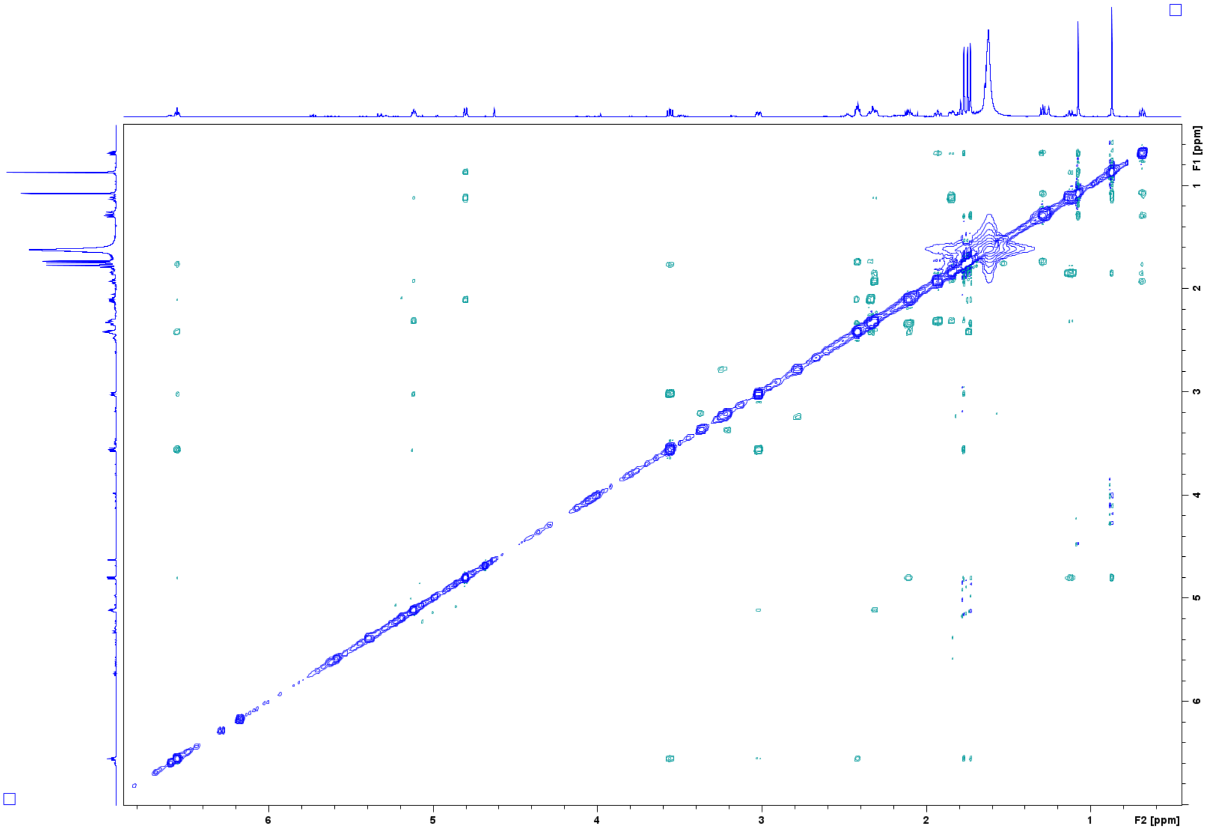

Supporting Figure S10 – NMR data for 9-hydroxy-5-keto-casbene

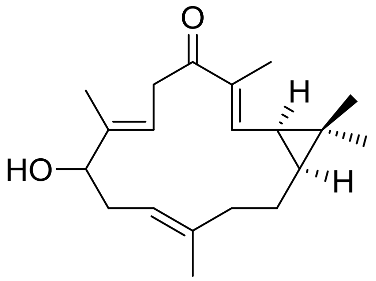

9-hydroxy-5-keto-casbene **(6)**

<u>^{1}H NMR (700 MHz)</u>

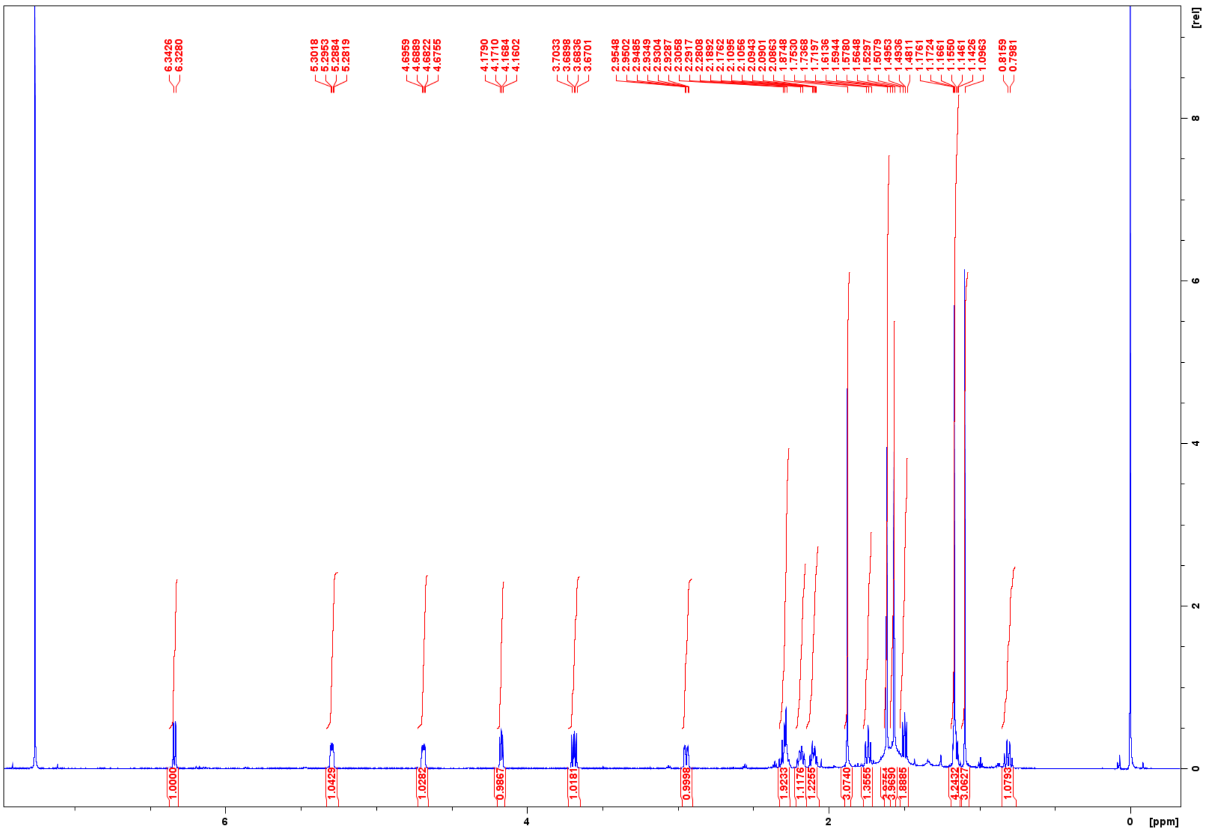

Supporting Figure S10 continued – NMR data for 9-hydroxy-5-keto-casbene

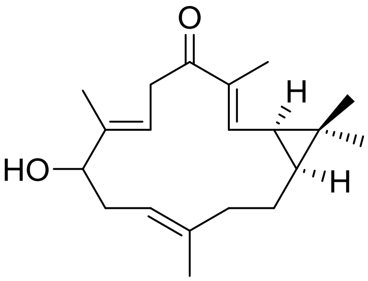

9-hydroxy-5-keto-casbene **(6)**

^{13}C NMR (175 MHz)

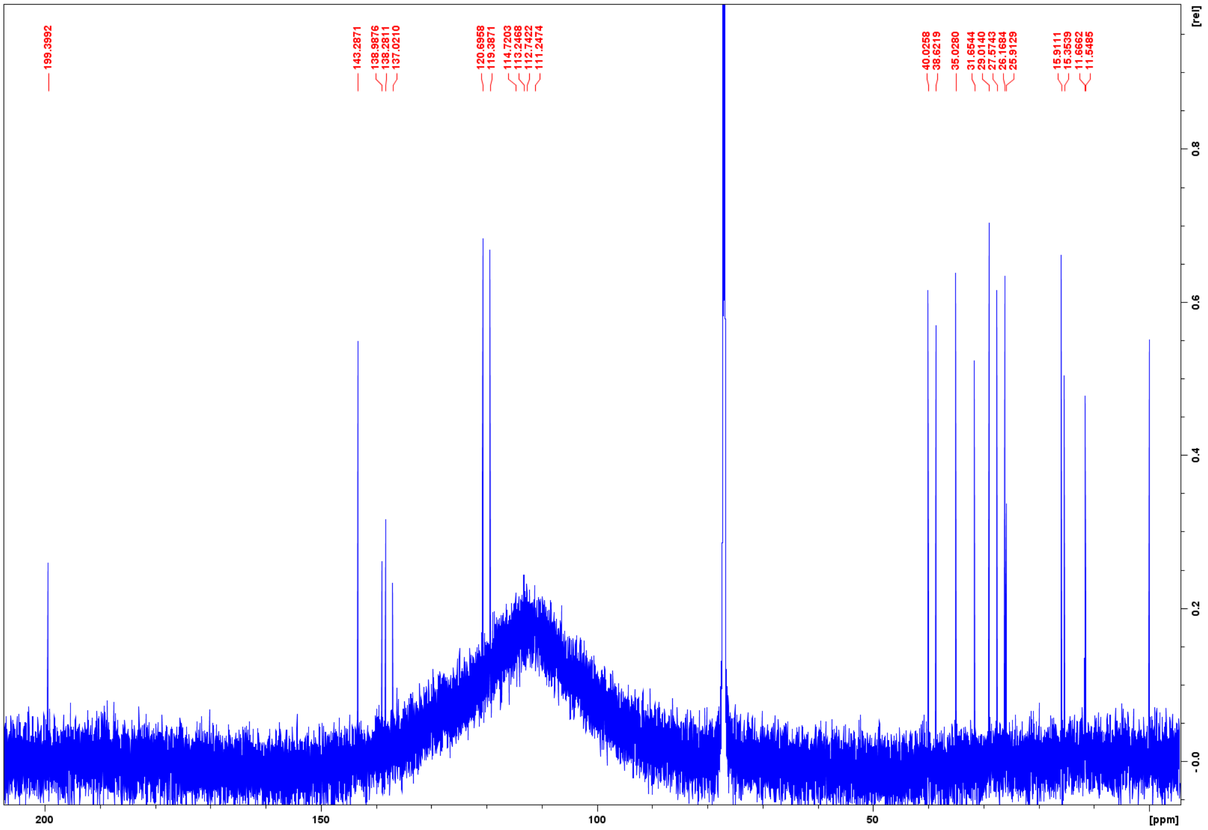

Supporting Figure S10 continued – NMR data for 9-hydroxy-5-keto-casbene

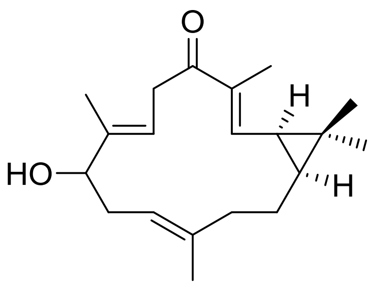

9-hydroxy-5-keto-casbene **(6)**

<u>Edited-HSQC</u>

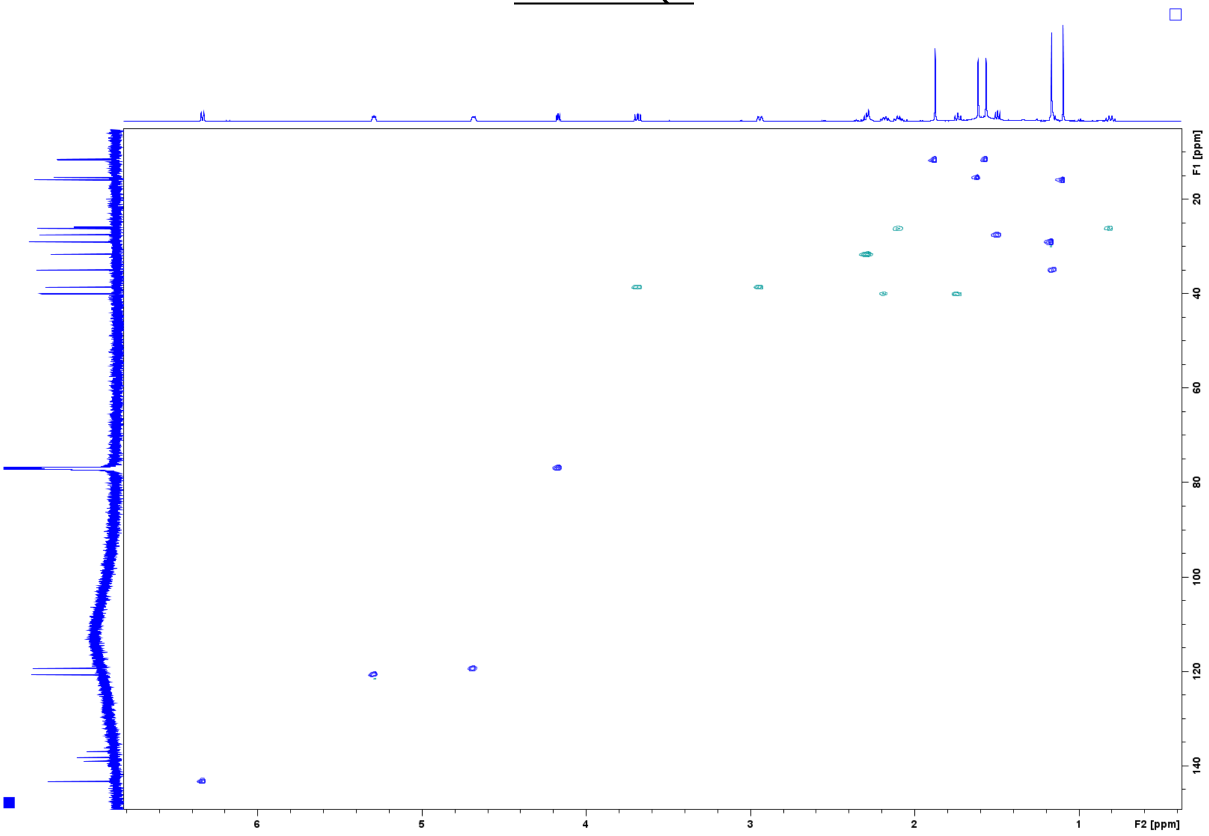

Supporting Figure S10 continued – NMR data for 9-hydroxy-5-keto-casbene

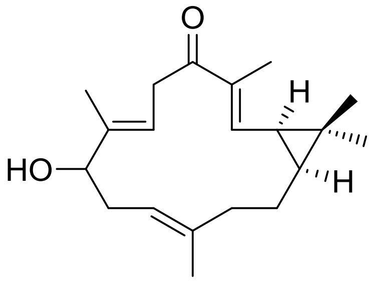

9-hydroxy-5-keto-casbene **(6)**

HMBC

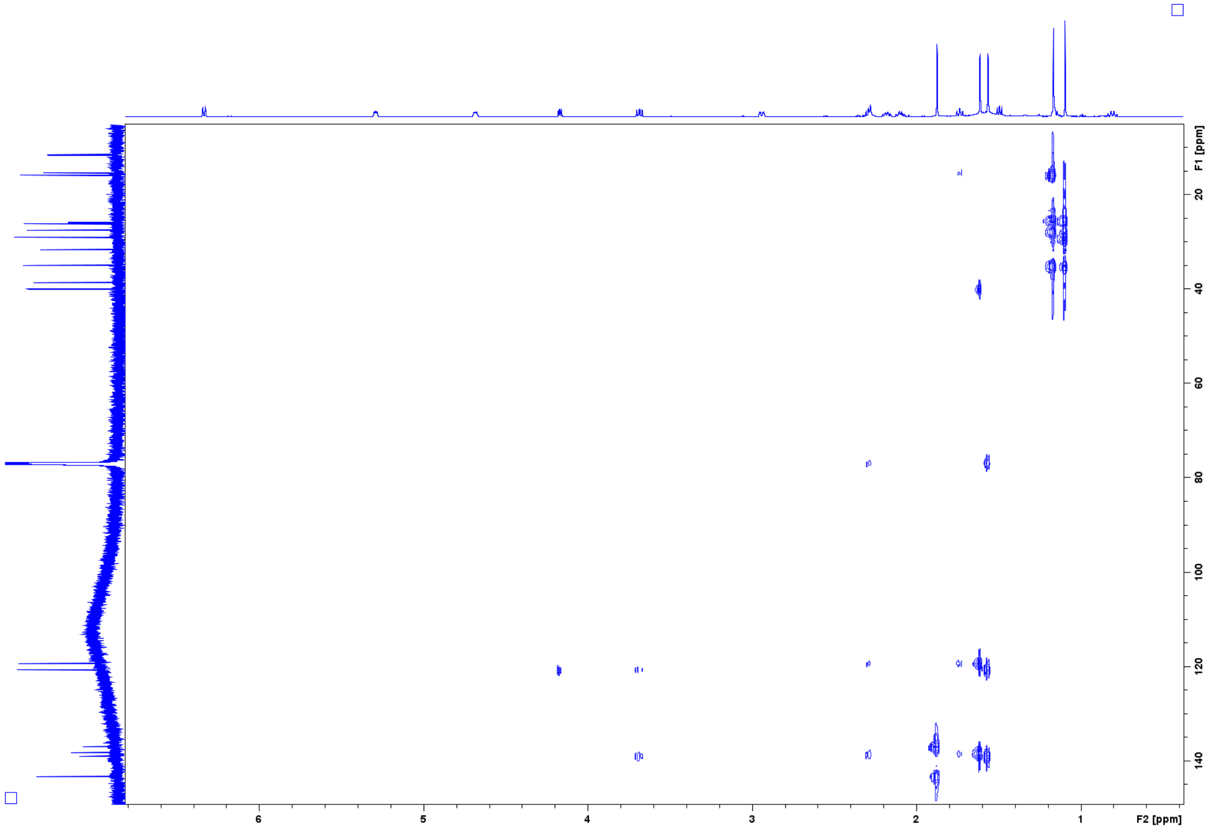

Supporting Figure S10 continued – NMR data for 9-hydroxy-5-keto-casbene

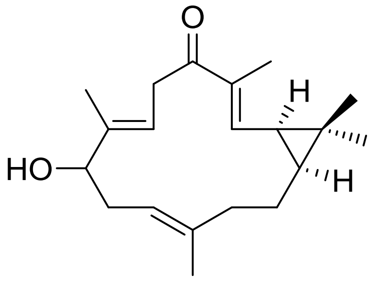

9-hydroxy-5-keto-casbene **(6)**

^{1}H-^{1}H COSY

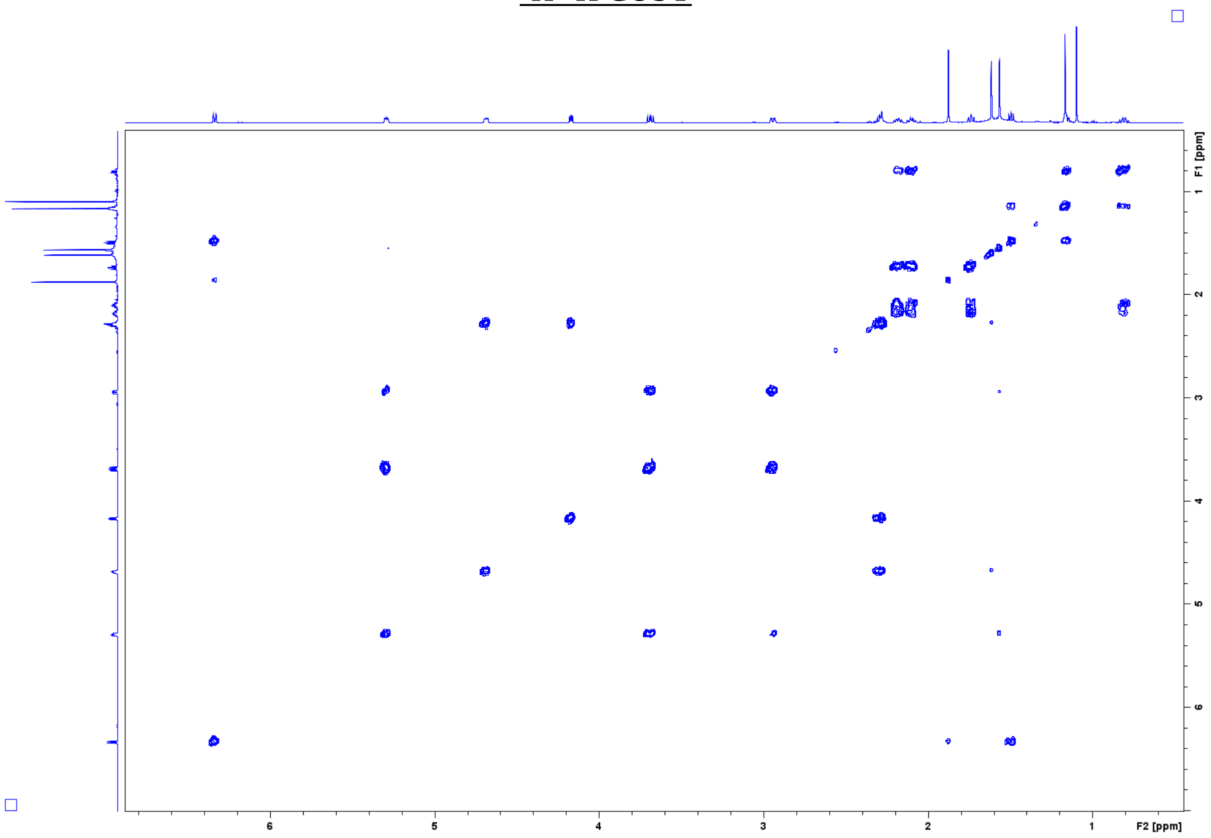

Supporting Figure S10 continued – NMR data for 9-hydroxy-5-keto-casbene

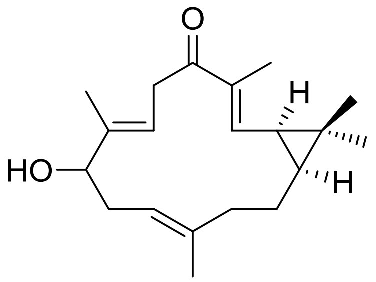

9-hydroxy-5-keto-casbene **(6)**

NOESY

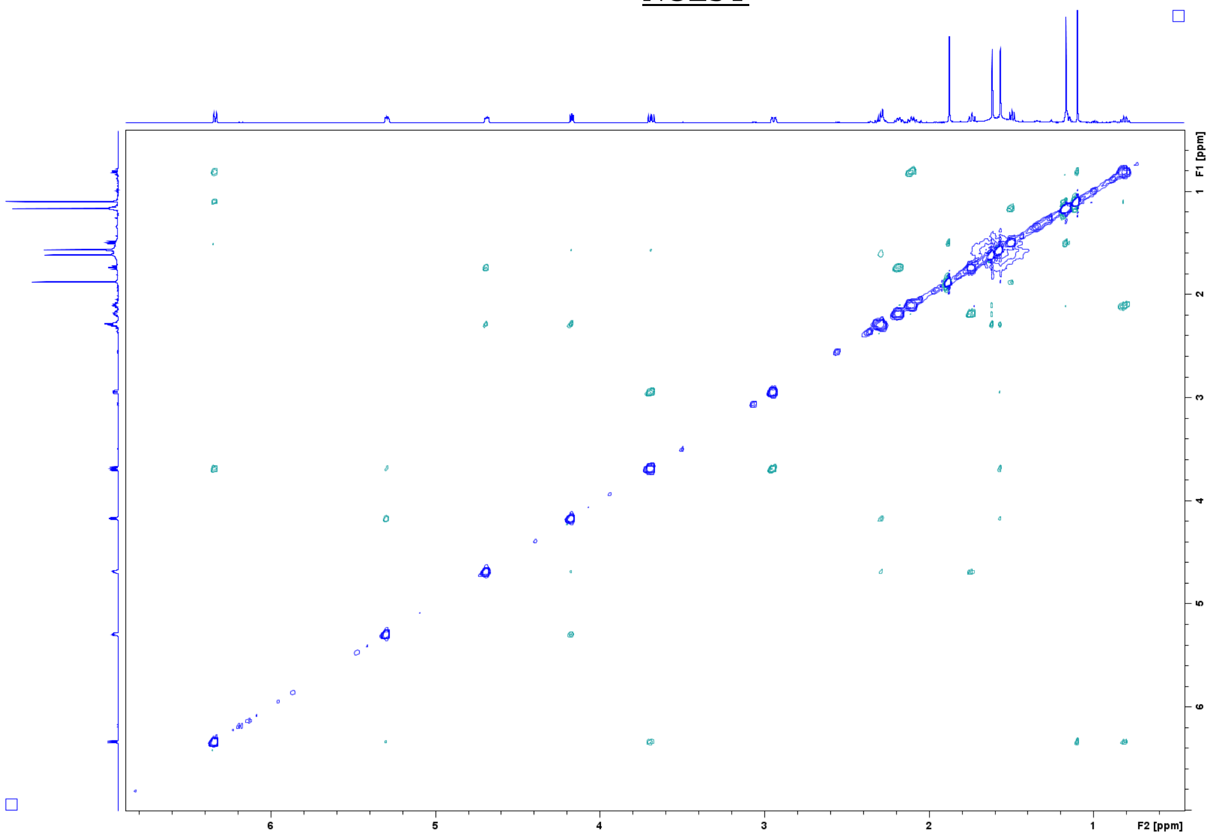

Supporting Figure S11 – NMR data for jolkinol C and *epi*-jolkinol C

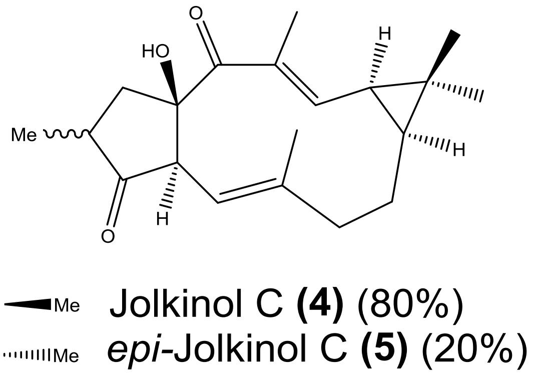

^{1}H NMR (700 MHz; * = ^{1}H resonances resolved for *epi*-jolkinol C (**5**))

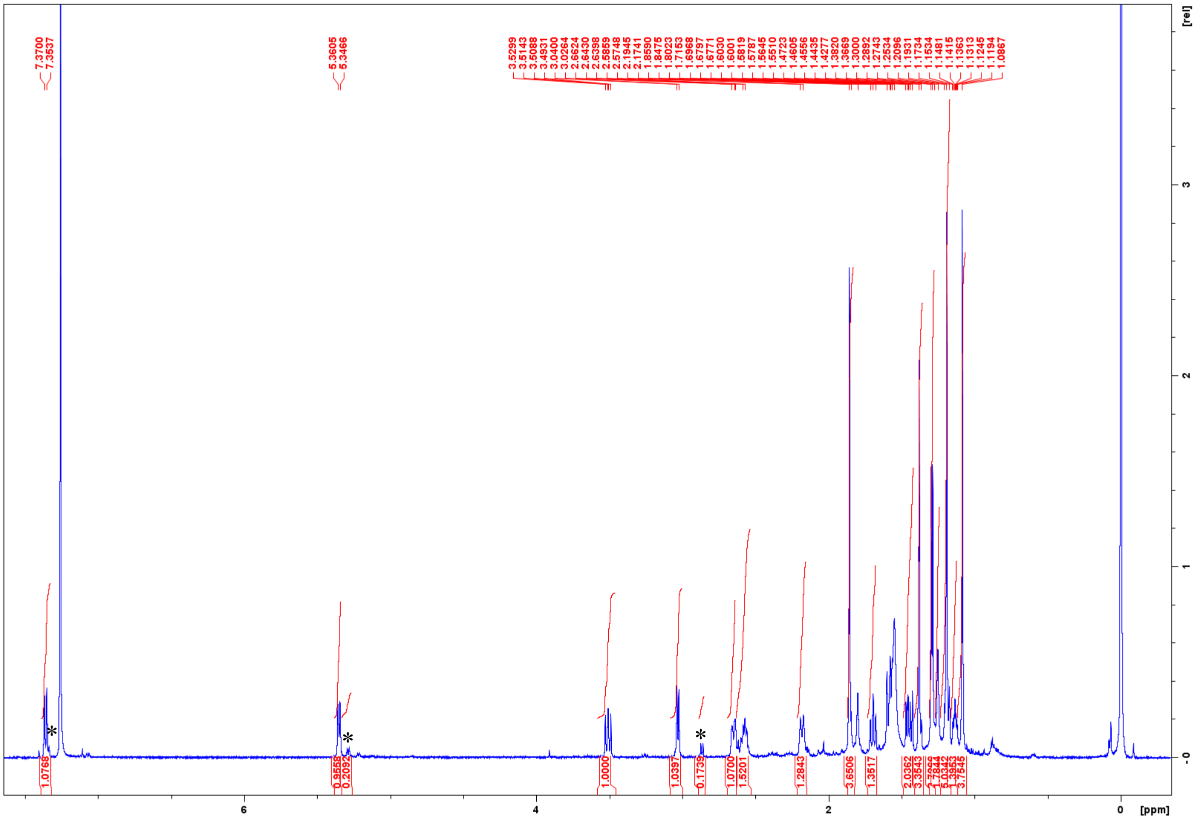

Supporting Figure S11 – NMR data for jolkinol C and *epi*-jolkinol C

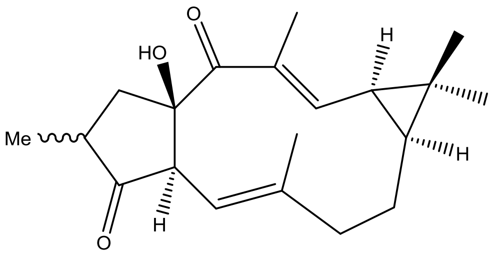

Me Jolkinol C **(4)** (80%)

Me *epi*-Jolkinol C **(5)** (20%)

<u>^{13}C NMR (175 MHz, * = ^{13}C resonances resolved for *epi*-jolkinol C (**5**); sample contains residual d_6-acetone)</u>

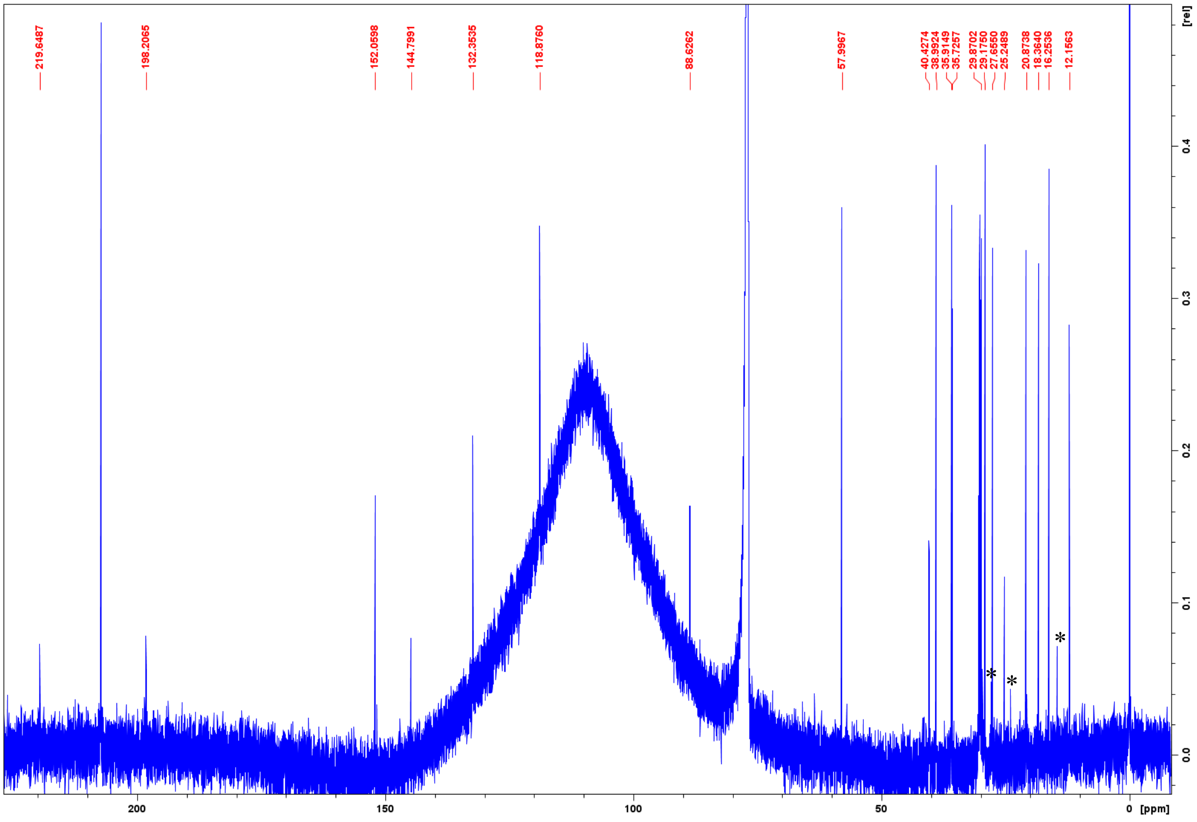

Supporting Figure S11 continued – NMR data for jolkinol C and *epi*-jolkinol C

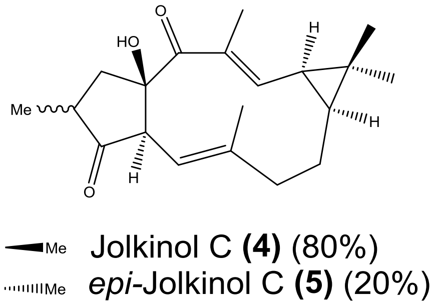

Edited-HSQC (* = ^{13}C-^{1}H correlations resolved for *epi*-jolkinol C (**5**))

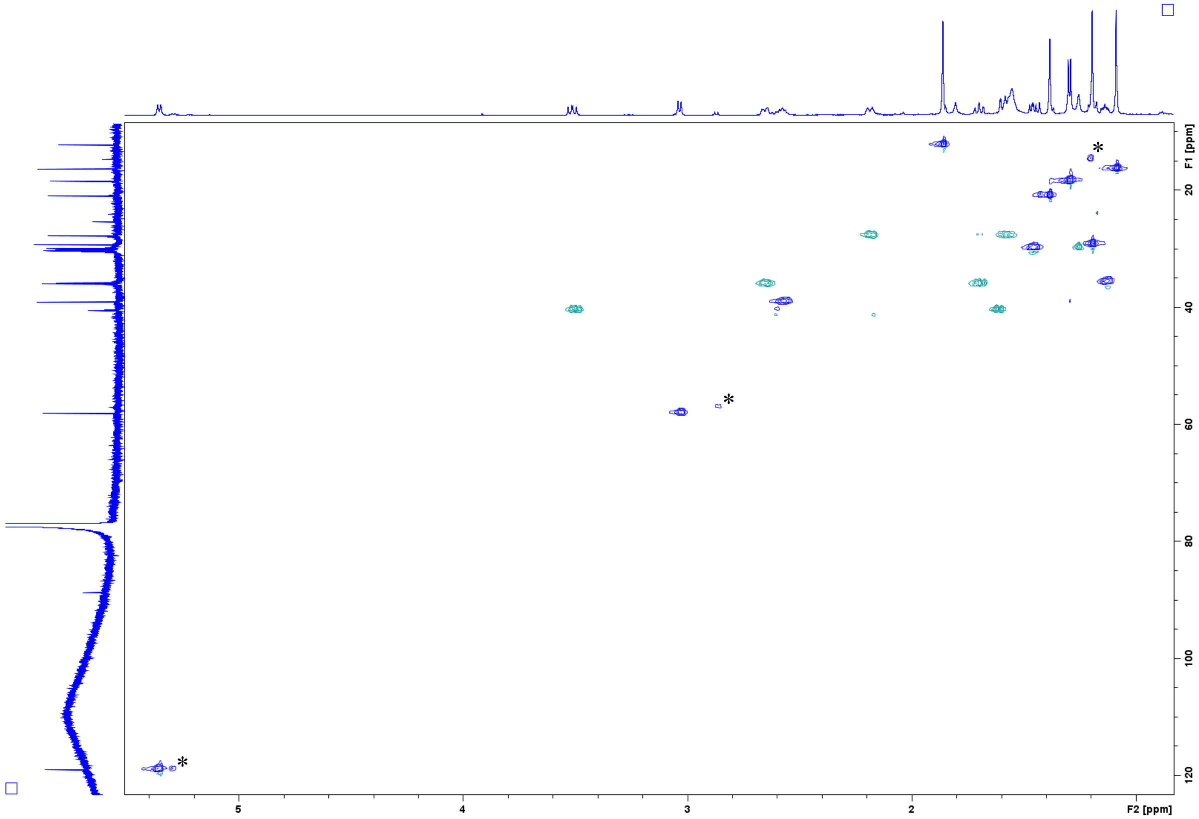

Supporting Figure S11 continued – NMR data for jolkinol C and *epi*-jolkinol C

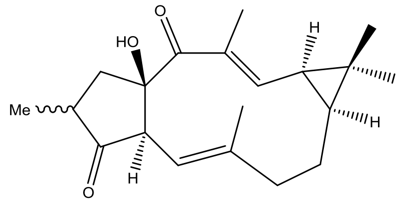

Me Jolkinol C **(4)** (80%)
Me *epi*-Jolkinol C **(5)** (20%)

<u>HMBC</u>

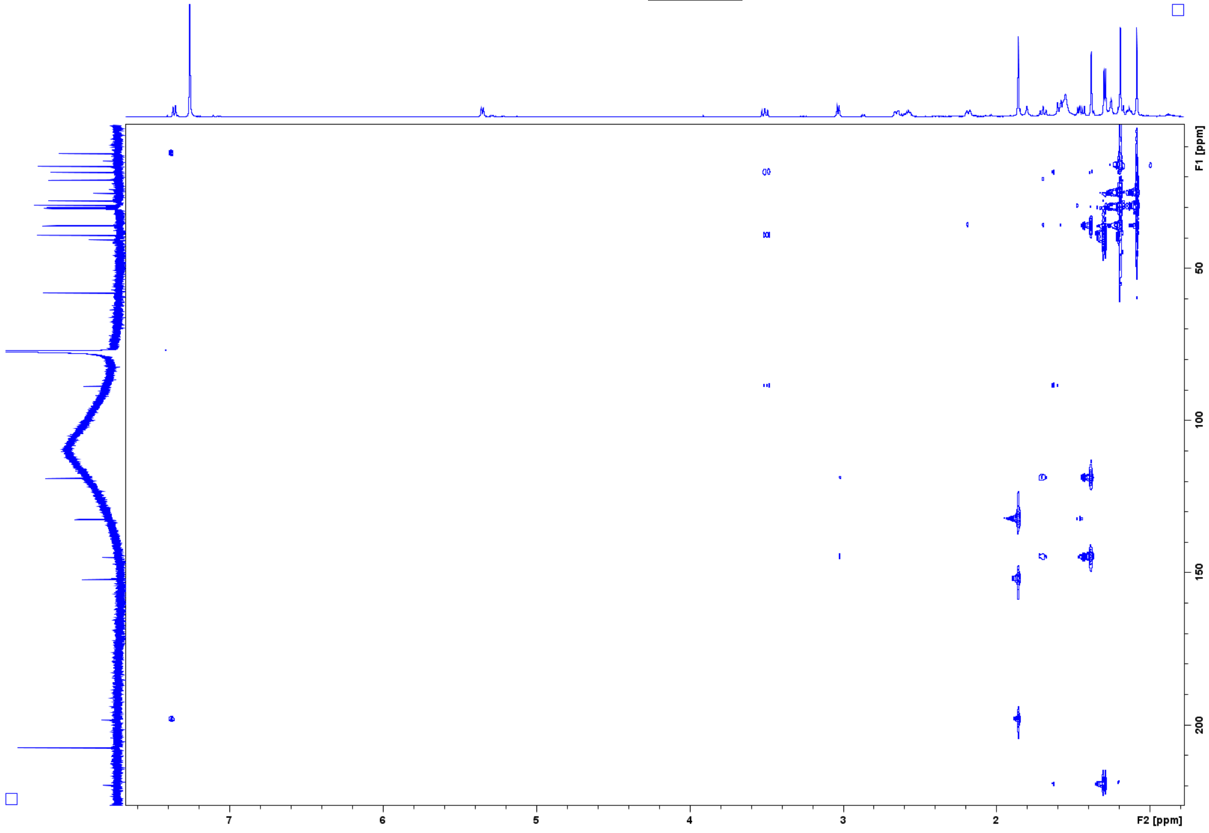

Supporting Figure S11 continued – NMR data for jolkinol C and *epi*-jolkinol C

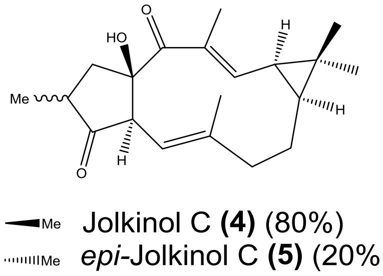

Jolkinol C **(4)** (80%)

epi-Jolkinol C **(5)** (20%)

^{1}H-^{1}H COSY (* = ^{1}H-^{1}H correlation resolved for *epi*-jolkinol C (**5**))

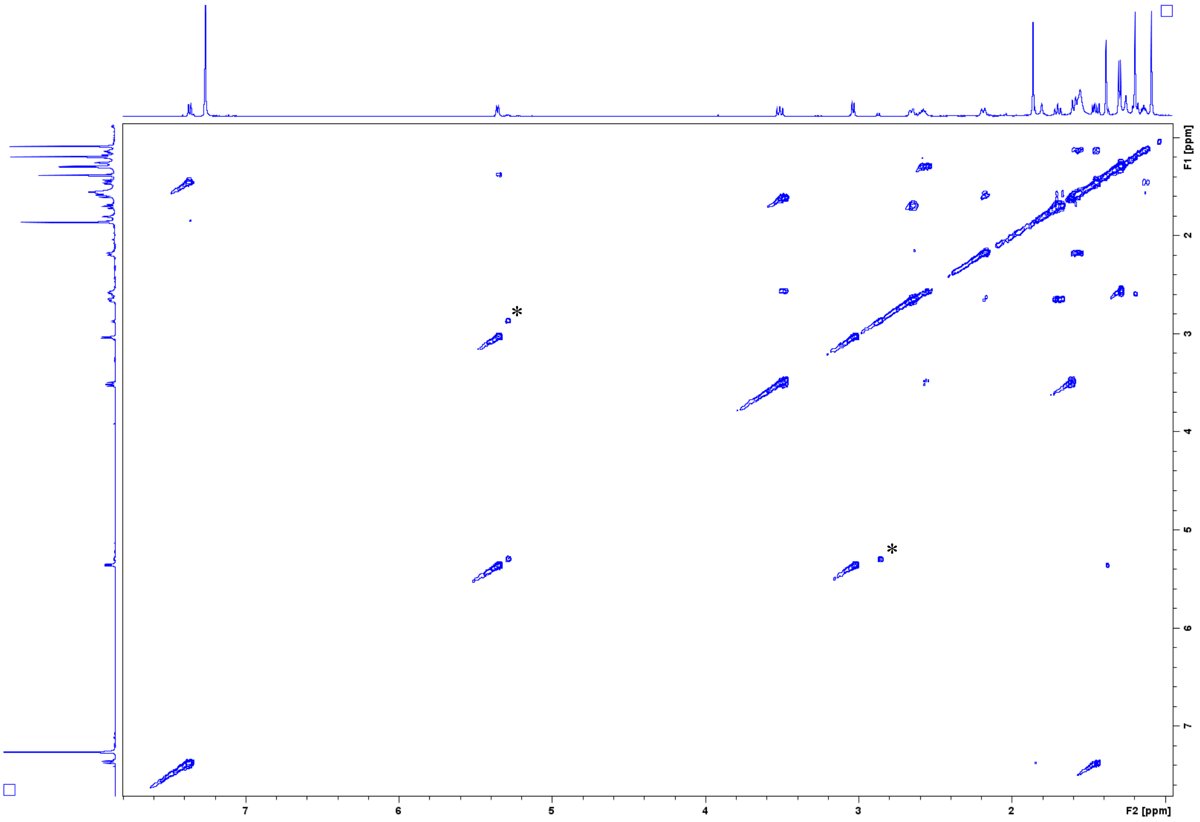

Supporting Figure S11 continued – NMR data for jolkinol C and *epi*-jolkinol C

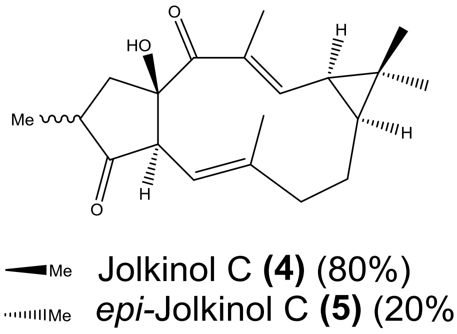

Jolkinol C **(4)** (80%)

epi-Jolkinol C **(5)** (20%)

<u>NOESY</u>

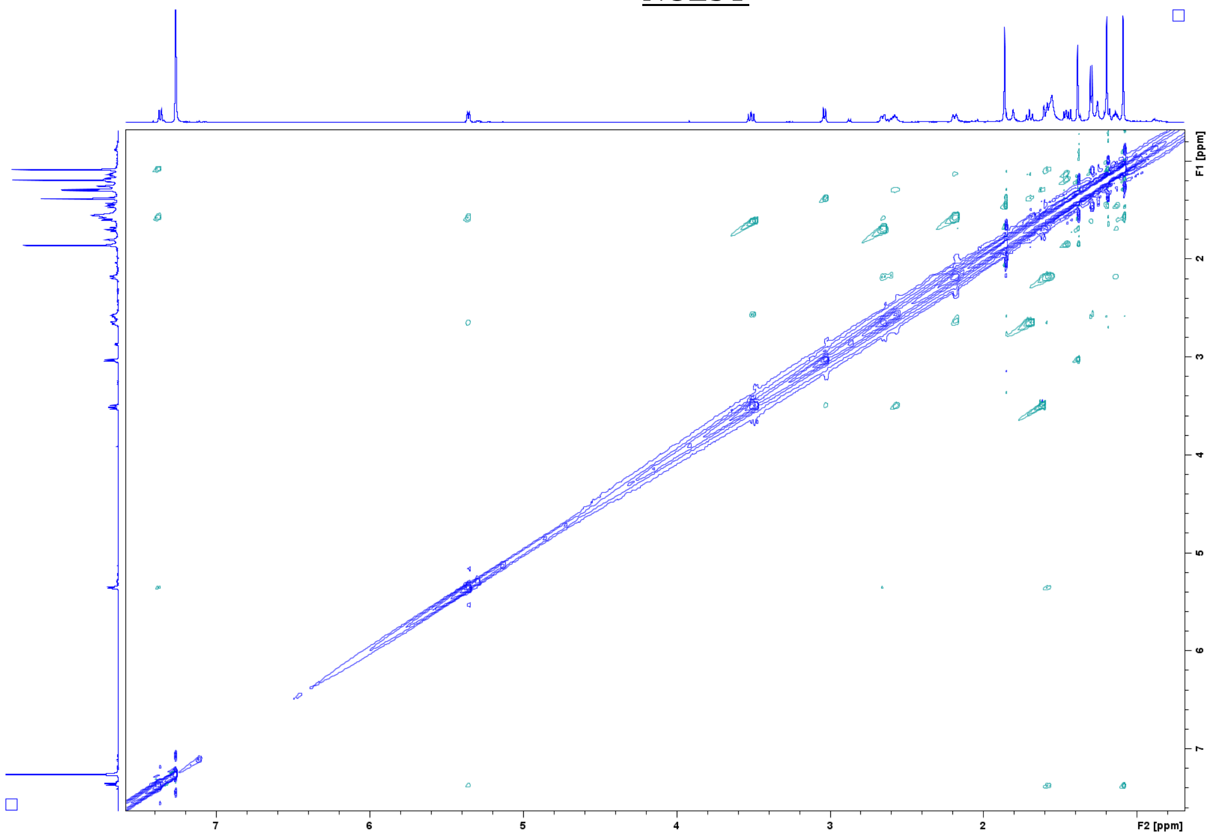

Supporting Figure S11 continued – NMR data for jolkinol C and *epi*-jolkinol C

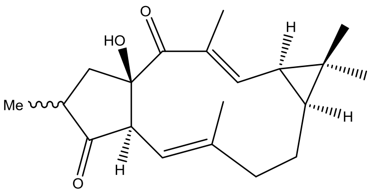

epi-Jolkinol C legend: ''''''''Me *epi*-Jolkinol C **(5)** (65%)

Me Jolkinol C **(4)** (35%)

^{13}C NMR (175 MHz, * = ^{13}C resonances resolved for Jolkinol C (**5**); sample contains residual formic acid and methanol)

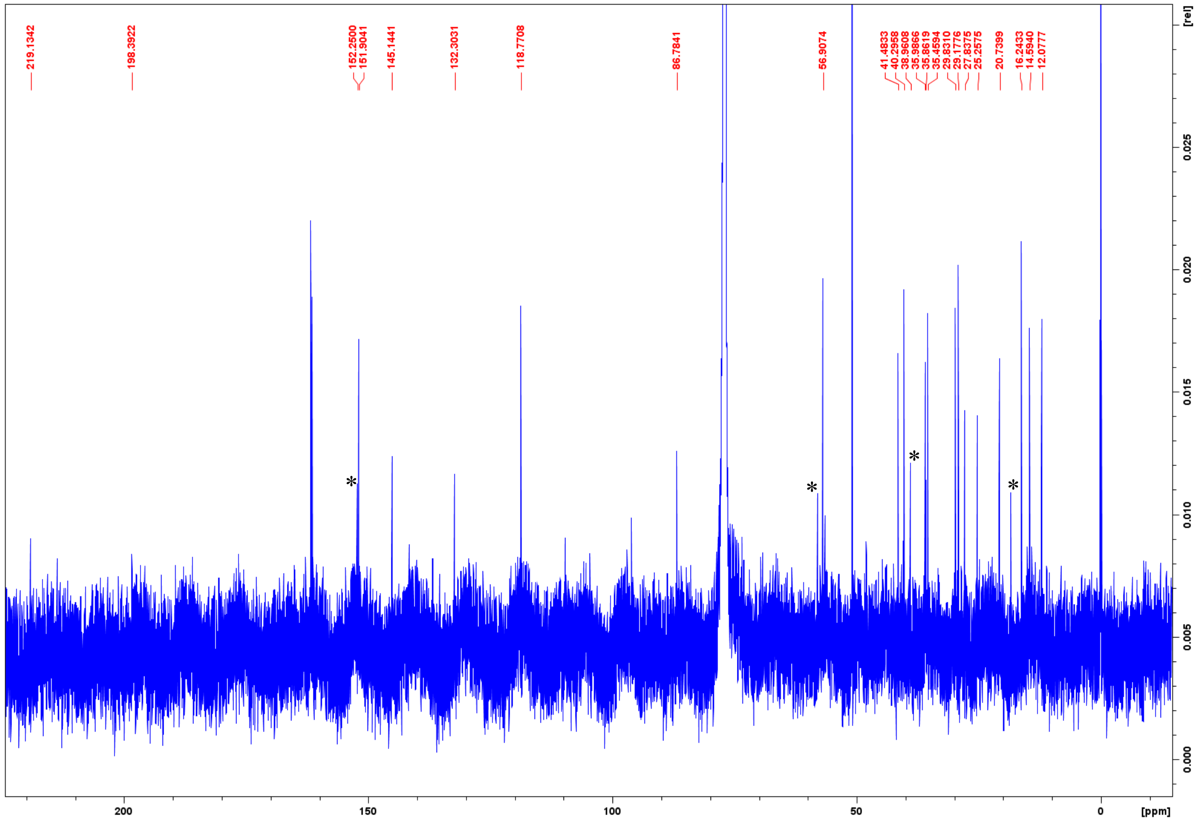

Supporting Figure S11 continued – NMR data for jolkinol C and *epi*-jolkinol C

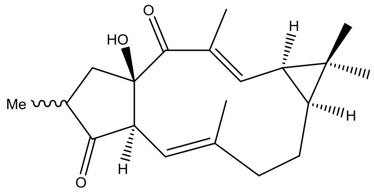

Me *epi*-Jolkinol C **(5)** (65%)

Me Jolkinol C **(4)** (35%)

<u>Edited-HSQC (* = ^{13}C-^{1}H correlations resolved for Jolkinol C (**4**))</u>

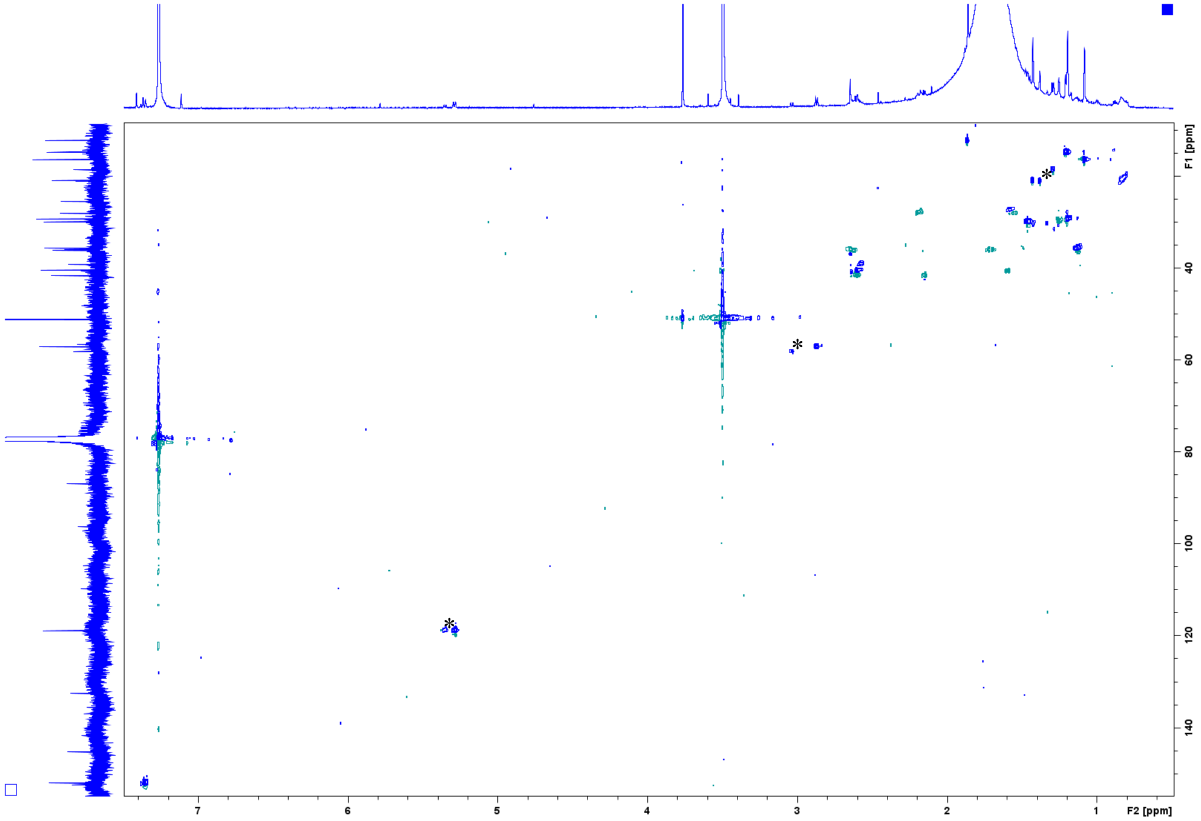

Supporting Figure S11 continued – NMR data for jolkinol C and *epi*-jolkinol C

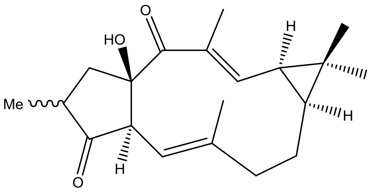

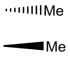

epi-Jolkinol C **(5)** (65%)

Jolkinol C **(4)** (35%)

<u>HMBC</u>

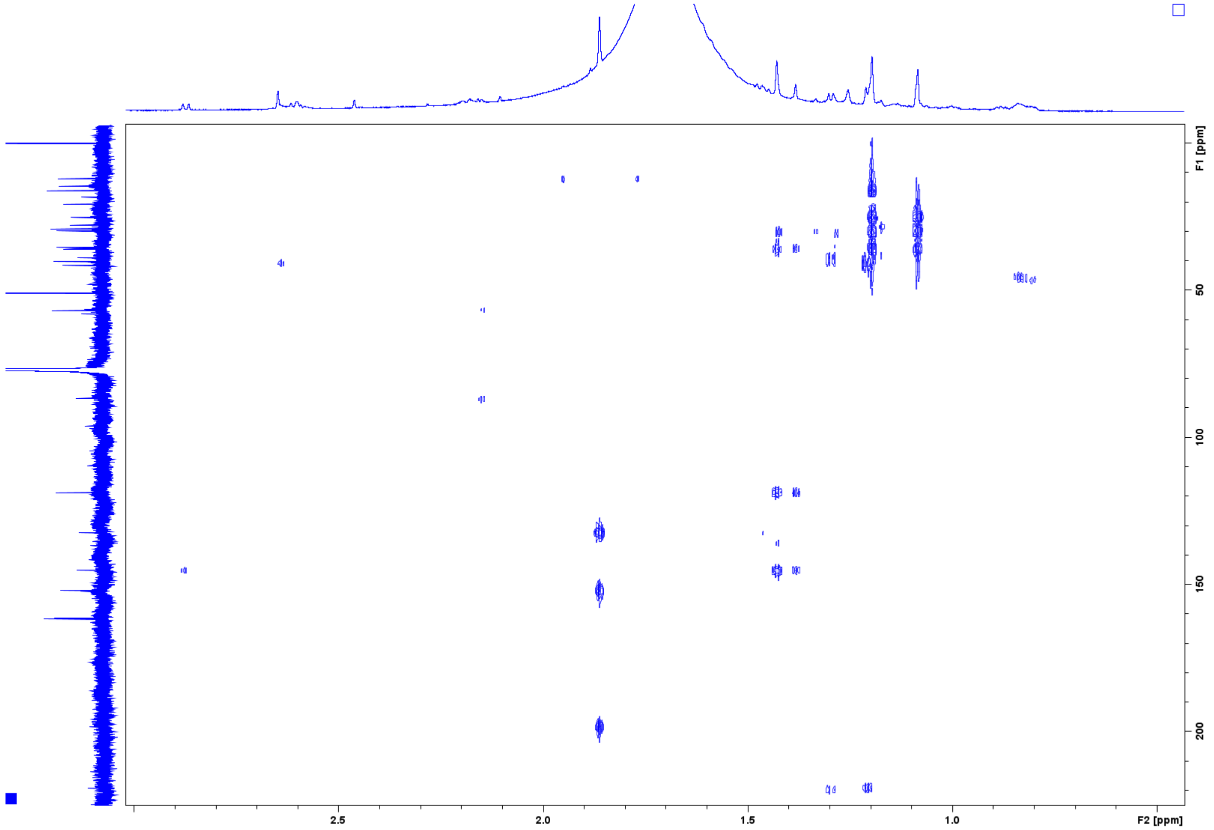

Supporting Figure S11 continued – NMR data for jolkinol C and *epi*-jolkinol C

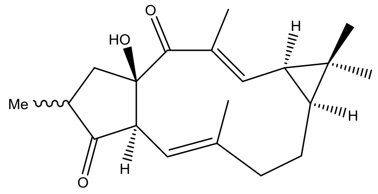

epi-Jolkinol C **(5)** (65%)

Jolkinol C **(4)** (35%)

^{1}H-^{1}H COSY (* = ^{1}H-^{1}H correlation resolved for Jolkinol C (**4**))

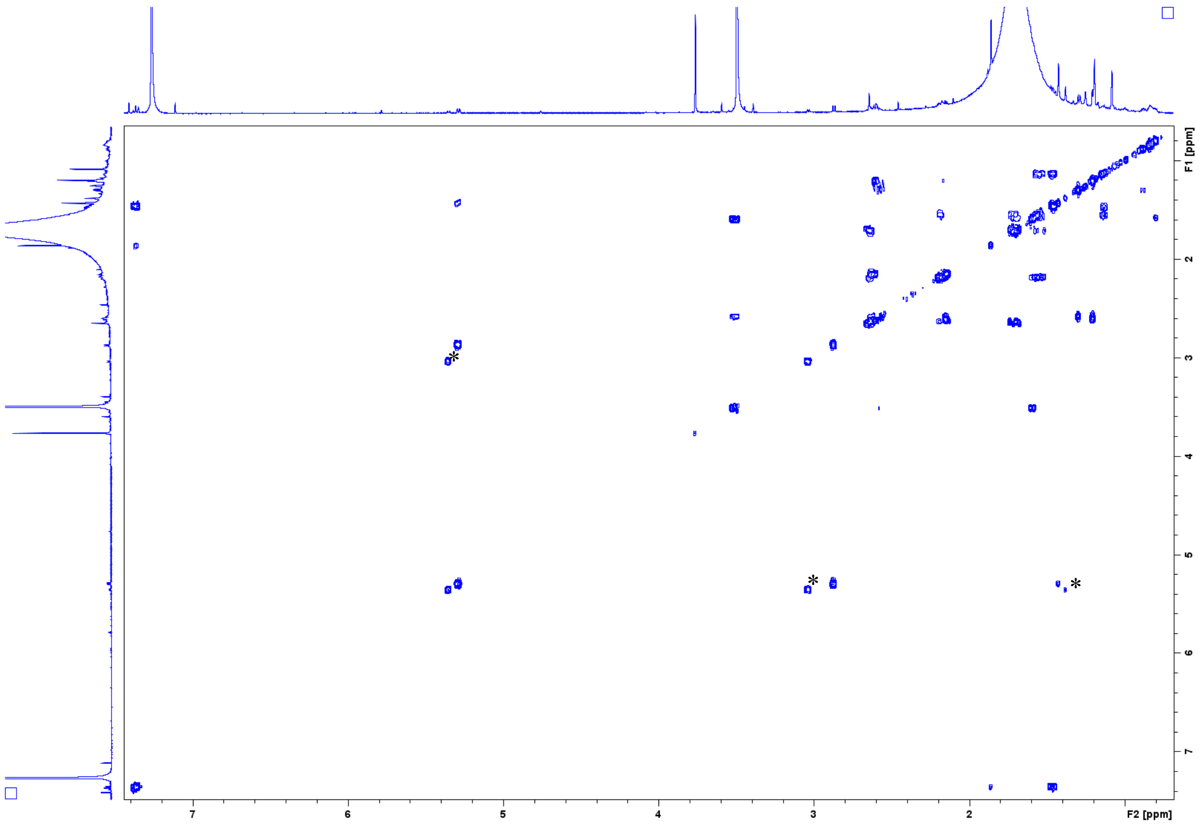

Supporting Figure S11 continued – NMR data for jolkinol C and *epi*-jolkinol C

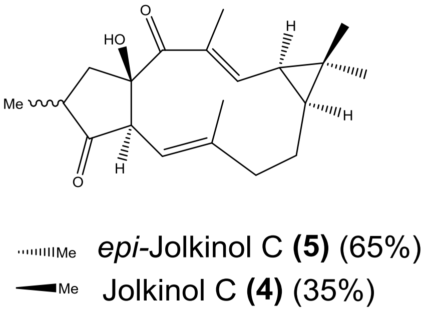

NOESY

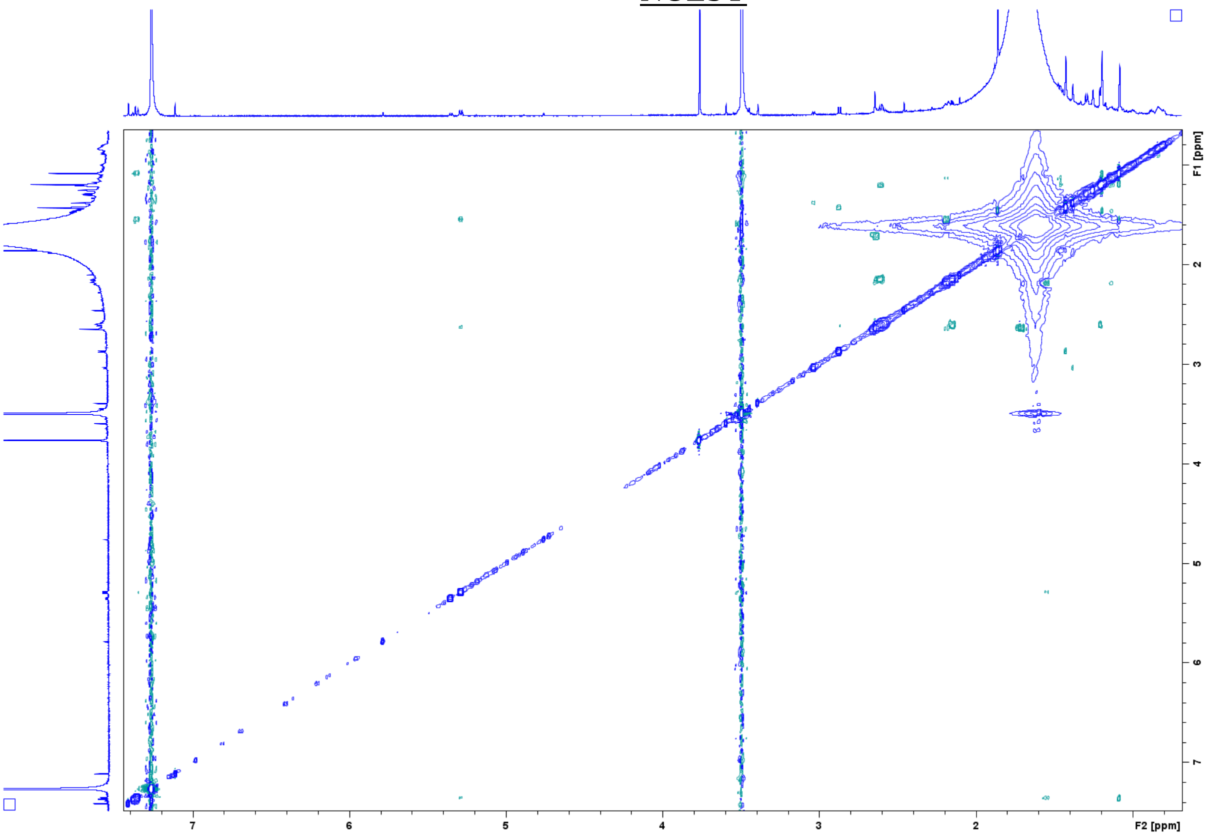

Supporting Figure S12 – NMR data for (3*E*, 6*E*, 11*E*)-8-hydroxy-casba-3,6,11-trien-5,9-dione

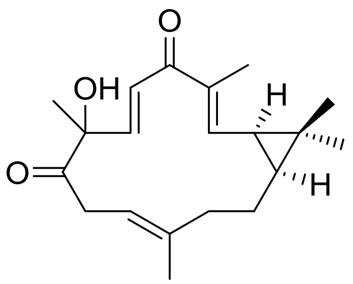

3*E*, 6*E*, 11*E*)-8-hydroxy-
casba-3,6,11-trien-5,9-dione **(7)**

^{1}H NMR (700 MHz)

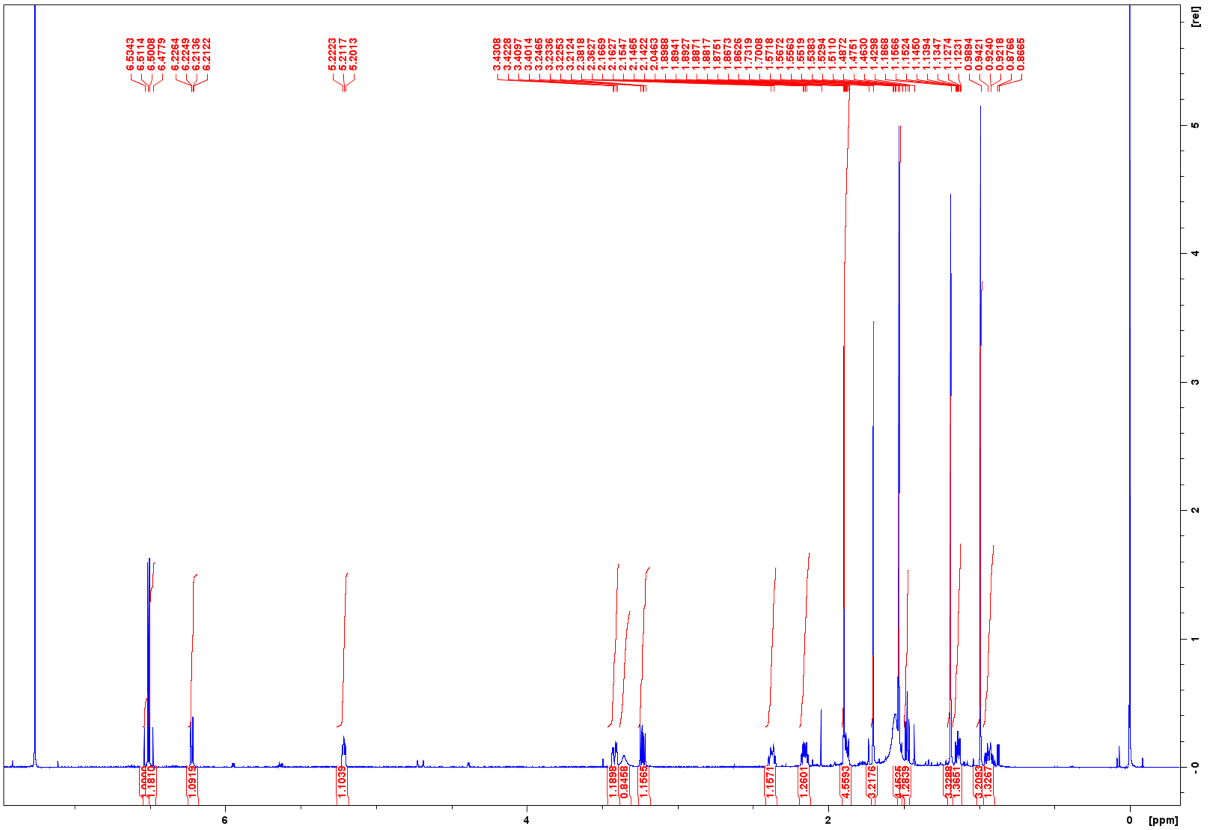

Supporting Figure S12 – NMR data for (3*E*, 6*E*, 11*E*)-8-hydroxy-casba-3,6,11-trien-5,9-dione

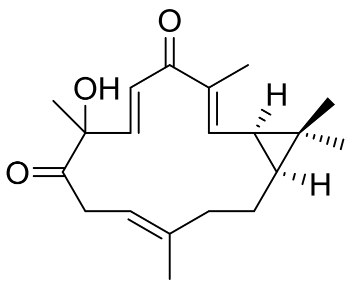

3*E*, 6*E*, 11*E*)-8-hydroxy-
casba-3,6,11-trien-5,9-dione **(7)**

<u>^{13}C NMR (175 MHz)</u>

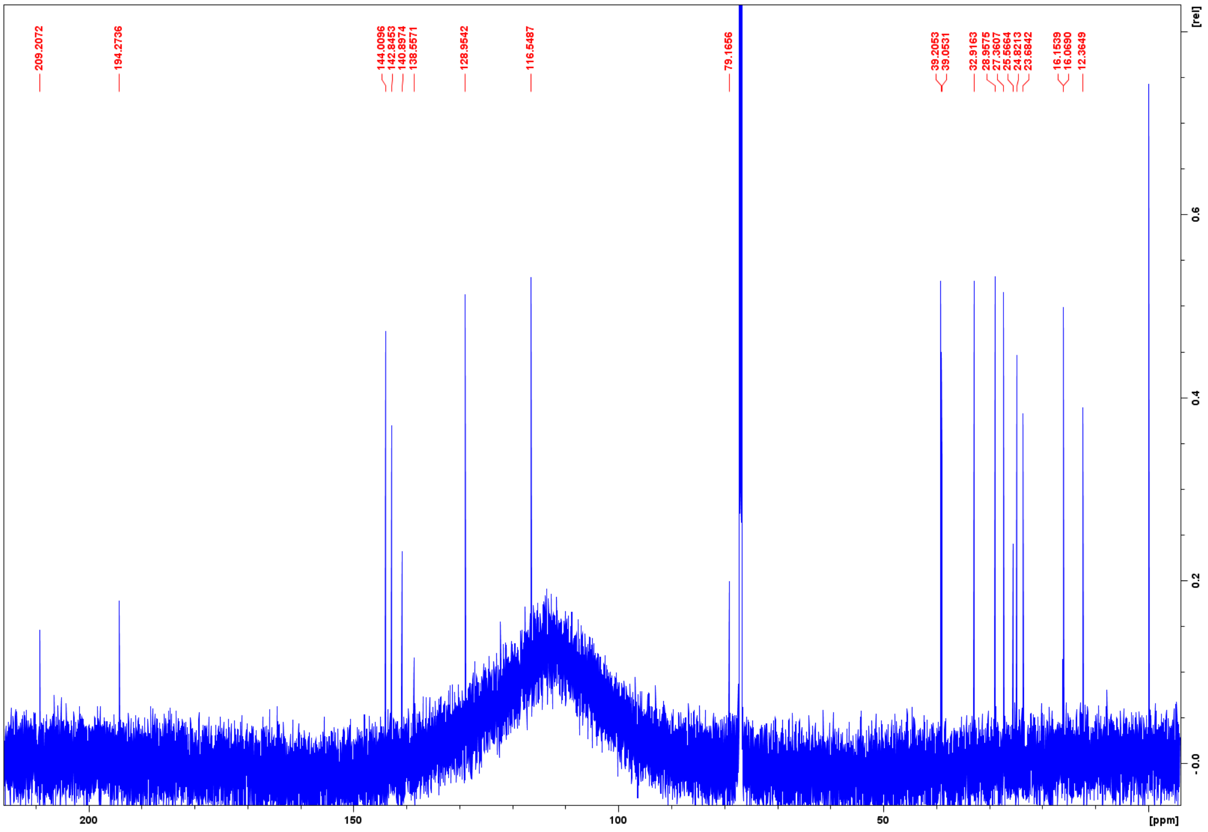

Supporting Figure S12 – NMR data for (3*E*, 6*E*, 11*E*)-8-hydroxy-casba-3,6,11-trien-5,9-dione

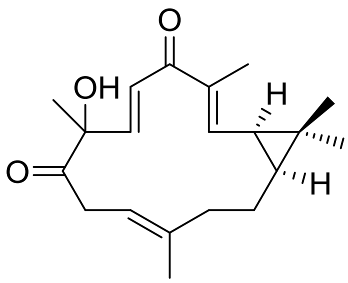

3*E*, 6*E*, 11*E*)-8-hydroxy-
casba-3,6,11-trien-5,9-dione **(7)**

Edited-HSQC

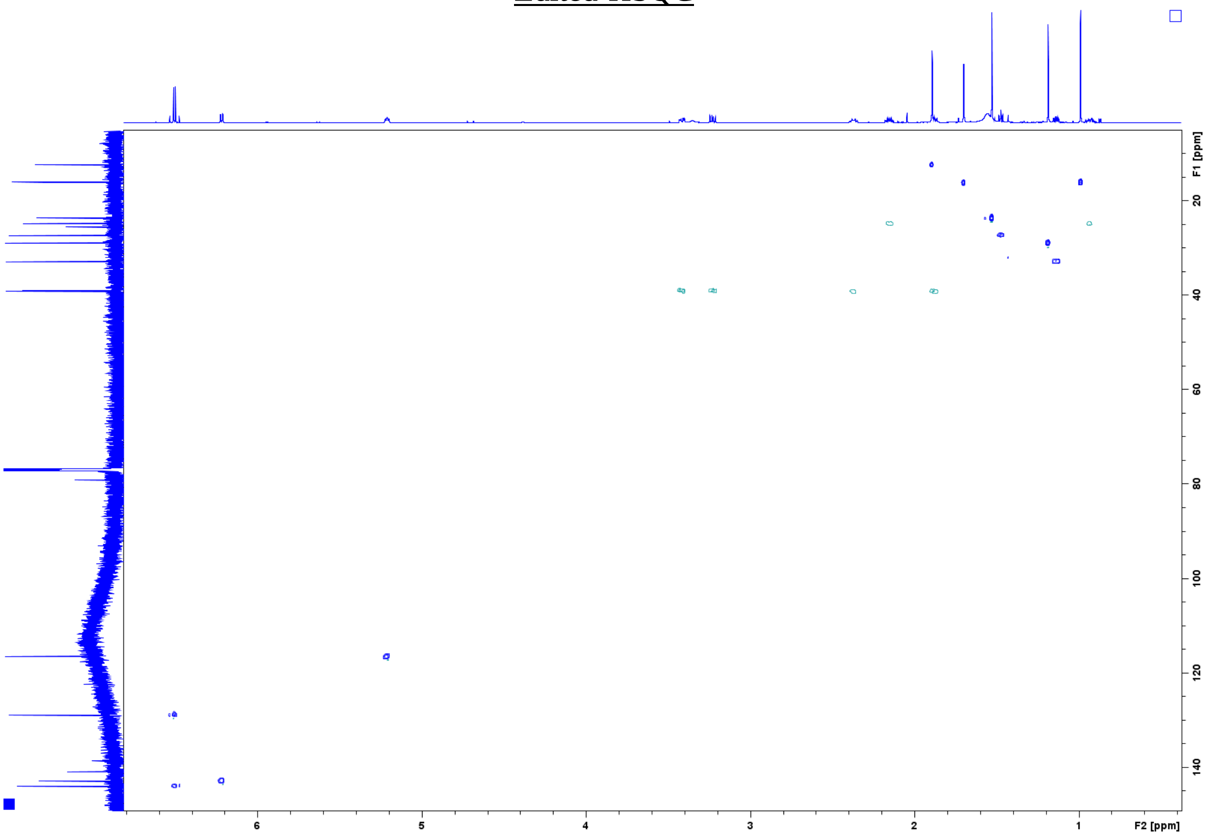

Supporting Figure S12 – NMR data for (3*E*, 6*E*, 11*E*)-8-hydroxy-casba-3,6,11-trien-5,9-dione

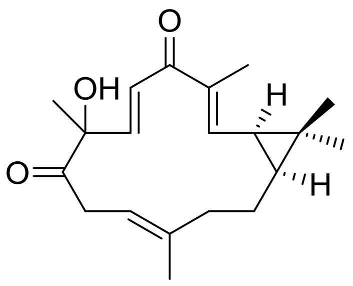

3*E*, 6*E*, 11*E*)-8-hydroxy-casba-3,6,11-trien-5,9-dione **(7)**

HMBC

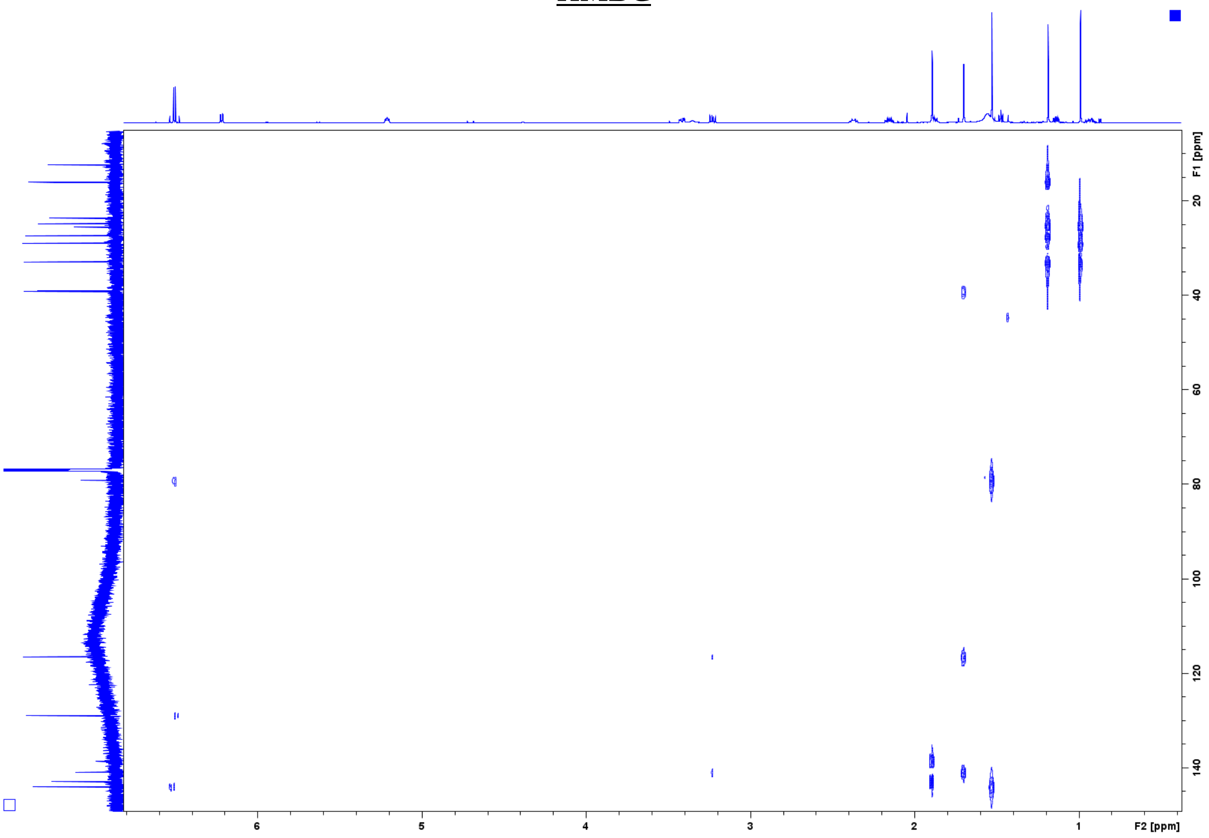

Supporting Figure S12 – NMR data for (3*E*, 6*E*, 11*E*)-8-hydroxy-casba-3,6,11-trien-5,9-dione

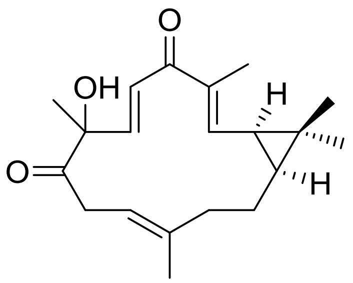

3*E*, 6*E*, 11*E*)-8-hydroxy-casba-3,6,11-trien-5,9-dione **(7)**

<u>^{1}H-^{1}H COSY</u>

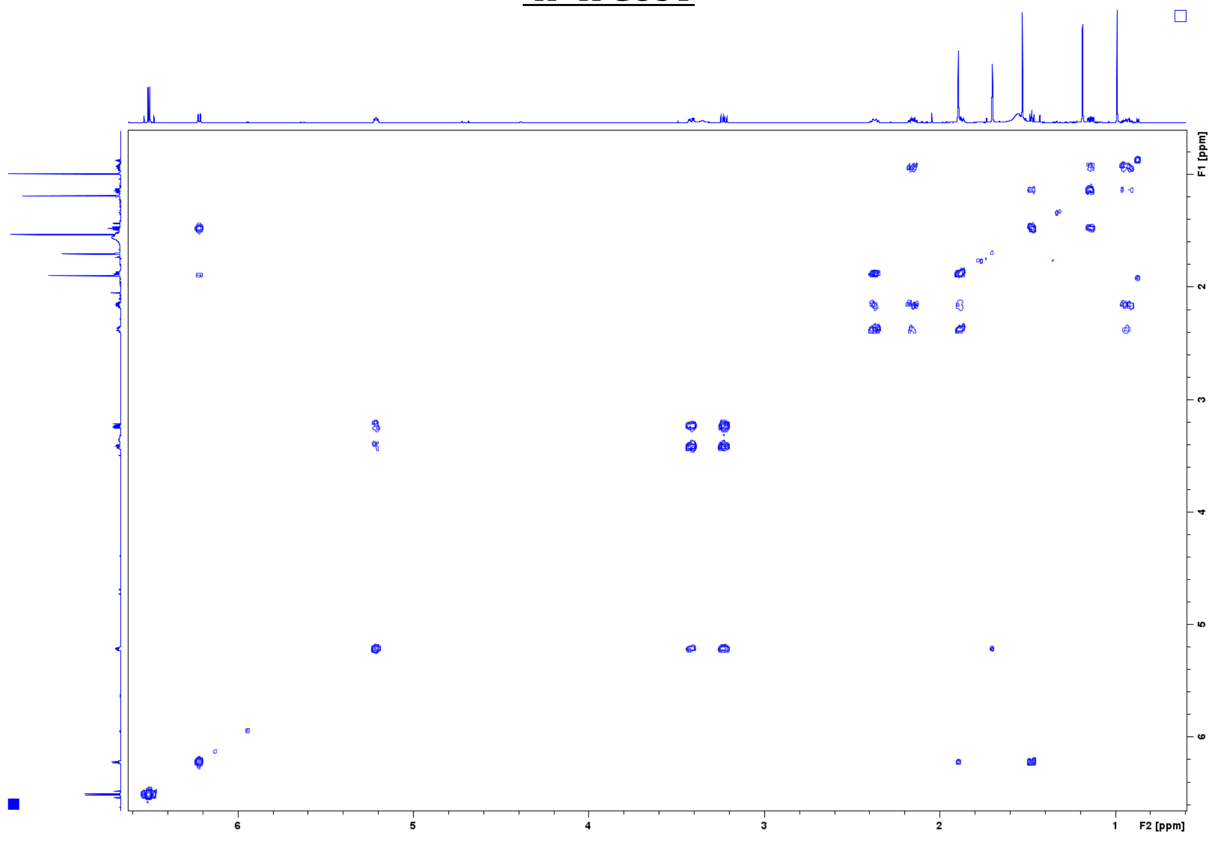

Supporting Figure S12 – NMR data for (3*E*, 6*E*, 11*E*)-8-hydroxy-casba-3,6,11-trien-5,9-dione

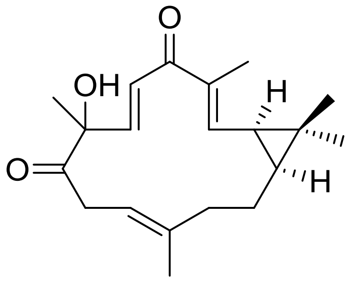

3*E*, 6*E*, 11*E*)-8-hydroxy-casba-3,6,11-trien-5,9-dione **(7)**

NOESY

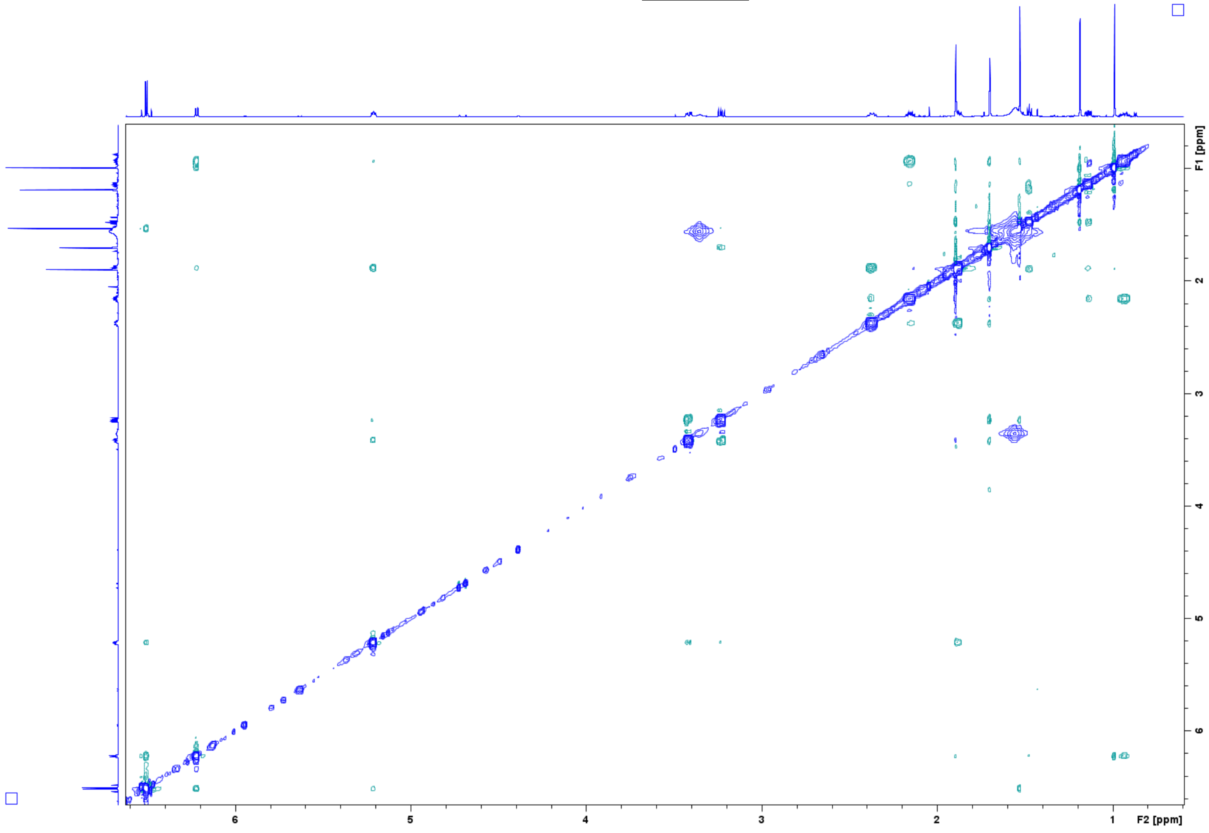

Supporting Table S1 – Sequences of primers used for qPCR analysis of gene expression on *J. curcas* genome scaffold 123.

Gene ID	Gene	Position on scaffold 123	Forward	Reverse	Annealing temperature
105629799	2-alkenal reductase like	(40398..41923)	5'-CCCAGAAGGAAGTATGCCCG-3'	5'-CTTTGCAAGTTGCCCAACGA-3'	60 °C
105629800	2-alkenal reductase like	(70021..71581)	5'-CTCCAAGTCCCAGAAGGAAGT-3'	5'-CGGGAAAATCTAGGCTGAGTGT-3'	65 °C
105629801	2-alkenal reductase like	(84833..87073)	5'-GCAGTGTTGCTGAATATGAGGC-3'	5'-TCCCGCAATGAATCTTGTCTGA-3'	65 °C
105629802	CYP726A24	(104379..106016)	5'-AGCTCGCAGGCTACCAATTT-3'	5'-CTTCTTTGGCCATTTCCGGC-3'	65 °C
105629803	2-alkenal reductase like	(109911..111959)	5'-CTGGGCATCCTTTTGCACCA-3'	5'-TCTTGAAGTCTGGCGGCG-3'	60 °C
105629805	CYP726A23	(127836..129469)	5'-TAACAGGAAGGCGGCAGTTC-3'	5'-CTGCCAGCCCCAAACATTTC-3'	63 °C
105629806	CYP71D495	(134771..137289)	5'-TGCTGGGATAAACAGTAAGGAGG-3'	5'-ATGACGTGTCACTACCAGCG-3'	57 °C
105629816	CYP71D496	(149297..150867)	5'-CAGCTCGGCGAAATTACCAC-3'	5'-GTGCGAGTGCGATATCTGTG-3'	65 °C
105629807	Short-chain alcohol dehydrogenase	(152208..153156)	5'-GGGTTTGAGCGAACAGCAAG-3'	5'-AGCAAGGTACAAAGCAGCCT-3'	65 °C
105629814	Monoterpene synthase	(174139..176533)	5'-CTCAAACCCAGCTTTTGCCC-3'	5'-TCGTTGGGGTTATTGGCACA-3'	62 °C
105629808	Monoterpene synthase	(191855..195188)	5'-ATGGCGGGTTCGGATCTTAC-3'	5'-GACATTGCTTGTTGAGCCGT-3'	65 °C
105629820	Monoterpene synthase	(209488..211654)	5'-GCTACTGCGTACCTGCTGAT-3'	5'-AGGGCCACTAAAAACTCGGG-3'	65 °C
105629809	CYP726A35	(237179..240418)	5'-AACATAAAGCCGACAGGGCA -3'	5'-CTGCCTGCGCCAAATGTATC-3'	59 °C
105629810	Casbene synthase 3	(246989..249381)	5'-CCTAGTGGCAAGCTGAACGA-3'	5'-TGGACGAGTGTCTGTCTCTGA-3'	65 °C
105629821	Casbene synthase 2	(252084..255566)	5'-ACATGTTTAATGGCGGGGTT-3'	5'-TTCGCCTCCAGCTTGATTGA-3'	55 °C
105629811	Casbene synthase 1	(259916..262727)	5'-GGTCCACAGAAGTTGTGCCA-3'	5'-TCAGTTGTGAAGAGTCCGTGT-3'	65 °C
105629812	CYP726A20	(284198..285983)	5'-TTGGGATAGGAGCGAAGCTG-3'	5'-TCGCTTCCAGCACCAAACAT-3'	58 °C
105629813	CYP726A21	(297137..298771)	5'-CTGATCGACCGCTTGTCCTT-3'	5'-CTCCGTACAGCCCAAAACCT-3'	58 °C
Housekeeping gene					
XM_012232498	Actin	n/a	5'-TGCCATCCAGGCCGTTCTATCT-3'	5'-GGAGGATAGCATGTGGAAGAGCG-3'	61 °C

Supporting Table S2 – Sequences of primers used insertion of *J. curcas* cDNA sequences into AgeI and XhoI sites of pEAQ-HT vector

Organism	Gene ID	Annotation	Forward	Reverse
Conventional cloning using restriction digestion with BsaI and ligation into AgeI and XhoI sites of pEAQ-HT				
J. curcas	105629806	CYP71D495	5'-AAAAGGTCTCACCGGAAAAATGCTTTTCTTCATCACCGTACTC-3'	5'-AAAAGGTCTCATCGACTATCTTGAGATTTTACCAACTGCTG-3'
Conventional cloning using restriction digestion AgeI and XhoI into AgeI and XhoI sites of pEAQ-HT				
J. curcas	105629809	CYP726A35	5'-AAAAACCGGTAAAAATGTCGCTGCAACCAGCAATTTTAC-3'	5'-AAAACTCGAGTCATAATGCTTTTAAGTGTGGGCAC-3'
Gibson cloning into the AgeI and XhoI sites of pEAQ-HT				
J. curcas	105629812	CYP726A20	5'-TATTCTGCCCAAATTCGCGAAAAAAATGGAACACCAAATCCTCTCATTT-3'	5'-TGAAACCAGAGTTAAAGGCCTTAGGGACGGAATGGAATGGGG-3'
A. thaliana	At4g15560	DXS	5'-TATTCTGCCCAAATTCGCGACCGGTAAAAATGGCTTCTTCTGCATTTG-3'	5'-TGAAACCAGAGTTAAAGGCCTCGAGTCAAAACAGAGCTTCCCTTG-3'
A. thaliana	At4g36810	GGPPS11	5'-TATTCTGCCCAAATTCGCGACCGGTAAAAATGGCTTCAGTGACTCTAG-3'	5'-TGAAACCAGAGTTAAAGGCCTCGAGTCAGTTCTGTCTATAGGCAATG-3'

Supporting Table S3 – Primers used for creation of GFP fusion constructs in pEAQ-HT via Gibson Assembly

**Fragment**	**Domain**	**Forward**	**Reverse**
Casbene synthase plastidial transit sequence.			
Fragment 1	AA 1-72	5'-CTGCCCAAATTCGCGACCGGTAAAAATGGCAATGCAACCTGCA-3'	5'-TTGCTCACCCATACAGTAGGAGGAAAGTAG-3'
Fragment 2	eGFP	5'-CTGTATGGGTGAGCAAGGGCGAGGAG-3'	5'-GAAACCAGAGTTAAAGGCCTTACTTGTACAGCTCGTCCATG-3'
CYP726A35 plastidial transit sequence.			
Fragment 1	AA 1-93	5'-CTGCCCAAATTCGCGACCGGTAAAAATGTCGCTGCAACCAGCA-5'	5'-TTGCTCACGAATATTTTGGTAAGACTTGTGGTAGTTG-3'
Fragment 2	eGFP	5'-CAAAATATTCGTGAGCAAGGGCGAGGAG-3'	5'-GAAACCAGAGTTAAAGGCCTTACTTGTACAGCTCGTCCATG-3'
Fragment 1	AA 1-80	5'-CTGCCCAAATTCGCGACCGGTAAAAATGGAACACCAAATCCTC-3'	5'-TTGCTCACGAAAGGAACTTGCCCAAG-3'
Fragment 2	eGFP	5'-GTTCCTTTCGTGAGCAAGGGCGAGGAG-3'	5'-GAAACCAGAGTTAAAGGCCTTACTTGTACAGCTCGTCCATG-3'

Refences to Supporting information.

[1] A. J. King, G. D. Brown, A. D. Gilday, T. R. Larson, I. A. Graham, *Plant Cell* **2014**, *26*, 3286-3298.
[2] A. Untergasser, H. Nijveen, X. Rao, T. Bisseling, R. Geurts, J. A. M. Leunissen, *Nucl Acids Res* **2007**, *35*, W71 - 74.
[3] M. W. Pfaffl, *Nucl. Acids Res.* **2001**, *29*, 6.
[4] J. M. Ruijter, C. Ramakers, W. M. H. Hoogaars, Y. Karlen, O. Bakker, M. J. B. van den Hoff, A. F. M. Moorman, *Nucl. Acids Res.* **2009**, *37*, 12.
[5] R. Hofgen, L. Willmitzer, *Nucl. Acids Res.* **1988**, *16*, 9877.
[6] O. Emanuelsson, H. Nielsen, G. V. Heijne, *Protein Science* **1999**, *8*, 978-984.
